# Complex Responses to Hydrogen Peroxide and Hypochlorous Acid by the Probiotic Bacterium Lactobacillus reuteri

**DOI:** 10.1128/mSystems.00453-19

**Published:** 2019-09-03

**Authors:** Poulami Basu Thakur, Abagail R. Long, Benjamin J. Nelson, Ranjit Kumar, Alexander F. Rosenberg, Michael J. Gray

**Affiliations:** a Department of Microbiology, School of Medicine, University of Alabama at Birmingham, Birmingham, Alabama, USA; b Auburn University, Auburn, Alabama, USA; c Center for Clinical and Translational Sciences, University of Alabama at Birmingham, Birmingham, Alabama, USA; d Informatics Institute, School of Medicine, University of Alabama at Birmingham, Birmingham, Alabama, USA; University of California, San Francisco

**Keywords:** oxidative stress, probiotics, transcriptomics

## Abstract

Inflammatory diseases of the gut are associated with increased intestinal oxygen concentrations and high levels of inflammatory oxidants, including hydrogen peroxide (H_2_O_2_) and hypochlorous acid (HOCl), which are antimicrobial compounds produced by the innate immune system. This contributes to dysbiotic changes in the gut microbiome, including increased populations of proinflammatory enterobacteria (Escherichia coli and related species) and decreased levels of health-associated anaerobic *Firmicutes* and *Bacteroidetes*. The pathways for H_2_O_2_ and HOCl resistance in E. coli have been well studied, but little is known about how commensal and probiotic bacteria respond to inflammatory oxidants. In this work, we have characterized the transcriptomic response of the anti-inflammatory, gut-colonizing Gram-positive probiotic Lactobacillus reuteri to both H_2_O_2_ and HOCl. L. reuteri mounts distinct but overlapping responses to each of these stressors, and both gene expression and survival were strongly affected by the presence or absence of oxygen. Oxidative stress response in L. reuteri required several factors not found in enterobacteria, including the small heat shock protein Lo18, polyphosphate kinase 2, and RsiR, an L. reuteri-specific regulator of anti-inflammatory mechanisms.

**IMPORTANCE** Reactive oxidants, including hydrogen peroxide and hypochlorous acid, are antimicrobial compounds produced by the immune system during inflammation. Little is known, however, about how many important types of bacteria present in the human microbiome respond to these oxidants, especially commensal and other health-associated species. We have now mapped the stress response to both H_2_O_2_ and HOCl in the intestinal lactic acid bacterium Lactobacillus reuteri.

## Abstract (precis)

Reactive oxidants, including hydrogen peroxide and hypochlorous acid, are antimicrobial compounds produced by the immune system during inflammation. Little is known, however, about how many important types of bacteria present in the human microbiome respond to these oxidants, especially commensal and other health-associated species. We have now mapped the stress response to both H_2_O_2_ and HOCl in the intestinal lactic acid bacterium Lactobacillus reuteri.

## INTRODUCTION

Inflammatory diseases of the gut (e.g., inflammatory bowel disease [IBD], Crohn’s disease, and irritable bowel syndrome [IBS]) are a rapidly growing health concern for which few effective treatment options are available (1–3). It has become increasingly clear that the bacterial populations inhabiting the gut play a key role in causing and perpetuating gut inflammation, with an emerging consensus that blooms of facultatively anaerobic enterobacteria (e.g., Escherichia coli) take advantage of changes in the nutritional and redox environment of the inflamed gut to outcompete the obligate anaerobes associated with a healthy gut microbiota (4–8). The redox changes in the inflamed gut include not only increases in oxygen levels (6, 7) but also the production of reactive oxygen, nitrogen, and chlorine species (ROS, RNS, and RCS, respectively), which are antimicrobial oxidants that can shift the population structure of the microbiome and are major contributors to host tissue damage (9–12). Treatments that interfere with the ability of enterobacteria to thrive in the inflamed gut reduce both the changes in the microbiome and the symptoms of disease (1, 13), indicating that manipulating gut bacteria is an important element in controlling these diseases.

Probiotics are live microorganisms which, when consumed in sufficient quantities, have a measurable health benefit (14), and a variety of different probiotic bacteria have been shown to have anti-inflammatory effects in the gut (15, 16). The most commonly used probiotics are lactic acid bacteria of the genus *Lactobacillus* (17), which are able to both modulate the host immune system and outcompete enterobacterial pathogens (15) and some strains of which have been shown to improve outcomes for inflammatory bowel diseases in both humans and animal models (18–20). The effectiveness of probiotics for treating inflammation in the gut, however, may be limited by their ability to survive attack by the overactive host immune system, including the oxidative damage caused by ROS and RCS. While the general stress response physiology of lactic acid bacteria has been relatively well characterized (21), bacterial responses to oxidative stress are best understood for E. coli and related inflammation-enriched enterobacteria (22–25). This is especially true of RCS, including hypochlorous acid (HOCl) and reactive chloramines, which are extremely potent antimicrobial compounds produced by the neutrophil enzyme myeloperoxidase (22, 26–28). Relatively little is known about how health-associated probiotic and commensal bacteria sense and respond to inflammatory oxidants (21, 29–31).

Lactobacillus reuteri is a well-established model probiotic bacterium that is able to stably colonize the mammalian intestine (32, 33), where several strains have been shown to combat inflammation and enteric infections by different mechanisms, including anti-inflammatory histamine synthesis by strains ATCC PTA 6475 and ATCC PTA 5289 (34–37), modulation of immune cell functions by strains ATCC PTA 6475, 100-23, and WU (33, 38–40), and production of antimicrobial compounds (e.g., reuterin and reutericyclin) by ATCC PTA 6475 and many other strains (37, 41, 42). While the genome-wide stress responses of L. reuteri to low pH (strain ATCC 23272) (43) and bile salts (strains ATCC PTA 6475 and 23272) (44, 45) have been characterized, little is known about how members of this species respond to ROS, and nothing is known about how L. reuteri or any other lactic acid bacterium senses or responds to RCS. The L. reuteri genome encodes neither catalase nor superoxide dismutase (46). The oxidative stress repair enzyme methionine sulfoxide reductase (47) is induced by and required for gut colonization by L. reuteri strain 100-23 (48, 49), indicating that resistance to oxidative damage is important *in vivo*. A cysteine-dependent pathway contributing to H_2_O_2_ and O_2_ tolerance has been identified in strain BR11 (50) but did not appear to play a role in the ability of L. reuteri BR11 to prevent colitis in mice (51).

In this work, we have taken a transcriptomic approach to characterize genome-wide H_2_O_2_- and HOCl-dependent gene regulation in L. reuteri ATCC PTA 6475 and to identify genes involved in resistance to killing by these stressors (52), with the goal of finding genes and pathways distinct from those found in the enterobacteria. Our results show that despite not containing close homologs of any of the known RCS-specific transcription factors (22, 53–56), L. reuteri is able to mount clearly different stress responses to H_2_O_2_ and HOCl and that the presence of O_2_ has dramatic effects on both gene regulation and survival in response to these stresses. We also identified roles for several genes in surviving H_2_O_2_- and HOCl-mediated stress, including those encoding methionine sulfoxide reductase (47), polyphosphate kinase 2 (57, 58), and the lactic acid bacterium-specific small heat shock protein Lo18 (59–61), as well as a role in surviving H_2_O_2_ stress for RsiR, previously characterized as an L. reuteri-specific regulator of histamine synthesis (35).

## RESULTS AND DISCUSSION

### Growth of L. reuteri is inhibited by inflammatory oxidants.

**FIG 1:**
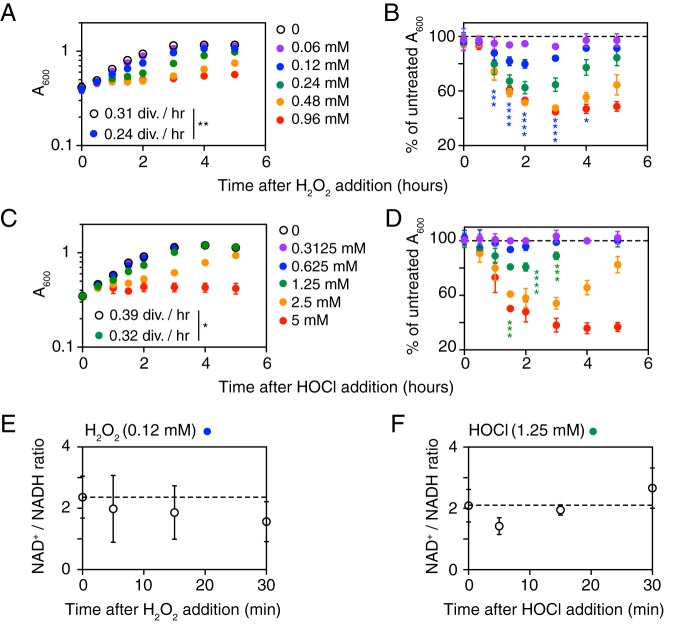
Growth of L. reuteri is inhibited by H_2_O_2_ and HOCl. L. reuteri ATCC PTA 6475 was grown anaerobically at 37°C to an *A*_600_ of 0.3 to 0.4 in MEI-C and then treated with the indicated concentrations of H_2_O_2_ (A, B, and E) or HOCl (C, D, and F) (*n* = 3, means ± SDs). *A*_600_ (A, B, C, and D) or NAD^+^/NADH ratios (E and F) were measured at the indicated times. Student’s *t* test was used to test differences between growth rates in the first 2 h after stress treatment (divisions per hour) (*, *P* < 0.05; **, *P* < 0.01). Two-way analysis of variance (ANOVA) with Holm-Sidak’s multiple-comparison test was used to test differences between normalized *A*_600_ values for stress-treated cells and untreated cultures, and significant differences for 0.12 mM H_2_O_2_ and 1.25 mM HOCl stress are indicated (*, *P* < 0.05; ***, *P* < 0.001; ****, *P* < 0.0001). Two-way ANOVA with Holm-Sidak’s multiple-comparison test was used to test differences between NAD^+^/NADH ratios before and after stress treatments, and no significant differences were found.

To begin characterizing the response of L. reuteri to inflammatory oxidants, we treated anaerobically growing cultures with different concentrations of H_2_O_2_ (Fig. 1A) and HOCl (Fig. 1B). L. reuteri growth was more sensitive to H_2_O_2_ than HOCl, with an increase in culture density of less than 0.1 *A*_600_ unit 5 h after 0.96 mM H_2_O_2_ or 5 mM HOCl treatment. Since we were interested in characterizing gene regulation during a successful, productive response to bacteriostatic stress, we selected concentrations of 0.12 mM H_2_O_2_ and 1.25 mM HOCl for further analysis. These concentrations resulted in reductions in growth rate after stress treatment to 80% of that of untreated cells, followed by complete recovery (Fig. 1A and C). Growth was significantly inhibited by 0.12 mM H_2_O_2_ between 1 and 4 h after treatment and by 1.25 mM HOCl between 1.5 and 3 h after treatment (Fig. 1B and D). The growth rates of untreated cells differed between these experiments (0.31 division h^−1^ for the H_2_O_2_ treatment experiment and 0.39 division h^−1^ for the HOCl experiment), which we hypothesize may reflect batch-to-batch variations in the MEI-C medium (see Materials and Methods) used. These concentrations of oxidants had no significant effect on cellular NAD^+^/NADH ratios (Fig. 1E and F and Fig. S1), indicating that the bacteriostatic effects of these H_2_O_2_ and HOCl concentrations did not involve major disruptions to the redox state of the bacterial cells.

10.1128/mSystems.00453-19.2 FIG S1 NAD^+^ and NADH levels in redox-stressed L. reuteri. L. reuteri ATCC PTA 6475 was grown anaerobically at 37°C to an *A*_600_ of 0.3 to 0.4 in MEI-C and then treated with 0.12 mM H_2_O_2_ (A) or 1.25 mM HOCl (B). NAD^+^ and NADH concentrations were measured at the indicated times and normalized to CFU (*n* = 3, means ± SDs). Two-way ANOVA with Holm-Sidak’s multiple-comparison tests identified no significant (*P* < 0.05) differences in NAD^+^ or NADH concentrations at any time points. Download FIG S1, EPS file, 1.2 MB. Copyright © 2019 Basu Thakur et al. 2019 Basu Thakur et al. This content is distributed under the terms of the Creative Commons Attribution 4.0 International license.

### Transcriptomic analysis of H_2_O_2_ and HOCl response by L. reuteri.

**FIG 2:**
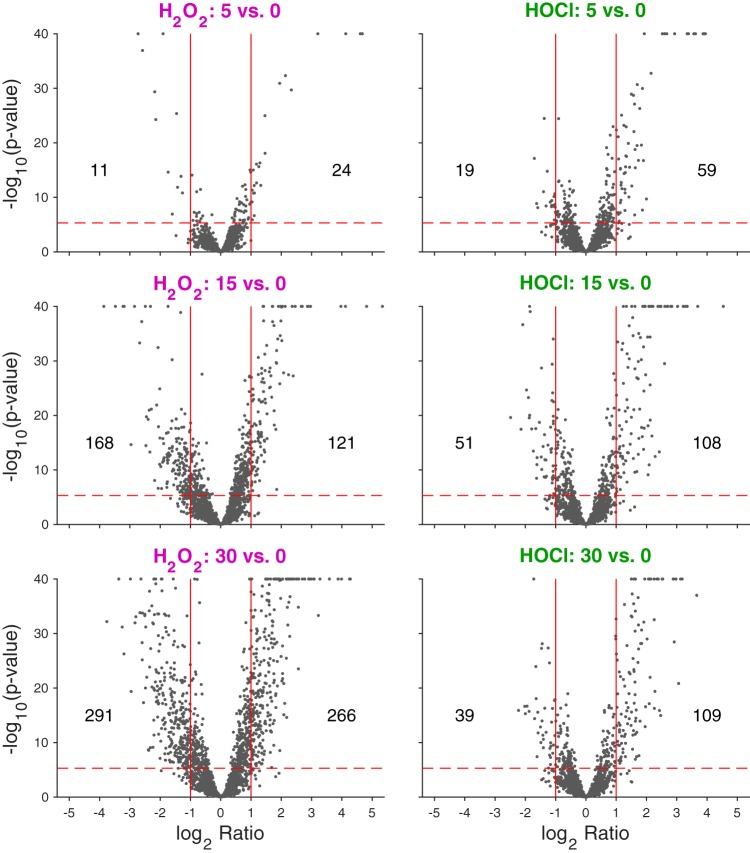
Volcano plot of log_2_ fold changes in gene expression after stress treatment. −Log_10_ *P* values are plotted against log_2_ fold changes in gene expression in anaerobically grown L. reuteri ATCC PTA 6475 treated with 0.12 mM H_2_O_2_ (left plots) or 1.25 mM HOCl (right plots) 5, 15, and 30 min after stress treatment (rows). Solid red vertical lines indicate 2-fold up and down changes relative to baseline. The horizontal red dashed line corresponds to a Bonferroni-corrected *P* value of 0.01; numbers of genes below this value (above dashed line) and with changes greater than 2-fold (up or down) are indicated on the plots. −Log_10_ *P* values >40 are set to 40 for these visualizations. See Table S2 in the supplemental material for a full list of genes whose expression changed significantly under any treatment condition.

We next treated anaerobically growing L. reuteri with 0.12 mM H_2_O_2_ or 1.25 mM HOCl and used RNA sequencing to characterize the transcriptomes of stressed cells before and 5, 15, and 30 min after stress treatment (Fig. 2 and Table S1). Up to 27% of the L. reuteri genome was up- or downregulated (>2-fold; Bonferroni-corrected *P* value [*P*_Bonf_] < 0.01; up to 48% without the 2-fold restriction) after stress treatment (Table S2), and there were clear differences in the responses to H_2_O_2_ and HOCl, consistent with previous reports that bacterial responses to these oxidants are different (22–24). As shown in Fig. 2 and Table S2, the response to H_2_O_2_ involved roughly equal numbers of up- and downregulated genes (2-fold more upregulated at 5 min, 1.4-fold more downregulated at 15 min, and 1.1-fold more downregulated at 30 min), with an increase in the number of genes with significant changes in expression over the 30-min course of stress treatment (from 35 at 5 min to 557 at 30 min, a 16-fold increase). In contrast, HOCl treatment caused 2- to 3-fold more genes to be upregulated than downregulated at all time points, and there was a smaller increase in the number of genes with significant changes in gene expression over time (from 78 at 5 min to 148 at 30 min, a 2-fold increase), consistent with the very high reaction rate of HOCl with biological molecules (22, 27, 62). The differences between the H_2_O_2_ and HOCl stress responses were also reflected in principal-component analysis of the transcriptomic data (Fig. S2A), which clearly separated the H_2_O_2_- and HOCl-treated samples. The untreated samples from the two experiments did not cluster as closely together as we expected, since these samples were ostensibly identical. To determine whether this reflected batch effects (possibly due to variations in the growth medium, as mentioned above) or inherent variation in expression levels for particular genes, we selected representative genes that had the same or significantly different levels of expression (*n* = 3 each) in the untreated samples from the two transcriptome sequencing (RNA-seq) data sets (Fig. S2B and Table S1) and used quantitative reverse transcription-PCR (qRT-PCR) to measure their expression in independently prepared unstressed L. reuteri cultures. We found that the amounts of variation in expression were similar for all six genes analyzed, with no significant differences between experiments (Fig. S2C). To minimize the effect of the differences between untreated samples from the H_2_O_2_ and HOCl RNA-seq experiments, all of the untreated samples from both experiments (*n* = 6) were considered a single group for the DeSeq2 analyses of RNA-seq data.

**FIG 3:**
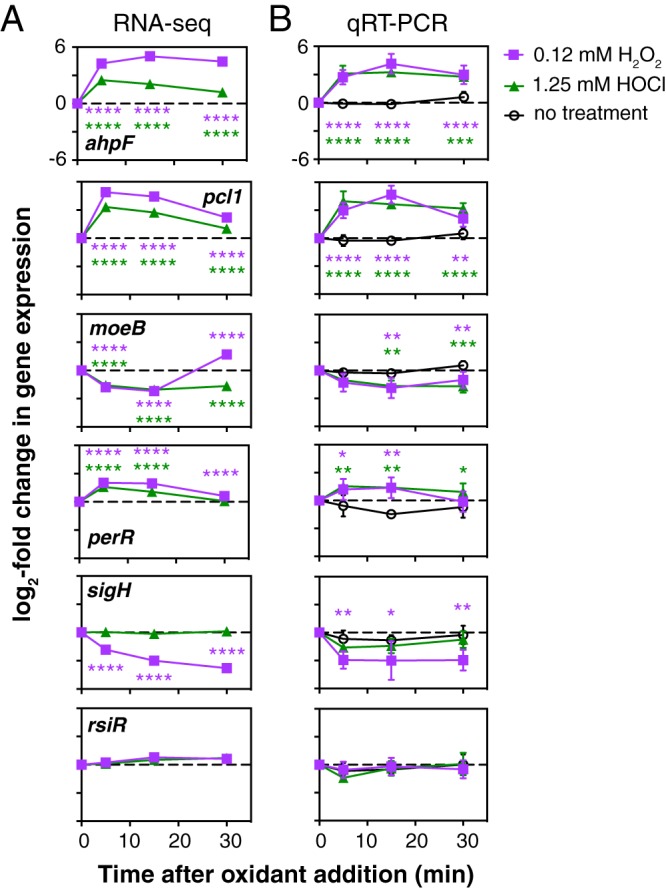
qRT-PCR validation of RNA-seq results for selected genes. (A) Log_2_ fold change in gene expression for *ahpF* (LAR_RS05795), *pcl1* (LAR_RS08080), *moeB* (LAR_RS05335), *perR* (LAR_RS06970), *sigH* (LAR_RS04695), and *rsiR* (LAR_RS05165), from RNA-seq experiments (Table S1). Bonferroni-corrected *P* values comparing each time point to expression at 0 min (untreated samples) are indicated (****, *P* < 0.0001). (B) L. reuteri ATCC PTA 6475 was grown anaerobically at 37°C to an *A*_600_ of 0.3 to 0.4 in MEI-C and then treated with the indicated concentrations of H_2_O_2_ or HOCl or not treated (*n* = 3, means ± SDs). qRT-PCR was used to measure log_2_ fold changes in expression for each gene at the indicated time points. Two-way ANOVA with Holm-Sidak’s multiple-comparison test was used to determine differences between H_2_O_2_- or HOCl-treated samples and untreated samples at each time point (*, *P* < 0.05; **, *P* < 0.01; ***, *P* < 0.001; ****, *P* < 0.0001).

10.1128/mSystems.00453-19.3 FIG S2 Differences in gene expression between untreated cells in RNA-seq analysis of H_2_O_2_- and HOCl-treated cells are probably due to batch effects, not inherently high variability in expression of those genes. (A) Principal-component analysis showing transcriptional response trajectories following treatments. The first two principal components are shown computed based on all 2,010 genes for all 24 samples of anaerobically grown L. reuteri treated with H_2_O_2_ (purple symbols) or HOCl (green symbols) at 5, 15, and 30 min after stress treatment (gray symbols). Lines connect means of replicates at each treatment and time point (*n* = 3) and baseline (*n* = 6). (B) Log_2_ normalized counts of selected genes from RNA-seq of untreated cells from H_2_O_2_ and HOCl stress experiments (Table S1) (*n* = 3, means ± SDs). Asterisks indicate corrected *P* values (ns, *P* > 0.05; *, *P* < 0.05; **, *P* < 0.01; ****, *P* < 0.0001) for the comparison of log_2_ normalized counts from the two experiments. (C) L. reuteri ATCC PTA 6475 was grown anaerobically at 37°C to an *A*_600_ of 0.3 to 0.4 in MEI-C for treatment with the indicated oxidants (see Fig. 3). Expression of the indicated genes in untreated samples from each of these experiments was measured by quantitative RT-PCR (*n* = 3, means ± SDs), with each threshold cycle (*C_T_*) normalized to that of 16S rRNA (*rsmB*) (E. S. Friedman, K. Bittinger, T. V. Esipova, et al., Proc Natl Acad Sci U S A 115:4170–4175, 2018, https://doi.org/10.1073/pnas.1718635115). Two-way ANOVA with Holm-Sidak’s multiple-comparison correction indicated no significant differences (*P* > 0.05) between Δ*C_T_* values for each experiment for any tested gene. Download FIG S2, EPS file, 1.6 MB. Copyright © 2019 Basu Thakur et al. 2019 Basu Thakur et al. This content is distributed under the terms of the Creative Commons Attribution 4.0 International license.

10.1128/mSystems.00453-19.7 TABLE S1 Results of RNA sequencing of H_2_O_2_- and HOCl-treated anaerobically grown cultures of L. reuteri. Columns A to I contain identifying information and annotation for each of the 2,010 genes in the L. reuteri ATCC PTA 6475 genome. Columns J to AG contain raw counts from RNA sequencing for each gene under baseline (0 min, untreated), HOCl (5, 15, and 30 min), or H_2_O_2_ (5, 15, and 30 min) stress. Columns AH to BE contain log_2_ transformed normalized counts for each condition. Columns BF to CC contain rlog normalized counts for each condition. Columns CD to CI contain average log_2_ fold changes in gene expression relative to baseline at each time point (5, 15, and 30 min) for HOCl- and H_2_O_2_-stressed samples. Columns CJ to CO and CP to CU contain *P* values and Bonferroni-corrected *P* values, respectively, for the significance of the log_2_ fold changes in gene expression. Download Table S1, XLSX file, 2.5 MB. Copyright © 2019 Basu Thakur et al. 2019 Basu Thakur et al. This content is distributed under the terms of the Creative Commons Attribution 4.0 International license.

10.1128/mSystems.00453-19.8 TABLE S2 Genes whose log_2_ ratio was >1 or <−1 and adjusted *P* values were <0.01 for comparisons of any timepoint versus 0 min for both H_2_O_2_ and HOCl. Columns A and C indicate the time point under consideration, columns A and B indicate the stressor, column D indicates the direction of regulation (either up or down relative to expression at 0 min), and columns E and F indicate the gene. For detailed gene expression and log_2_ fold change values for each gene, see Table S1. Download Table S2, DOCX file, 0.1 MB. Copyright © 2019 Basu Thakur et al. 2019 Basu Thakur et al. This content is distributed under the terms of the Creative Commons Attribution 4.0 International license.

We next used qRT-PCR to validate our RNA-seq results (Fig. 3). We examined two genes strongly activated by both H_2_O_2_ and HOCl (*ahpF* and *pcl1*, encoding alkylhydroperoxidase and a predicted iron transporter, respectively) and a gene repressed by both oxidants (*moeB*, encoding a subunit of molybdopterin synthase [63]) (Table S1) and confirmed the expected expression patterns. RT-PCR of the redox-responsive *perR* and *sigH* genes, encoding predicted transcription factors, also recapitulated the expression patterns seen in RNA-seq. Finally, we examined expression of *rsiR*, a known regulator of anti-inflammatory mechanisms in L. reuteri ATCC PTA 6475 (34, 35) that has also been reported to regulate some redox response genes (35), and we confirmed that its expression is not regulated by either H_2_O_2_ or HOCl.

**FIG 4:**
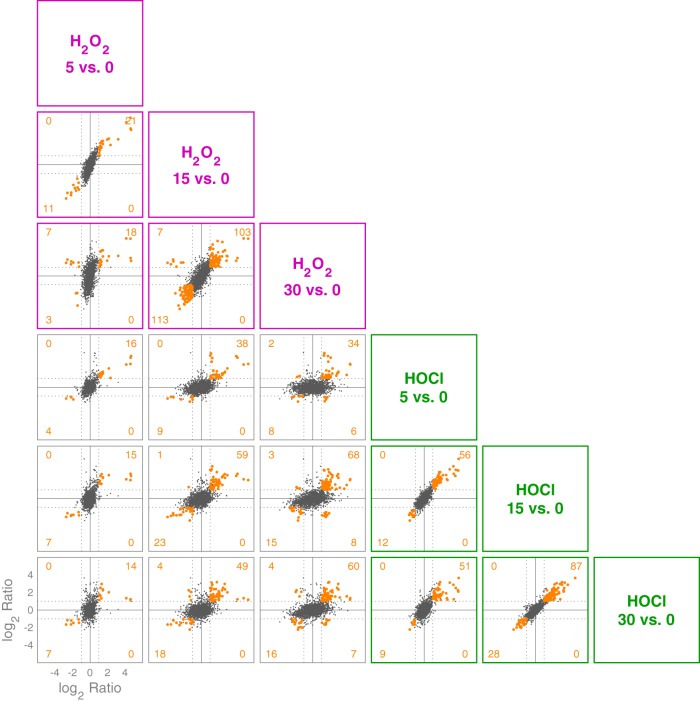
Trellis plot comparing log_2_ ratios for pairs of pairwise comparisons. Each individual plot below the diagonal compares the log_2_ ratio of each gene of the treatment time point versus baseline pairwise comparison indicated by the column versus that indicated by the row. Those plots outlined in purple or green show fold changes of genes at different time points for either H_2_O_2_ or HOCl treatment, respectively. The remaining plots compare fold changes between the treatments. Orange data points represent those genes where the Bonferroni-corrected *P* value is <0.01 and the absolute value of the log_2_ ratio is >1 for both pairwise comparisons; numbers of these genes are indicated in each quadrant of each plot. See Table S2 for individual genes in each expression category.

**FIG 5:**
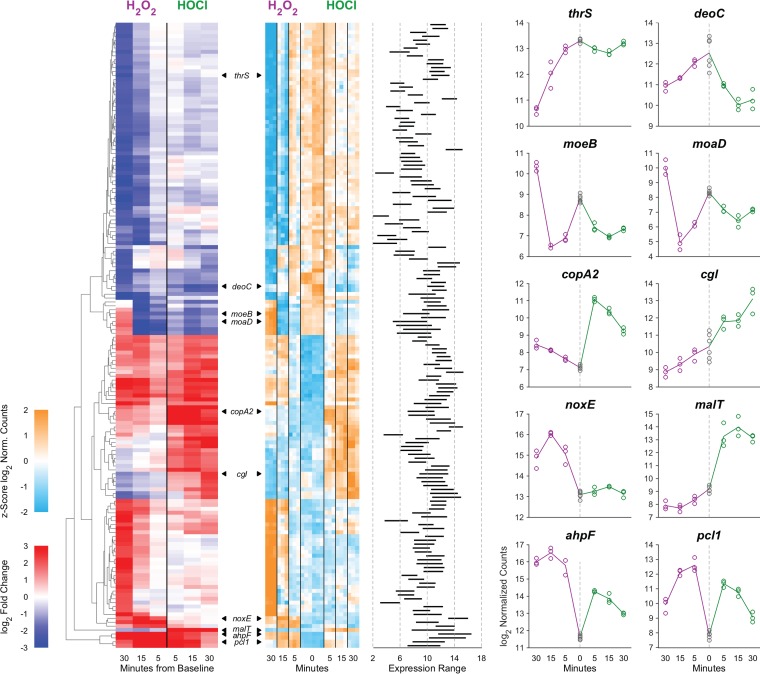
Cluster analysis of gene expression levels in anaerobically grown L. reuteri ATCC PTA 6475 treated with 0.12 mM H_2_O_2_ (purple) or 1.25 mM HOCl (green) 0, 5, 15, and 30 min after stress treatment (times plotted outward from the center for each panel). A total of 160 genes are shown that had a 4-fold change in expression and Bonferroni-corrected *P* value of <0.01 for at least one of the six treatment time point comparisons against baseline. The red-blue heat map at the left shows the fold changes (color) of these 160 genes (rows) for each of the six pairwise comparisons (columns). Clustering was performed to illustrate the diversity of response profiles rather than to establish a number of “canonical” patterns. Genes (rows) were hierarchically clustered based on Euclidean distance and average linkage. The second, teal-orange heat map shows the per-gene z-score (the number of SDs away from the mean of expression in the reference) of log_2_ normalized data of genes (in the same order) for each replicate of each treatment/time point and baseline (columns). The next plot shows the range of the log_2_ normalized expression data (of averaged replicates) for each of the genes. Expression data for 10 representative genes are plotted to the right (symbols, individual replicates; lines connect means of replicates). Called-out genes are *thrS* (LAR_RS09850), *deoC* (LAR_RS00565), *moeB* (LAR_RS05335), *moaD* (LAR_RS05395), *copA2* (LAR_RS02280), *cgl* (LAR_RS01550), *noxE* (LAR_RS00345), *malT* (LAR_RS00275), *ahpF* (LAR_RS05795), and *pcl1* (LAR_RS08080).

To further characterize the differences and overlaps between the H_2_O_2_ and HOCl stress responses of L. reuteri, we plotted changes in gene expression under each tested condition against each other condition (Fig. 4 and Table S2). There were 78 genes that were upregulated (at at least one time point) by both stressors (287 for H_2_O_2_ and 133 for HOCl) and 40 genes that were downregulated (at at least one time point) by both stressors (346 for H_2_O_2_ and 69 for HOCl). As shown in Fig. 4, there were 12 genes (11 unique) that had reciprocal responses at the same time point (see diagonals of gray-outlined plots in Fig. 4). In general, there were 16 unique genes that were up in at least one time point in H_2_O_2_ and down in at least one time point in HOCl (or vice versa). Furthermore, there were 73, 282, and 531 genes at 5, 15 and 30 min, respectively, that were significantly changed (*P*_Bonf_ < 0.01, 2-fold up or down) with one stressor but not the other, all of which suggests that, despite lacking homologs of known HOCl-sensing transcription factors (22, 53–55, 64, 65), L. reuteri has a sophisticated ability to distinguish between H_2_O_2_ and HOCl and differentially control transcription. This is consistent with results with E. coli and Bacillus subtilis, in which H_2_O_2_ and HOCl stress responses partially overlap but have substantial oxidant-specific components (22, 27, 56, 62, 66). Cluster analysis of genome-wide expression patterns (Fig. 5) reinforced this result, and we were able to identify genes whose expression was controlled in very similar ways by the different oxidants as well as groups of genes with very different expression patterns in response to H_2_O_2_ and HOCl, including, for example, *cgl* and *cyuABC*, which encode a previously characterized cysteine-dependent redox stress response pathway (50). Examples of genes with distinct patterns of regulation are illustrated in the rightmost columns of Fig. 5.

L. reuteri’s response to H_2_O_2_ was generally consistent with what has been previously observed with other catalase-negative Gram-positive bacteria (21, 23, 24, 31, 65, 67, 68). Highly upregulated genes included genes encoding alkylhydroperoxidase (*ahpCF*) (69), NADH oxidase (*noxE*) (70), and methionine sulfoxide reductase (*msrB*) (47), DNA repair genes (*uvrABD*, *xthA*, and *umuC*) (71), and genes for predicted metal transporters (*pcl1* and *pcl2*) and the peroxide-sensing transcription factor PerR (65). The response to HOCl was also, in broad strokes, similar to that of previously characterized bacteria (22), in that upregulated genes included those involved in proteostasis (*groSL*, *clpE*, and *hsp20/lo18*), metal stress (*pcl1*, *pcl2*, and *copAR*), thioredoxins (*trxABD*), and cysteine and methionine synthesis (*cysK* and *metE*). Genes upregulated by both stressors included not only *msrB*, *ahpCF*, *perR*, and the genes for predicted iron transporters (*pcl1* and *pcl2*) but also genes for a variety of predicted sugar and amino acid transporters and metabolic enzymes (*oxc*, encoding oxalyl-CoA decarboxylase [72], for example). These may represent responses to changes in the nutritional environment L. reuteri might encounter in the inflamed gut (6, 8).

### Redox-regulated transcription factors in L. reuteri.

**FIG 6:**
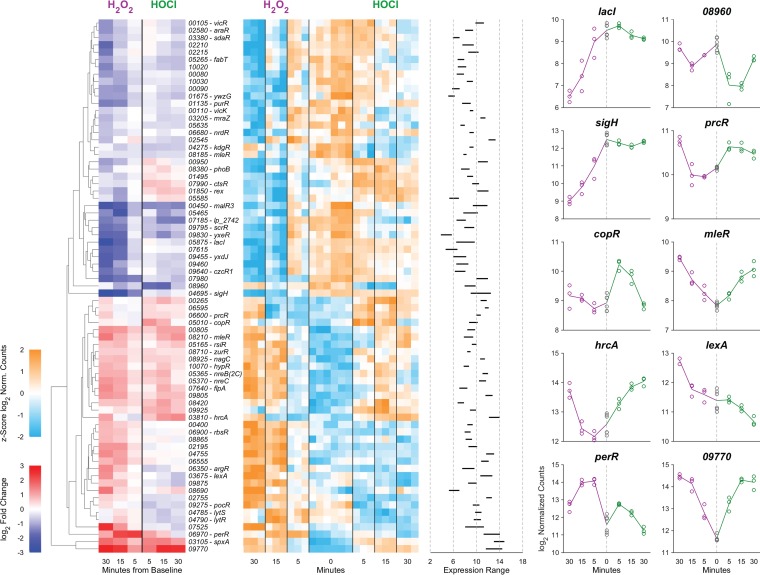
Cluster analysis of gene expression for transcription factors in anaerobically grown L. reuteri ATCC PTA 6475 treated with 0.12 mM H_2_O_2_ (purple) or 1.25 mM HOCl (green) 0, 5, 15, and 30 min after stress treatment. Responses of 73 transcription factors whose expression changed significantly from 0 min under at least one stress treatment condition are shown. The format of this figure is identical to that of Fig. 5. Called-out genes are *lacI* (LAR_RS05875), LAR_RS08960, *sigH* (LAR_RS04695), *prcR* (LAR_RS06600), *copR* (LAR_RS05010), *mleR* (LAR_RS08185), *hrcA* (LAR_RS03810), *lexA* (LAR_RS03675), *perR* (LAR_RS06970), and LAR_RS09770.

While many bacterial transcription factors that respond to H_2_O_2_ and/or HOCl have been described, L. reuteri encodes only a few homologs of known H_2_O_2_-detecting transcription factors (e.g., PerR and VicK [24, 68]) and no close homologs of any of the known HOCl-detecting transcription factors (22, 53–55, 64, 65). This suggested that among the 102 predicted transcription factors encoded by the L. reuteri genome, there are likely to be novel redox-sensing regulators. To begin to assess this possibility, we performed cluster analysis of the expression of genes encoding transcription factors that showed a significant change with either stressor at any time point (*n* = 73) under both stress conditions (Fig. 6), reasoning that many bacterial transcription factors are autoregulated and that changes in expression of transcription factors are useful signposts for identifying regulatory stress response networks (53, 66, 73). We found genes encoding predicted transcription factors whose expression was activated by both H_2_O_2_ and HOCl (e.g., *perR*, *spxA*, and LAR_RS09770), repressed by both H_2_O_2_ and HOCl (e.g., *kdgR* and *fabT*), activated only by H_2_O_2_ (e.g., *lexA* and LAR_RS07525), activated only by HOCl (e.g., *ctsR* and *copR*), repressed only by H_2_O_2_ (e.g., *sigH* and *rex*), and repressed only by HOCl (e.g., *malR3* and LAR_RS02755), indicating the presence of a complex regulatory response to both oxidants. Some of these regulators have known functions, which give useful insights into the *in vivo* effects of H_2_O_2_ and HOCl on L. reuteri. For example, only HOCl activated expression of *ctsR*, a conserved regulator of protein quality control in Gram-positive bacteria (74, 75), consistent with the known ability of HOCl to unfold and aggregate proteins (76, 77) and the activation of the heat shock response in many species of HOCl-stressed bacteria (22). On the other hand, only H_2_O_2_ activated expression of the DNA-damage responsive *lexA* regulator (71), consistent with the known ability of H_2_O_2_ to damage DNA (23) and suggesting that HOCl does not cause DNA damage at the concentration used in this experiment. However, most of the transcription factors in L. reuteri have no known function, and the expression patterns of many of these genes were affected by the redox stress treatments. For example, the only alternative sigma factor (78) encoded in the L. reuteri genome (*sigH*) was downregulated strongly by H_2_O_2_ but unaffected by HOCl. We do not currently know what genes these uncharacterized regulators regulate, what role(s) they may play in surviving redox stress, or what transcription factor(s) is responsible for HOCl-specific regulation in L. reuteri.

### Oxygen affects H_2_O_2_- and HOCl-dependent gene expression in L. reuteri.

**FIG 7:**
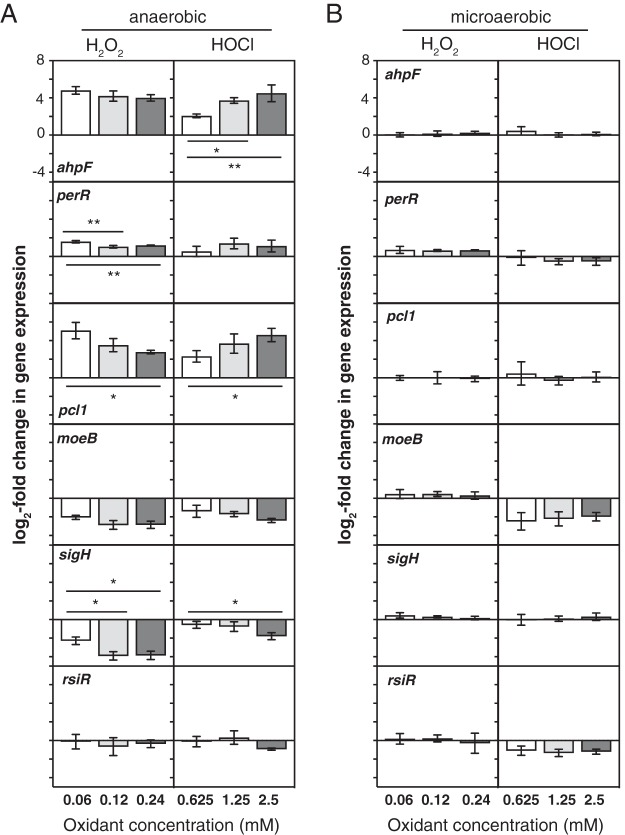
Dose-responsive control of gene expression by oxidative stress. L. reuteri ATCC PTA 6475 was grown anaerobically or microaerobically at 37°C to an *A*_600_ of 0.3 to 0.4 in MEI-C and then treated for 15 min with the indicated concentrations of H_2_O_2_ or HOCl. Change in expression of the indicated genes relative to untreated control cells was measured by quantitative RT-PCR (*n* = 3, means ± SDs). Asterisks indicate significant differences in expression at different oxidant concentrations under a given growth condition (two-way ANOVA with Holm-Sidak’s multiple-comparison correction) as follows: *, *P* < 0.05, and **, *P* < 0.01.

We used quantitative RT-PCR to measure the dose responsiveness of changes in expression of selected genes in anaerobically grown L. reuteri 15 min after treatment with concentrations of H_2_O_2_ and HOCl at, above, and below the nonbactericidal concentrations used in previous experiments (Fig. 7A). Interestingly, the genes differed in their dose-response patterns, with *moeB* equally repressed at all H_2_O_2_ and HOCl concentrations, *ahpF* equally upregulated by all three H_2_O_2_ concentrations but activated more strongly by increasing doses of HOCl, and *pcl1* upregulated more strongly at lower doses of H_2_O_2_ and at higher doses of HOCl. Expression of *sigH* was repressed at higher HOCl concentrations (2.5 mM), indicating that its control is not strictly H_2_O_2_ specific.

While the intestine is primarily an anaerobic environment (5), exact oxygen concentrations are difficult to measure *in vivo* and may vary depending on anatomical position or specific microenvironments in the intestine (79). Recent evidence suggests that inflammation, antibiotic treatment, and infection with enteric pathogens may increase the amount of oxygen available to microbes in the gut (6, 7). We therefore wanted to assess how much of an effect oxygen has on expression of redox-regulated genes in L. reuteri. We repeated our RT-PCR experiment with microaerobic cultures, which were prepared aerobically and grown in full screw-cap tubes without shaking, low-oxygen conditions under which L. reuteri, like other lactic acid bacteria (70, 80), can remove oxygen from liquid media and grow to the same density as under anaerobic conditions (Fig. S3). The results of this experiment (Fig. 7B) revealed that the presence of even the low levels of oxygen expected in these cultures had large effects on redox-responsive gene expression. In contrast to what we observed anaerobically, expression of *ahpF*, *pcl1*, *moeB*, and *sigH* was unaffected by H_2_O_2_ under these conditions, and activation of *perR* was reduced. HOCl activation of *ahpF*, *pcl1*, and *perR* expression was eliminated in the presence of oxygen, and expression of both *moeB* and *rsiR* expression was HOCl repressed. These results showed that oxygen can dramatically affect how bacteria regulate gene expression in response to inflammatory oxidants and that studies of redox responses in the presence of even small amounts of oxygen may not necessarily reflect how bacteria respond in anaerobic environments and vice versa.

10.1128/mSystems.00453-19.4 FIG S3 L. reuteri removes oxygen from statically grown microaerobic cultures. (A) The L. reuteri ATCC PTA 6475 wild type was grown for 18 h at 37°C in MEI-C either anaerobically, microaerobically (in full screw-cap tubes without shaking), or aerobically (5 ml of medium in a test tube with shaking at 200 rpm) and then diluted for measurement of culture density (*A*_600_). Asterisks indicate significant differences in culture density (two-way ANOVA with Holm-Sidak’s multiple-comparison correction as follows: *, *P* < 0.05. (B) In the left-hand tube, L. reuteri was grown microaerobically as described for panel A, with 2 mg liter^−1^ of the oxygen indicator dye methylene blue included in the MEI-C medium. The right-hand tube was not inoculated with L. reuteri. Download FIG S3, EPS file, 2.6 MB. Copyright © 2019 Basu Thakur et al. 2019 Basu Thakur et al. This content is distributed under the terms of the Creative Commons Attribution 4.0 International license.

### Identifying genes important for surviving oxidative stress in L. reuteri.

Finally, we wanted to use the gene expression data generated as described above to begin identifying genes involved in protecting L. reuteri against the toxicity of H_2_O_2_ and HOCl, based on the simple hypothesis that genes strongly upregulated by a certain stress may be involved in protecting the cell against that stress (81). We were particularly interested in identifying genes encoding factors that protect L. reuteri against HOCl, since much less is known about HOCl defense in bacteria in general (22), and no previous studies have examined how lactic acid bacteria survive reactive chlorine stress. We therefore identified bactericidal doses of H_2_O_2_ and HOCl for L. reuteri (Fig. S4A) and found that 1.5 mM H_2_O_2_ was sufficient to cause a 99.9% loss in viable cells of L. reuteri over the course of an hour both anaerobically and microaerobically. Subsequent titration of bactericidal HOCl concentrations (Fig. S4B) showed that doses resulting in a rate of viability loss comparable to that seen with 1.5 mM H_2_O_2_ in the first 40 min after treatment also resulted in recovery of viable cells after 60 min. We therefore used bactericidal concentrations of HOCl that resulted in a 99.999% loss in viable cells with no recovery during the 1-h course of the experiment (7.5 mM anaerobically and 2.5 mM microaerobically).

10.1128/mSystems.00453-19.5 FIG S4 Bactericidal oxidative stress treatment of L. reuteri. The L. reuteri ATCC PTA 6475 wild type was grown at 37°C to an *A*_600_ of 0.3 to 0.4 in MEI-C anaerobically (A) or microaerobically (B) and then treated with the indicated concentrations of H_2_O_2_ or HOCl. At the indicated times, oxidants were quenched (3 mM sodium pyruvate for H_2_O_2_ and 25 mM sodium thiosulfate for HOCl), and CFU were determined by plating serial dilutions on MRS agar and counting colonies formed after 24 to 48 h at 37°C anaerobically (*n* = 3 for microaerobic 1.25 mM HOCl and *n* = 15 for all other treatments, means ± SDs). Download FIG S4, EPS file, 1.2 MB. Copyright © 2019 Basu Thakur et al. 2019 Basu Thakur et al. This content is distributed under the terms of the Creative Commons Attribution 4.0 International license.

**FIG 8:**
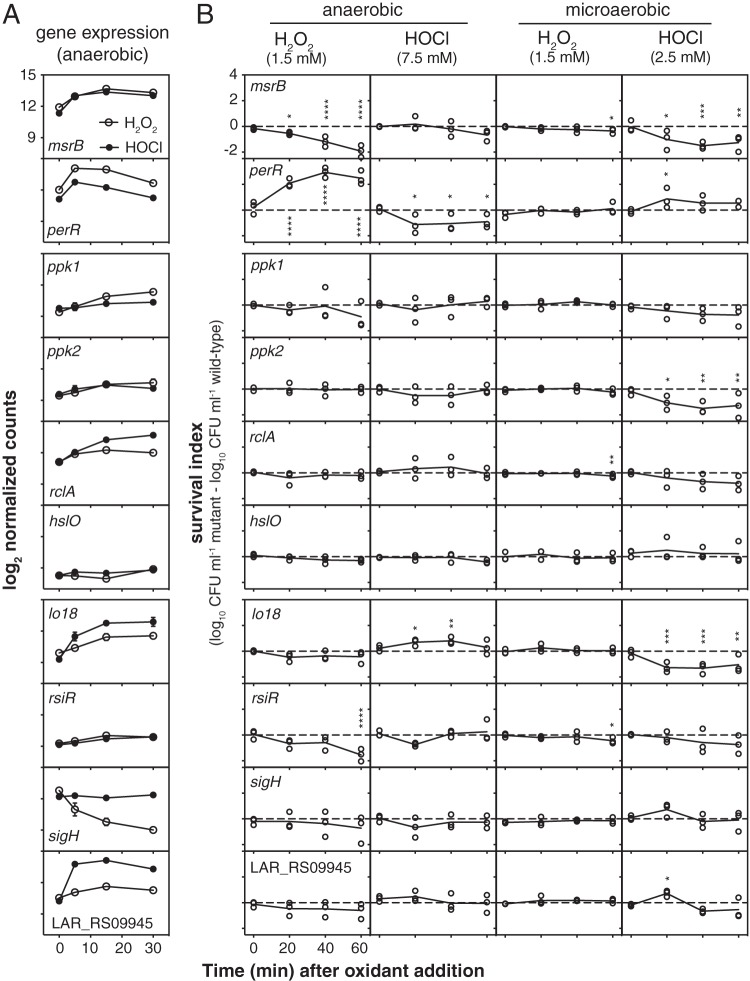
Genes regulated by inflammatory oxidants affect survival of bactericidal oxidative stress in L. reuteri. Shown are log_2_ normalized counts from RNA sequencing (A) and survival indices of anaerobically or microaerobically grown null mutants in *msrB* and *perR* (expected to be involved in H_2_O_2_ stress response), *ppk1*, *ppk2*, *rclA*, and *hslO* (expected to be involved in HOCl response), and *lo18*, *rsiR*, *sigH*, and LAR_RS09945 (found in L. reuteri but not in enterobacteria) at the indicated time points after addition of 1.5 mM H_2_O_2_ or 7.5 or 2.5 mM HOCl (B). Asterisks indicate survival indices significantly different from zero at the indicated time point (two-way repeated measures ANOVA with Holm-Sidak’s multiple-comparison test) as follows: *, *P* < 0.05; **, *P* < 0.01; ***, *P* < 0.001; and ****, *P* < 0.0001.

We constructed several strains containing null mutations of genes that we predicted to be involved in defense against either H_2_O_2_ or HOCl, based on known bacterial redox stress response mechanisms (22, 23, 53, 82) and on our transcriptomic data. We obtained mutants lacking *msrB* and *perR*, which we expected to be involved in H_2_O_2_ response, as well as four mutants lacking genes we expected to be involved in HOCl response: *ppk1* and *ppk2*, encoding two different kinases able to produce inorganic polyphosphate (polyP), which protects against HOCl-mediated protein damage in E. coli (57, 76, 83); *rclA*, encoding a conserved flavoprotein known to protect E. coli against HOCl (53); and *hslO*, encoding the widely conserved HOCl-activated chaperone Hsp33 (77). Finally, we knocked out four genes found in L. reuteri but not in enterobacteria with either interesting redox-responsive expression patterns or, in the case of *rsiR*, a known role in probiotic action: *sigH*, *rsiR*, *lo18* (*hsp20*), which encodes a small heat shock protein found only in lactic acid bacteria (59, 60) and whose expression was more strongly activated by HOCl than by H_2_O_2_, and LAR_RS09945, encoding a predicted oxidoreductase that was very strongly upregulated by HOCl but not by H_2_O_2_. The ability of each of these strains to survive bactericidal oxidative stress was measured by comparison to the viability of the wild-type strain under the same conditions (Fig. 8).

Anaerobically, the *msrB* mutant was extremely sensitive to H_2_O_2_ treatment, as expected (23, 47), and the *perR* mutant, which is expected to have constitutively high expression of peroxide defense genes (84), was significantly protected. A mutant lacking *rsiR* was significantly more sensitive to H_2_O_2_, suggesting that despite the fact that its expression is not controlled by this oxidant (Fig. 3), it is important for surviving H_2_O_2_ treatment (35). Surprisingly, only the *perR* mutant was significantly more sensitive than the wild type to HOCl under anaerobic conditions. However, knocking out *lo18* had a significant and unexpected protective effect. This was particularly surprising since *lo18* expression was strongly upregulated in response to HOCl. Under microaerobic conditions, the results of survival assays were considerably different. There were only minor differences in survival of a bactericidal dose of H_2_O_2_ in microaerobic cultures for any of the mutants, with *msrB*, *rsiR*, and *rclA* mutants showing very small but statistically significant defects in survival at the 1-h time point. In contrast, there were more significant differences in survival of HOCl stress under microaerobic conditions. The *msrB*, *perR*, *lo18*, and *ppk2* mutants had significant defects in HOCl stress survival under these conditions. The *perR* and LAR_RS09945 mutants were significantly protected at the 20-min time point, but this effect was lost at later time points. There was no difference in HOCl survival between the wild type and *sigH* or *hslO* mutants. The ability of each mutant to grow in media containing nonbactericidal concentrations of H_2_O_2_ and HOCl (Fig. S5) was more variable and the differences between mutants and the wild type of smaller magnitude, but *perR*, *hslO*, *lo18*, *rsiR*, and LAR_RS09945 mutants had significant, >2-h lags in growth relative to that of the wild type after inoculation into anaerobic media containing either H_2_O_2_ or HOCl and a *perR* mutant had a significant growth advantage in microaerobic media containing HOCl 7 to 12 h after inoculation. Overall, our results show that different redox stress treatment methods can give different results, further emphasize that oxygen concentration has dramatic effects on oxidative stress survival, and indicate that it will be important to quantify what oxygen levels gut bacteria are exposed to in inflamed and noninflamed gut environments (5–7) to understand what genes are likely to play roles in ROS and RCS resistance *in vivo*.

10.1128/mSystems.00453-19.6 FIG S5 Genes regulated by inflammatory oxidants affect resistance to inhibitory oxidative stress in L. reuteri. (A) Log_2_ normalized counts from RNA sequencing and (B) percent of wild-type *A*_600_ of anaerobically or microaerobically grown cultures with null mutations in *msrB* and *perR* (expected to be involved in H_2_O_2_ stress response), *ppk1*, *ppk2*, *rclA*, and *hslO* (expected to be involved in HOCl response), and *lo18*, *rsiR*, *sigH*, and LAR_RS09945 (found in L. reuteri but not in enterobacteria) at the indicated time points after inoculation into MEI-C containing 0.0625 mM H_2_O_2_ or 1.25 mM HOCl (*n* = 3, means ± SDs). Red symbols indicate percent of wild-type *A*_600_ significantly different from 100% at the indicated time point (two-way repeated-measures ANOVA with Holm-Sidak’s multiple-comparison test, *P* < 0.05). Download FIG S5, EPS file, 2.2 MB. Copyright © 2019 Basu Thakur et al. 2019 Basu Thakur et al. This content is distributed under the terms of the Creative Commons Attribution 4.0 International license.

Screening mutants lacking HOCl-induced genes has successfully identified HOCl resistance factors in other bacterial species (53, 54, 56, 66), but this strategy had limited success in L. reuteri. Neither *sigH* nor LAR_RS09945 mutations, for example, had any effect on resistance to the stresses which regulated their expression. In future work, a genome-wide mutant screening approach (e.g., transposon sequencing) (52) may be valuable for identifying additional genes required for H_2_O_2_ and HOCl stress survival, and complementation and overexpression analysis will be necessary to confirm that the observed phenotypes are specific to the constructed mutations in each gene. Nevertheless, our targeted mutagenesis approach did allow us to identify several important players in oxidative stress resistance. Clearly, methionine sulfoxide reductase is a major contributor to the ability of L. reuteri to resist oxidative stress both anaerobically and microaerobically, consistent with its enzymatic activity (47) and known role in colonization (48, 49). While PerR is relatively unimportant microaerobically, anaerobically it plays a key role in regulating H_2_O_2_ resistance, as expected (65), although for unknown reasons it appears that the constitutive overexpression of H_2_O_2_ resistance genes expected in a *perR* mutant is detrimental in the presence of HOCl.

### L. reuteri-specific defenses against H_2_O_2_ and HOCl stress.

The H_2_O_2_ sensitivity of the *rsiR* mutant was somewhat surprising, since this L. reuteri-specific gene has largely been characterized for its role in regulating the expression of the histamine-producing histidine decarboxylase locus of L. reuteri, where *rsiR* is essential for histamine-dependent anti-inflammatory phenotypes (34, 35). However, RsiR is a global regulator, activating and repressing transcription of 195 and 143 genes, respectively, many of which are involved in redox homeostasis (including *ahpC*, *perR*, and genes involved in cysteine and methionine synthesis) (35). It is currently unclear what signal(s) RsiR responds to, which RsiR-regulated genes contribute to H_2_O_2_ sensitivity, or what role H_2_O_2_ resistance plays in RsiR-dependent anti-inflammatory effects *in vivo*, and these are exciting issues for future research exploring the connections between inflammatory oxidants and anti-inflammatory probiotic mechanisms.

The small heat shock protein Hsp33 and the flavoprotein RclA are RCS-specific defense factors in E. coli (53, 77), so we were also surprised to find that mutations of these genes had no apparent effect on HOCl resistance in L. reuteri, despite the fact that *rclA* expression was induced more strongly by HOCl treatment than by H_2_O_2_ (Fig. 8A and Table S1). This could be due to the redundant nature of RCS resistance mechanisms (22) or could reflect fundamental differences in RCS response between L. reuteri and E. coli. Supporting the second hypothesis is the fact that mutations in *lo18* and *ppk2*, genes not found in E. coli, had very strong effects on HOCl resistance. Lo18 is a chaperone found only in a subset of *Lactobacillus* and *Oenococcus* species that stabilizes proteins and membranes under heat and ethanol stress conditions (59, 60). While this could easily explain how Lo18 protects L. reuteri against the protein-unfolding activity of HOCl, as we saw under microaerobic conditions, it is much less intuitive why the loss of Lo18 protected L. reuteri against HOCl anaerobically, and more work will be needed to understand the mechanism underlying this effect. PolyP plays a role in stress resistance and probiotic phenotypes in several different *Lactobacillus* species (85–90). In E. coli, the polyP kinase PPK (homologous to L. reuteri PPK1) is required for HOCl resistance (76), but deletion of *ppk1* had only a modest, nonstatistically significant effect on HOCl resistance in L. reuteri. In contrast, deletion of *ppk2*, which encodes an unrelated polyP kinase (PPK2) whose primary physiological role is generally thought to be in generating NTPs from NDPs or NMPs and polyP (57, 58), led to a highly significant defect in HOCl resistance, albeit only in the presence of oxygen. Whether polyP production in response to HOCl stress is driven by PPK1 or PPK2 in L. reuteri remains to be determined, as does the relative importance of PPK2’s polyP- and NTP-synthesizing activities. PPK2 is not present in enterobacteria but is found in many species of commensal bacteria (including lactobacilli, *Bacteroidetes*, and *Clostridiacea*) (58, 91, 92).

Our results clearly demonstrate that HOCl resistance in L. reuteri depends on factors different than in E. coli or B. subtilis. These differences may represent possible targets for differentially sensitizing gut bacteria to oxidative stress. Interestingly, the frontline IBD drug mesalamine has recently been shown to be an inhibitor of PPK1 (93), and it is tempting to speculate that mesalamine may therefore have a larger impact on the ability of enterobacteria to survive in the inflamed gut than on PPK2-encoding commensals, although more data will be needed to test this hypothesis.

### Conclusions.

Manipulating the microbiome is likely to be a key element in future treatments for inflammatory diseases of the gut. Development of such treatments will require a sophisticated understanding of how gut bacteria respond to changes in their environment. The differences we have now begun to uncover in oxidative stress response between anti-inflammatory, health-associated bacteria and proinflammatory, disease-associated species may present opportunities for new therapies. We hope that our results will ultimately make it possible to sensitize enterobacteria to inflammatory oxidants while simultaneously protecting the healthy gut community.

## MATERIALS AND METHODS

### Bacterial strains and growth conditions.

All strains and plasmids used in this study are listed in Table 1. All L. reuteri strains were derivatives of strain ATCC PTA 6475 (Biogaia) (94). Strain 6475*rsiR*-Stop (35) was a gift from James Versalovic (Baylor College of Medicine), and plasmid pJP042 (*recT*^+^ *erm*^+^) (94) was a gift from Jan-Peter van Pijkeren (University of Wisconsin—Madison). L. reuteri was grown at 37°C in MEI broth (86) without added cysteine (MEI-C) or on solid De Man, Rogosa, and Sharpe (MRS) agar (Difco). Anaerobic cultures were incubated in an anaerobic chamber (Coy Laboratory Products) in an atmosphere of 90% nitrogen, 5% CO_2_, and 5% H_2_ or in Hungate tubes prepared, inoculated, and sealed in that chamber. Liquid media were made anaerobic before use by equilibration for at least 24 h in the anaerobic chamber. MRS plates for CFU plate counts were incubated in sealed containers made anaerobic using GasPak EZ sachets (Becton, Dickinson). Microaerobic cultures were incubated aerobically without shaking in 16- by 125-mm screw-cap test tubes containing 15 ml of MEI-C. Methylene blue (2 mg liter^−1^) was added when indicated (95). Aerobic cultures (5 ml in a 16-mm diameter test tube) were incubated with shaking (200 rpm). Details of H_2_O_2_ and HOCl stress treatments, transcript quantification, and phenotype analysis are described in the supplemental material.

**TABLE 1:** Strains and plasmids used in this study^*a*^

Strain or plasmid	Relevant genotype	Source and/or reference
L. reuteri strains		
ATCC PTA 6475	Wild type; human breast milk isolate	Biogaia (94)
6475rsiR-Stop	*rsiR* (LAR_RS05165)^G6C, G7C, T10A, A11T, C12G, A13T^	35
MJG0562	*ppk1* (LAR_RS01770)^G94T, A95C, G96C, G97T, C98A^	
MJG0569	*ppk2* (LAR_RS00075)^A52C, C53T, G54T, G55T, C56A^	
MJG0570	*rclA* (LAR_RS00915)^C178A, A179G, T180C, G181T, G182A^	
MJG0977	*msrB* (LAR_RS00975)^G58T, T59C, T60C, A61T, C62A^	
MJG0979	*hslO* (LAR_RS01385)^G106A, A107T, T108G, A109T, C110A^	
MJG1017	*lo18* (LAR_RS07000)^T43A, T44C, G45T, A46T, T47A^	
MJG1056	LAR_RS09945^C103A, C104T, A105G, G106T, A107G^	
MJG1278	*sigH* (LAR_RS04695)^G101A, G102A, C103T, C104A, G105A^	
MJG1573	*perR* (LAR_RS06970)^G10T, C11T, A12C, G13T, A14G^	
Plasmid pJP042	*recT*^+^ *erm*^+^	94

a Unless otherwise indicated, all strains were generated in the course of this work.

### Molecular methods.

Oligonucleotide-directed recombineering was used to construct null mutations in the chromosome of L. reuteri using the pJP042-encoded RecT recombinase as previously described (94). Null mutations were designed to incorporate in-frame stop codons near the 5′ end of each gene. Mutagenic primers used are listed in Table S3. Primers used for quantitative RT-PCR were designed with Primer Quest (Integrated DNA Technologies; parameter set “qPCR 2 primers intercalating dyes” for qRT-PCR primer design) and are listed in Table S4. Additional primers for PCR amplification, screening, and sequencing were designed using WebPrimer (www.candidagenome.org/cgi-bin/compute/web-primer). All chromosomal mutations were confirmed by Sanger sequencing (UAB Heflin Center for Genomic Sciences).

10.1128/mSystems.00453-19.9 TABLE S3 Mutagenic primers. Download Table S3, DOCX file, 0.02 MB. Copyright © 2019 Basu Thakur et al. 2019 Basu Thakur et al. This content is distributed under the terms of the Creative Commons Attribution 4.0 International license.

10.1128/mSystems.00453-19.10 TABLE S4 Primers used for quantitative RT-PCR. Download Table S4, DOCX file, 0.02 MB. Copyright © 2019 Basu Thakur et al. 2019 Basu Thakur et al. This content is distributed under the terms of the Creative Commons Attribution 4.0 International license.

### Data availability.

All strains generated in the course of this work are available from the authors upon request. RNA sequencing data have been deposited in NCBI’s Gene Expression Omnibus (96) and are accessible through GEO Series accession number GSE127961.

10.1128/mSystems.00453-19.1 TEXT S1 Supplemental methods and references. Download Text S1, DOCX file, 0.03 MB. Copyright © 2019 Basu Thakur et al. 2019 Basu Thakur et al. This content is distributed under the terms of the Creative Commons Attribution 4.0 International license.
