# Supplementary material for: Complex Responses to Hydrogen Peroxide and Hypochlorous Acid by the Probiotic Bacterium Lactobacillus reuteri
Source: mSystems. 2019 Sep 3;4(5):e00453-19. doi: 10.1128/mSystems.00453-19 (PMC6722424; doi:10.1128/mSystems.00453-19)
Supplement: TABLE S2 [file mSystems.00453-19-st002.docx]

| A | B | C | D | E | F |
| --- | --- | --- | --- | --- | --- |
| Comparison | Stressor | Minutes | Direction | Gene | Symbol |
| H2O2_5min_vs_baseline | H2O2 | 5 | UP | LAR_RS00215 | osmC |
| H2O2_5min_vs_baseline | H2O2 | 5 | UP | LAR_RS00345 | noxE |
| H2O2_5min_vs_baseline | H2O2 | 5 | UP | LAR_RS00975 | msrB |
| H2O2_5min_vs_baseline | H2O2 | 5 | UP | LAR_RS02625 | oxc |
| H2O2_5min_vs_baseline | H2O2 | 5 | UP | LAR_RS02680 | cydA |
| H2O2_5min_vs_baseline | H2O2 | 5 | UP | LAR_RS02685 | cydB |
| H2O2_5min_vs_baseline | H2O2 | 5 | UP | LAR_RS02690 | cydD |
| H2O2_5min_vs_baseline | H2O2 | 5 | UP | LAR_RS02695 | cydC |
| H2O2_5min_vs_baseline | H2O2 | 5 | UP | LAR_RS03125 |  |
| H2O2_5min_vs_baseline | H2O2 | 5 | UP | LAR_RS04130 |  |
| H2O2_5min_vs_baseline | H2O2 | 5 | UP | LAR_RS04235 |  |
| H2O2_5min_vs_baseline | H2O2 | 5 | UP | LAR_RS04240 |  |
| H2O2_5min_vs_baseline | H2O2 | 5 | UP | LAR_RS04890 | nrdI |
| H2O2_5min_vs_baseline | H2O2 | 5 | UP | LAR_RS05795 | ahpF |
| H2O2_5min_vs_baseline | H2O2 | 5 | UP | LAR_RS05800 | ahpC |
| H2O2_5min_vs_baseline | H2O2 | 5 | UP | LAR_RS06965 | ycnB |
| H2O2_5min_vs_baseline | H2O2 | 5 | UP | LAR_RS06970 | perR |
| H2O2_5min_vs_baseline | H2O2 | 5 | UP | LAR_RS07565 |  |
| H2O2_5min_vs_baseline | H2O2 | 5 | UP | LAR_RS08080 | pcl1 |
| H2O2_5min_vs_baseline | H2O2 | 5 | UP | LAR_RS08085 | pcl2 |
| H2O2_5min_vs_baseline | H2O2 | 5 | UP | LAR_RS08735 |  |
| H2O2_5min_vs_baseline | H2O2 | 5 | UP | LAR_RS08820 |  |
| H2O2_5min_vs_baseline | H2O2 | 5 | UP | LAR_RS08880 | gatC |
| H2O2_5min_vs_baseline | H2O2 | 5 | UP | LAR_RS09770 |  |
| H2O2_5min_vs_baseline | H2O2 | 5 | DOWN | LAR_RS04695 | sigH |
| H2O2_5min_vs_baseline | H2O2 | 5 | DOWN | LAR_RS05330 | narG |
| H2O2_5min_vs_baseline | H2O2 | 5 | DOWN | LAR_RS05335 | moeB |
| H2O2_5min_vs_baseline | H2O2 | 5 | DOWN | LAR_RS05390 | narK |
| H2O2_5min_vs_baseline | H2O2 | 5 | DOWN | LAR_RS05395 | moaD |
| H2O2_5min_vs_baseline | H2O2 | 5 | DOWN | LAR_RS05400 | moaE |
| H2O2_5min_vs_baseline | H2O2 | 5 | DOWN | LAR_RS05455 |  |
| H2O2_5min_vs_baseline | H2O2 | 5 | DOWN | LAR_RS05460 |  |
| H2O2_5min_vs_baseline | H2O2 | 5 | DOWN | LAR_RS05745 |  |
| H2O2_5min_vs_baseline | H2O2 | 5 | DOWN | LAR_RS07015 | preA |
| H2O2_5min_vs_baseline | H2O2 | 5 | DOWN | LAR_RS07980 |  |
| H2O2_15min_vs_baseline | H2O2 | 15 | UP | LAR_RS00025 | gyrB |
| H2O2_15min_vs_baseline | H2O2 | 15 | UP | LAR_RS00030 | gyrA |
| H2O2_15min_vs_baseline | H2O2 | 15 | UP | LAR_RS00065 |  |
| H2O2_15min_vs_baseline | H2O2 | 15 | UP | LAR_RS00070 |  |
| H2O2_15min_vs_baseline | H2O2 | 15 | UP | LAR_RS00170 |  |
| H2O2_15min_vs_baseline | H2O2 | 15 | UP | LAR_RS00175 | proC |
| H2O2_15min_vs_baseline | H2O2 | 15 | UP | LAR_RS00215 | osmC |
| H2O2_15min_vs_baseline | H2O2 | 15 | UP | LAR_RS00235 | clpL |
| H2O2_15min_vs_baseline | H2O2 | 15 | UP | LAR_RS00255 | addB |
| H2O2_15min_vs_baseline | H2O2 | 15 | UP | LAR_RS00345 | noxE |
| H2O2_15min_vs_baseline | H2O2 | 15 | UP | LAR_RS00660 | fhs |
| H2O2_15min_vs_baseline | H2O2 | 15 | UP | LAR_RS00805 |  |
| H2O2_15min_vs_baseline | H2O2 | 15 | UP | LAR_RS00855 |  |
| H2O2_15min_vs_baseline | H2O2 | 15 | UP | LAR_RS00860 |  |
| H2O2_15min_vs_baseline | H2O2 | 15 | UP | LAR_RS00910 |  |
| H2O2_15min_vs_baseline | H2O2 | 15 | UP | LAR_RS00915 | rclA |
| H2O2_15min_vs_baseline | H2O2 | 15 | UP | LAR_RS00925 |  |
| H2O2_15min_vs_baseline | H2O2 | 15 | UP | LAR_RS00975 | msrB |
| H2O2_15min_vs_baseline | H2O2 | 15 | UP | LAR_RS01220 | srtA |
| H2O2_15min_vs_baseline | H2O2 | 15 | UP | LAR_RS01450 |  |
| H2O2_15min_vs_baseline | H2O2 | 15 | UP | LAR_RS01540 | gpmA |
| H2O2_15min_vs_baseline | H2O2 | 15 | UP | LAR_RS01545 |  |
| H2O2_15min_vs_baseline | H2O2 | 15 | UP | LAR_RS01595 |  |
| H2O2_15min_vs_baseline | H2O2 | 15 | UP | LAR_RS01660 |  |
| H2O2_15min_vs_baseline | H2O2 | 15 | UP | LAR_RS01670 | mscL |
| H2O2_15min_vs_baseline | H2O2 | 15 | UP | LAR_RS01765 | ppx1 |
| H2O2_15min_vs_baseline | H2O2 | 15 | UP | LAR_RS01770 | ppk1 |
| H2O2_15min_vs_baseline | H2O2 | 15 | UP | LAR_RS01975 | trxB |
| H2O2_15min_vs_baseline | H2O2 | 15 | UP | LAR_RS02010 | uvrB |
| H2O2_15min_vs_baseline | H2O2 | 15 | UP | LAR_RS02015 | uvrA |
| H2O2_15min_vs_baseline | H2O2 | 15 | UP | LAR_RS02190 |  |
| H2O2_15min_vs_baseline | H2O2 | 15 | UP | LAR_RS02195 |  |
| H2O2_15min_vs_baseline | H2O2 | 15 | UP | LAR_RS02200 |  |
| H2O2_15min_vs_baseline | H2O2 | 15 | UP | LAR_RS02285 | copA3 |
| H2O2_15min_vs_baseline | H2O2 | 15 | UP | LAR_RS02290 | copA |
| H2O2_15min_vs_baseline | H2O2 | 15 | UP | LAR_RS02370 |  |
| H2O2_15min_vs_baseline | H2O2 | 15 | UP | LAR_RS02400 |  |
| H2O2_15min_vs_baseline | H2O2 | 15 | UP | LAR_RS02415 | tdk |
| H2O2_15min_vs_baseline | H2O2 | 15 | UP | LAR_RS02420 | prfA |
| H2O2_15min_vs_baseline | H2O2 | 15 | UP | LAR_RS02425 |  |
| H2O2_15min_vs_baseline | H2O2 | 15 | UP | LAR_RS02605 |  |
| H2O2_15min_vs_baseline | H2O2 | 15 | UP | LAR_RS02625 | oxc |
| H2O2_15min_vs_baseline | H2O2 | 15 | UP | LAR_RS02680 | cydA |
| H2O2_15min_vs_baseline | H2O2 | 15 | UP | LAR_RS02685 | cydB |
| H2O2_15min_vs_baseline | H2O2 | 15 | UP | LAR_RS02690 | cydD |
| H2O2_15min_vs_baseline | H2O2 | 15 | UP | LAR_RS02850 | trxA |
| H2O2_15min_vs_baseline | H2O2 | 15 | UP | LAR_RS03105 | spxA |
| H2O2_15min_vs_baseline | H2O2 | 15 | UP | LAR_RS03120 | pepDA |
| H2O2_15min_vs_baseline | H2O2 | 15 | UP | LAR_RS03125 |  |
| H2O2_15min_vs_baseline | H2O2 | 15 | UP | LAR_RS03375 | asd |
| H2O2_15min_vs_baseline | H2O2 | 15 | UP | LAR_RS03475 | ftsW |
| H2O2_15min_vs_baseline | H2O2 | 15 | UP | LAR_RS03565 | uvrC |
| H2O2_15min_vs_baseline | H2O2 | 15 | UP | LAR_RS03570 | obg |
| H2O2_15min_vs_baseline | H2O2 | 15 | UP | LAR_RS04130 |  |
| H2O2_15min_vs_baseline | H2O2 | 15 | UP | LAR_RS04235 |  |
| H2O2_15min_vs_baseline | H2O2 | 15 | UP | LAR_RS04755 |  |
| H2O2_15min_vs_baseline | H2O2 | 15 | UP | LAR_RS04895 | ypsC |
| H2O2_15min_vs_baseline | H2O2 | 15 | UP | LAR_RS05370 | nreC |
| H2O2_15min_vs_baseline | H2O2 | 15 | UP | LAR_RS05680 |  |
| H2O2_15min_vs_baseline | H2O2 | 15 | UP | LAR_RS05685 |  |
| H2O2_15min_vs_baseline | H2O2 | 15 | UP | LAR_RS05775 |  |
| H2O2_15min_vs_baseline | H2O2 | 15 | UP | LAR_RS05795 | ahpF |
| H2O2_15min_vs_baseline | H2O2 | 15 | UP | LAR_RS05800 | ahpC |
| H2O2_15min_vs_baseline | H2O2 | 15 | UP | LAR_RS05825 | rpsN |
| H2O2_15min_vs_baseline | H2O2 | 15 | UP | LAR_RS05900 |  |
| H2O2_15min_vs_baseline | H2O2 | 15 | UP | LAR_RS06175 |  |
| H2O2_15min_vs_baseline | H2O2 | 15 | UP | LAR_RS06180 |  |
| H2O2_15min_vs_baseline | H2O2 | 15 | UP | LAR_RS06190 |  |
| H2O2_15min_vs_baseline | H2O2 | 15 | UP | LAR_RS06340 |  |
| H2O2_15min_vs_baseline | H2O2 | 15 | UP | LAR_RS06370 | xseA |
| H2O2_15min_vs_baseline | H2O2 | 15 | UP | LAR_RS06375 | folD |
| H2O2_15min_vs_baseline | H2O2 | 15 | UP | LAR_RS06430 |  |
| H2O2_15min_vs_baseline | H2O2 | 15 | UP | LAR_RS06460 | patA |
| H2O2_15min_vs_baseline | H2O2 | 15 | UP | LAR_RS06555 |  |
| H2O2_15min_vs_baseline | H2O2 | 15 | UP | LAR_RS06905 |  |
| H2O2_15min_vs_baseline | H2O2 | 15 | UP | LAR_RS06965 | ycnB |
| H2O2_15min_vs_baseline | H2O2 | 15 | UP | LAR_RS06970 | perR |
| H2O2_15min_vs_baseline | H2O2 | 15 | UP | LAR_RS07000 | hsp20 |
| H2O2_15min_vs_baseline | H2O2 | 15 | UP | LAR_RS07005 |  |
| H2O2_15min_vs_baseline | H2O2 | 15 | UP | LAR_RS07020 | xthA |
| H2O2_15min_vs_baseline | H2O2 | 15 | UP | LAR_RS07060 |  |
| H2O2_15min_vs_baseline | H2O2 | 15 | UP | LAR_RS07440 | map |
| H2O2_15min_vs_baseline | H2O2 | 15 | UP | LAR_RS07495 | gshA |
| H2O2_15min_vs_baseline | H2O2 | 15 | UP | LAR_RS07510 |  |
| H2O2_15min_vs_baseline | H2O2 | 15 | UP | LAR_RS07515 |  |
| H2O2_15min_vs_baseline | H2O2 | 15 | UP | LAR_RS07525 |  |
| H2O2_15min_vs_baseline | H2O2 | 15 | UP | LAR_RS07530 |  |
| H2O2_15min_vs_baseline | H2O2 | 15 | UP | LAR_RS07550 | ogt |
| H2O2_15min_vs_baseline | H2O2 | 15 | UP | LAR_RS07555 |  |
| H2O2_15min_vs_baseline | H2O2 | 15 | UP | LAR_RS07560 |  |
| H2O2_15min_vs_baseline | H2O2 | 15 | UP | LAR_RS07565 |  |
| H2O2_15min_vs_baseline | H2O2 | 15 | UP | LAR_RS08080 | pcl1 |
| H2O2_15min_vs_baseline | H2O2 | 15 | UP | LAR_RS08085 | pcl2 |
| H2O2_15min_vs_baseline | H2O2 | 15 | UP | LAR_RS08205 | mleS |
| H2O2_15min_vs_baseline | H2O2 | 15 | UP | LAR_RS08215 | ppiB |
| H2O2_15min_vs_baseline | H2O2 | 15 | UP | LAR_RS08240 |  |
| H2O2_15min_vs_baseline | H2O2 | 15 | UP | LAR_RS08395 |  |
| H2O2_15min_vs_baseline | H2O2 | 15 | UP | LAR_RS08400 |  |
| H2O2_15min_vs_baseline | H2O2 | 15 | UP | LAR_RS08695 |  |
| H2O2_15min_vs_baseline | H2O2 | 15 | UP | LAR_RS08735 |  |
| H2O2_15min_vs_baseline | H2O2 | 15 | UP | LAR_RS08780 |  |
| H2O2_15min_vs_baseline | H2O2 | 15 | UP | LAR_RS08820 |  |
| H2O2_15min_vs_baseline | H2O2 | 15 | UP | LAR_RS08880 | gatC |
| H2O2_15min_vs_baseline | H2O2 | 15 | UP | LAR_RS08900 |  |
| H2O2_15min_vs_baseline | H2O2 | 15 | UP | LAR_RS08950 |  |
| H2O2_15min_vs_baseline | H2O2 | 15 | UP | LAR_RS09370 | uspA |
| H2O2_15min_vs_baseline | H2O2 | 15 | UP | LAR_RS09555 |  |
| H2O2_15min_vs_baseline | H2O2 | 15 | UP | LAR_RS09665 | corA |
| H2O2_15min_vs_baseline | H2O2 | 15 | UP | LAR_RS09670 | pepDA |
| H2O2_15min_vs_baseline | H2O2 | 15 | UP | LAR_RS09730 | gldA |
| H2O2_15min_vs_baseline | H2O2 | 15 | UP | LAR_RS09765 | nnr |
| H2O2_15min_vs_baseline | H2O2 | 15 | UP | LAR_RS09770 |  |
| H2O2_15min_vs_baseline | H2O2 | 15 | UP | LAR_RS09805 |  |
| H2O2_15min_vs_baseline | H2O2 | 15 | UP | LAR_RS09885 | metE2 |
| H2O2_15min_vs_baseline | H2O2 | 15 | UP | LAR_RS09890 |  |
| H2O2_15min_vs_baseline | H2O2 | 15 | UP | LAR_RS09895 |  |
| H2O2_15min_vs_baseline | H2O2 | 15 | UP | LAR_RS09940 | pepC |
| H2O2_15min_vs_baseline | H2O2 | 15 | UP | LAR_RS09945 |  |
| H2O2_15min_vs_baseline | H2O2 | 15 | UP | LAR_RS10160 |  |
| H2O2_15min_vs_baseline | H2O2 | 15 | UP | LAR_RS10190 |  |
| H2O2_15min_vs_baseline | H2O2 | 15 | UP | LAR_RS10230 | jag |
| H2O2_15min_vs_baseline | H2O2 | 15 | DOWN | LAR_RS00090 |  |
| H2O2_15min_vs_baseline | H2O2 | 15 | DOWN | LAR_RS00165 | glcU |
| H2O2_15min_vs_baseline | H2O2 | 15 | DOWN | LAR_RS00295 | ywaC |
| H2O2_15min_vs_baseline | H2O2 | 15 | DOWN | LAR_RS00315 | gshA |
| H2O2_15min_vs_baseline | H2O2 | 15 | DOWN | LAR_RS00335 | glpQ |
| H2O2_15min_vs_baseline | H2O2 | 15 | DOWN | LAR_RS00405 |  |
| H2O2_15min_vs_baseline | H2O2 | 15 | DOWN | LAR_RS00450 | malR3 |
| H2O2_15min_vs_baseline | H2O2 | 15 | DOWN | LAR_RS00455 | rumA |
| H2O2_15min_vs_baseline | H2O2 | 15 | DOWN | LAR_RS00510 | serP |
| H2O2_15min_vs_baseline | H2O2 | 15 | DOWN | LAR_RS00565 | deoC |
| H2O2_15min_vs_baseline | H2O2 | 15 | DOWN | LAR_RS00590 | guaB |
| H2O2_15min_vs_baseline | H2O2 | 15 | DOWN | LAR_RS00620 | pyrB |
| H2O2_15min_vs_baseline | H2O2 | 15 | DOWN | LAR_RS00630 | pyrD |
| H2O2_15min_vs_baseline | H2O2 | 15 | DOWN | LAR_RS00635 | pyrF |
| H2O2_15min_vs_baseline | H2O2 | 15 | DOWN | LAR_RS00640 | pyrE |
| H2O2_15min_vs_baseline | H2O2 | 15 | DOWN | LAR_RS00745 | tyrS |
| H2O2_15min_vs_baseline | H2O2 | 15 | DOWN | LAR_RS00810 | pncB |
| H2O2_15min_vs_baseline | H2O2 | 15 | DOWN | LAR_RS00995 | metN |
| H2O2_15min_vs_baseline | H2O2 | 15 | DOWN | LAR_RS01000 | metQ |
| H2O2_15min_vs_baseline | H2O2 | 15 | DOWN | LAR_RS01010 |  |
| H2O2_15min_vs_baseline | H2O2 | 15 | DOWN | LAR_RS01020 |  |
| H2O2_15min_vs_baseline | H2O2 | 15 | DOWN | LAR_RS01025 |  |
| H2O2_15min_vs_baseline | H2O2 | 15 | DOWN | LAR_RS01090 | metG |
| H2O2_15min_vs_baseline | H2O2 | 15 | DOWN | LAR_RS01135 | purR |
| H2O2_15min_vs_baseline | H2O2 | 15 | DOWN | LAR_RS01140 | glmU |
| H2O2_15min_vs_baseline | H2O2 | 15 | DOWN | LAR_RS01325 | codB |
| H2O2_15min_vs_baseline | H2O2 | 15 | DOWN | LAR_RS01335 | hpaH |
| H2O2_15min_vs_baseline | H2O2 | 15 | DOWN | LAR_RS01375 | hpt |
| H2O2_15min_vs_baseline | H2O2 | 15 | DOWN | LAR_RS01400 |  |
| H2O2_15min_vs_baseline | H2O2 | 15 | DOWN | LAR_RS01500 | pncB |
| H2O2_15min_vs_baseline | H2O2 | 15 | DOWN | LAR_RS01620 | nusG |
| H2O2_15min_vs_baseline | H2O2 | 15 | DOWN | LAR_RS01635 |  |
| H2O2_15min_vs_baseline | H2O2 | 15 | DOWN | LAR_RS01640 |  |
| H2O2_15min_vs_baseline | H2O2 | 15 | DOWN | LAR_RS01705 | nrdH |
| H2O2_15min_vs_baseline | H2O2 | 15 | DOWN | LAR_RS01850 | rex |
| H2O2_15min_vs_baseline | H2O2 | 15 | DOWN | LAR_RS01900 |  |
| H2O2_15min_vs_baseline | H2O2 | 15 | DOWN | LAR_RS02140 | dnaQ |
| H2O2_15min_vs_baseline | H2O2 | 15 | DOWN | LAR_RS02150 | rbsK |
| H2O2_15min_vs_baseline | H2O2 | 15 | DOWN | LAR_RS02155 | rbsD |
| H2O2_15min_vs_baseline | H2O2 | 15 | DOWN | LAR_RS02220 | glcU |
| H2O2_15min_vs_baseline | H2O2 | 15 | DOWN | LAR_RS02225 | maa |
| H2O2_15min_vs_baseline | H2O2 | 15 | DOWN | LAR_RS02245 |  |
| H2O2_15min_vs_baseline | H2O2 | 15 | DOWN | LAR_RS02460 | pyrP |
| H2O2_15min_vs_baseline | H2O2 | 15 | DOWN | LAR_RS02610 |  |
| H2O2_15min_vs_baseline | H2O2 | 15 | DOWN | LAR_RS02615 |  |
| H2O2_15min_vs_baseline | H2O2 | 15 | DOWN | LAR_RS02675 | valS |
| H2O2_15min_vs_baseline | H2O2 | 15 | DOWN | LAR_RS02730 |  |
| H2O2_15min_vs_baseline | H2O2 | 15 | DOWN | LAR_RS02735 |  |
| H2O2_15min_vs_baseline | H2O2 | 15 | DOWN | LAR_RS02740 |  |
| H2O2_15min_vs_baseline | H2O2 | 15 | DOWN | LAR_RS02885 | mscS |
| H2O2_15min_vs_baseline | H2O2 | 15 | DOWN | LAR_RS02960 |  |
| H2O2_15min_vs_baseline | H2O2 | 15 | DOWN | LAR_RS03080 | NA |
| H2O2_15min_vs_baseline | H2O2 | 15 | DOWN | LAR_RS03335 | dapF |
| H2O2_15min_vs_baseline | H2O2 | 15 | DOWN | LAR_RS03340 | lysC |
| H2O2_15min_vs_baseline | H2O2 | 15 | DOWN | LAR_RS03440 | pdhA |
| H2O2_15min_vs_baseline | H2O2 | 15 | DOWN | LAR_RS03445 | pdhB |
| H2O2_15min_vs_baseline | H2O2 | 15 | DOWN | LAR_RS03505 | comEB |
| H2O2_15min_vs_baseline | H2O2 | 15 | DOWN | LAR_RS03510 | comEC |
| H2O2_15min_vs_baseline | H2O2 | 15 | DOWN | LAR_RS03530 |  |
| H2O2_15min_vs_baseline | H2O2 | 15 | DOWN | LAR_RS03880 |  |
| H2O2_15min_vs_baseline | H2O2 | 15 | DOWN | LAR_RS04020 | patA |
| H2O2_15min_vs_baseline | H2O2 | 15 | DOWN | LAR_RS04600 |  |
| H2O2_15min_vs_baseline | H2O2 | 15 | DOWN | LAR_RS04680 | kch |
| H2O2_15min_vs_baseline | H2O2 | 15 | DOWN | LAR_RS04685 | yfhO |
| H2O2_15min_vs_baseline | H2O2 | 15 | DOWN | LAR_RS04695 | sigH |
| H2O2_15min_vs_baseline | H2O2 | 15 | DOWN | LAR_RS04820 | melA |
| H2O2_15min_vs_baseline | H2O2 | 15 | DOWN | LAR_RS04875 | recU |
| H2O2_15min_vs_baseline | H2O2 | 15 | DOWN | LAR_RS05065 |  |
| H2O2_15min_vs_baseline | H2O2 | 15 | DOWN | LAR_RS05290 |  |
| H2O2_15min_vs_baseline | H2O2 | 15 | DOWN | LAR_RS05300 |  |
| H2O2_15min_vs_baseline | H2O2 | 15 | DOWN | LAR_RS05320 | narJ |
| H2O2_15min_vs_baseline | H2O2 | 15 | DOWN | LAR_RS05325 | narH |
| H2O2_15min_vs_baseline | H2O2 | 15 | DOWN | LAR_RS05330 | narG |
| H2O2_15min_vs_baseline | H2O2 | 15 | DOWN | LAR_RS05335 | moeB |
| H2O2_15min_vs_baseline | H2O2 | 15 | DOWN | LAR_RS05375 |  |
| H2O2_15min_vs_baseline | H2O2 | 15 | DOWN | LAR_RS05380 |  |
| H2O2_15min_vs_baseline | H2O2 | 15 | DOWN | LAR_RS05385 |  |
| H2O2_15min_vs_baseline | H2O2 | 15 | DOWN | LAR_RS05390 | narK |
| H2O2_15min_vs_baseline | H2O2 | 15 | DOWN | LAR_RS05395 | moaD |
| H2O2_15min_vs_baseline | H2O2 | 15 | DOWN | LAR_RS05400 | moaE |
| H2O2_15min_vs_baseline | H2O2 | 15 | DOWN | LAR_RS05455 |  |
| H2O2_15min_vs_baseline | H2O2 | 15 | DOWN | LAR_RS05460 |  |
| H2O2_15min_vs_baseline | H2O2 | 15 | DOWN | LAR_RS05465 |  |
| H2O2_15min_vs_baseline | H2O2 | 15 | DOWN | LAR_RS05470 |  |
| H2O2_15min_vs_baseline | H2O2 | 15 | DOWN | LAR_RS05475 |  |
| H2O2_15min_vs_baseline | H2O2 | 15 | DOWN | LAR_RS05490 |  |
| H2O2_15min_vs_baseline | H2O2 | 15 | DOWN | LAR_RS05585 |  |
| H2O2_15min_vs_baseline | H2O2 | 15 | DOWN | LAR_RS05590 | yhcR |
| H2O2_15min_vs_baseline | H2O2 | 15 | DOWN | LAR_RS05705 | rfbD |
| H2O2_15min_vs_baseline | H2O2 | 15 | DOWN | LAR_RS05745 |  |
| H2O2_15min_vs_baseline | H2O2 | 15 | DOWN | LAR_RS05875 | lacI |
| H2O2_15min_vs_baseline | H2O2 | 15 | DOWN | LAR_RS06250 | rnc |
| H2O2_15min_vs_baseline | H2O2 | 15 | DOWN | LAR_RS06330 | rpoZ |
| H2O2_15min_vs_baseline | H2O2 | 15 | DOWN | LAR_RS06335 | gmk |
| H2O2_15min_vs_baseline | H2O2 | 15 | DOWN | LAR_RS06540 |  |
| H2O2_15min_vs_baseline | H2O2 | 15 | DOWN | LAR_RS06550 | pheS |
| H2O2_15min_vs_baseline | H2O2 | 15 | DOWN | LAR_RS06570 | acyP |
| H2O2_15min_vs_baseline | H2O2 | 15 | DOWN | LAR_RS06575 | spoIIIJ |
| H2O2_15min_vs_baseline | H2O2 | 15 | DOWN | LAR_RS06580 | def |
| H2O2_15min_vs_baseline | H2O2 | 15 | DOWN | LAR_RS06585 | matE |
| H2O2_15min_vs_baseline | H2O2 | 15 | DOWN | LAR_RS06805 | pbp2A |
| H2O2_15min_vs_baseline | H2O2 | 15 | DOWN | LAR_RS06845 |  |
| H2O2_15min_vs_baseline | H2O2 | 15 | DOWN | LAR_RS06850 | folC |
| H2O2_15min_vs_baseline | H2O2 | 15 | DOWN | LAR_RS06855 | folE |
| H2O2_15min_vs_baseline | H2O2 | 15 | DOWN | LAR_RS06860 | folK |
| H2O2_15min_vs_baseline | H2O2 | 15 | DOWN | LAR_RS06865 | folB |
| H2O2_15min_vs_baseline | H2O2 | 15 | DOWN | LAR_RS06955 | tag |
| H2O2_15min_vs_baseline | H2O2 | 15 | DOWN | LAR_RS07010 | preT |
| H2O2_15min_vs_baseline | H2O2 | 15 | DOWN | LAR_RS07015 | preA |
| H2O2_15min_vs_baseline | H2O2 | 15 | DOWN | LAR_RS07040 |  |
| H2O2_15min_vs_baseline | H2O2 | 15 | DOWN | LAR_RS07045 | nrdG |
| H2O2_15min_vs_baseline | H2O2 | 15 | DOWN | LAR_RS07130 | lafC |
| H2O2_15min_vs_baseline | H2O2 | 15 | DOWN | LAR_RS07135 | cpoA |
| H2O2_15min_vs_baseline | H2O2 | 15 | DOWN | LAR_RS07140 | lafA |
| H2O2_15min_vs_baseline | H2O2 | 15 | DOWN | LAR_RS07165 | panT |
| H2O2_15min_vs_baseline | H2O2 | 15 | DOWN | LAR_RS07185 | lp_2742 |
| H2O2_15min_vs_baseline | H2O2 | 15 | DOWN | LAR_RS07235 |  |
| H2O2_15min_vs_baseline | H2O2 | 15 | DOWN | LAR_RS07240 |  |
| H2O2_15min_vs_baseline | H2O2 | 15 | DOWN | LAR_RS07335 |  |
| H2O2_15min_vs_baseline | H2O2 | 15 | DOWN | LAR_RS07420 |  |
| H2O2_15min_vs_baseline | H2O2 | 15 | DOWN | LAR_RS07610 | cylB |
| H2O2_15min_vs_baseline | H2O2 | 15 | DOWN | LAR_RS07615 |  |
| H2O2_15min_vs_baseline | H2O2 | 15 | DOWN | LAR_RS07620 |  |
| H2O2_15min_vs_baseline | H2O2 | 15 | DOWN | LAR_RS07790 | cbiO |
| H2O2_15min_vs_baseline | H2O2 | 15 | DOWN | LAR_RS07960 | comC |
| H2O2_15min_vs_baseline | H2O2 | 15 | DOWN | LAR_RS07980 |  |
| H2O2_15min_vs_baseline | H2O2 | 15 | DOWN | LAR_RS08030 | dnk |
| H2O2_15min_vs_baseline | H2O2 | 15 | DOWN | LAR_RS08120 | rnj |
| H2O2_15min_vs_baseline | H2O2 | 15 | DOWN | LAR_RS08135 |  |
| H2O2_15min_vs_baseline | H2O2 | 15 | DOWN | LAR_RS08145 | rluD |
| H2O2_15min_vs_baseline | H2O2 | 15 | DOWN | LAR_RS08270 | arcD |
| H2O2_15min_vs_baseline | H2O2 | 15 | DOWN | LAR_RS08300 | cysK |
| H2O2_15min_vs_baseline | H2O2 | 15 | DOWN | LAR_RS08405 |  |
| H2O2_15min_vs_baseline | H2O2 | 15 | DOWN | LAR_RS08455 | metQ |
| H2O2_15min_vs_baseline | H2O2 | 15 | DOWN | LAR_RS08460 | luxS |
| H2O2_15min_vs_baseline | H2O2 | 15 | DOWN | LAR_RS08490 |  |
| H2O2_15min_vs_baseline | H2O2 | 15 | DOWN | LAR_RS08610 | guaA |
| H2O2_15min_vs_baseline | H2O2 | 15 | DOWN | LAR_RS08615 |  |
| H2O2_15min_vs_baseline | H2O2 | 15 | DOWN | LAR_RS08730 | yaaU |
| H2O2_15min_vs_baseline | H2O2 | 15 | DOWN | LAR_RS08815 | iunH |
| H2O2_15min_vs_baseline | H2O2 | 15 | DOWN | LAR_RS08980 |  |
| H2O2_15min_vs_baseline | H2O2 | 15 | DOWN | LAR_RS08995 |  |
| H2O2_15min_vs_baseline | H2O2 | 15 | DOWN | LAR_RS09365 | lacS |
| H2O2_15min_vs_baseline | H2O2 | 15 | DOWN | LAR_RS09405 | galK |
| H2O2_15min_vs_baseline | H2O2 | 15 | DOWN | LAR_RS09445 | pnuN |
| H2O2_15min_vs_baseline | H2O2 | 15 | DOWN | LAR_RS09455 | yxdJ |
| H2O2_15min_vs_baseline | H2O2 | 15 | DOWN | LAR_RS09460 |  |
| H2O2_15min_vs_baseline | H2O2 | 15 | DOWN | LAR_RS09560 |  |
| H2O2_15min_vs_baseline | H2O2 | 15 | DOWN | LAR_RS09570 | brnQ |
| H2O2_15min_vs_baseline | H2O2 | 15 | DOWN | LAR_RS09590 |  |
| H2O2_15min_vs_baseline | H2O2 | 15 | DOWN | LAR_RS09620 |  |
| H2O2_15min_vs_baseline | H2O2 | 15 | DOWN | LAR_RS09625 |  |
| H2O2_15min_vs_baseline | H2O2 | 15 | DOWN | LAR_RS09630 |  |
| H2O2_15min_vs_baseline | H2O2 | 15 | DOWN | LAR_RS09640 | czcR1 |
| H2O2_15min_vs_baseline | H2O2 | 15 | DOWN | LAR_RS09660 | rluD |
| H2O2_15min_vs_baseline | H2O2 | 15 | DOWN | LAR_RS09680 | nhaC2 |
| H2O2_15min_vs_baseline | H2O2 | 15 | DOWN | LAR_RS09795 | scrR |
| H2O2_15min_vs_baseline | H2O2 | 15 | DOWN | LAR_RS09830 | yxeR |
| H2O2_15min_vs_baseline | H2O2 | 15 | DOWN | LAR_RS09835 | mtlD |
| H2O2_15min_vs_baseline | H2O2 | 15 | DOWN | LAR_RS09850 | thrS |
| H2O2_15min_vs_baseline | H2O2 | 15 | DOWN | LAR_RS09960 | asnA |
| H2O2_15min_vs_baseline | H2O2 | 15 | DOWN | LAR_RS09975 |  |
| H2O2_15min_vs_baseline | H2O2 | 15 | DOWN | LAR_RS10030 |  |
| H2O2_15min_vs_baseline | H2O2 | 15 | DOWN | LAR_RS10040 |  |
| H2O2_15min_vs_baseline | H2O2 | 15 | DOWN | LAR_RS10050 | hicD3 |
| H2O2_15min_vs_baseline | H2O2 | 15 | DOWN | LAR_RS10055 | hpaH |
| H2O2_15min_vs_baseline | H2O2 | 15 | DOWN | LAR_RS10135 | ybaK |
| H2O2_15min_vs_baseline | H2O2 | 15 | DOWN | LAR_RS10140 |  |
| H2O2_30min_vs_baseline | H2O2 | 30 | UP | LAR_RS00065 |  |
| H2O2_30min_vs_baseline | H2O2 | 30 | UP | LAR_RS00070 |  |
| H2O2_30min_vs_baseline | H2O2 | 30 | UP | LAR_RS00075 | ppk2 |
| H2O2_30min_vs_baseline | H2O2 | 30 | UP | LAR_RS00170 |  |
| H2O2_30min_vs_baseline | H2O2 | 30 | UP | LAR_RS00175 | proC |
| H2O2_30min_vs_baseline | H2O2 | 30 | UP | LAR_RS00180 | gabD |
| H2O2_30min_vs_baseline | H2O2 | 30 | UP | LAR_RS00210 |  |
| H2O2_30min_vs_baseline | H2O2 | 30 | UP | LAR_RS00215 | osmC |
| H2O2_30min_vs_baseline | H2O2 | 30 | UP | LAR_RS00230 |  |
| H2O2_30min_vs_baseline | H2O2 | 30 | UP | LAR_RS00235 | clpL |
| H2O2_30min_vs_baseline | H2O2 | 30 | UP | LAR_RS00255 | addB |
| H2O2_30min_vs_baseline | H2O2 | 30 | UP | LAR_RS00260 | addA |
| H2O2_30min_vs_baseline | H2O2 | 30 | UP | LAR_RS00270 | atoB |
| H2O2_30min_vs_baseline | H2O2 | 30 | UP | LAR_RS00290 |  |
| H2O2_30min_vs_baseline | H2O2 | 30 | UP | LAR_RS00325 | ycjL |
| H2O2_30min_vs_baseline | H2O2 | 30 | UP | LAR_RS00330 |  |
| H2O2_30min_vs_baseline | H2O2 | 30 | UP | LAR_RS00345 | noxE |
| H2O2_30min_vs_baseline | H2O2 | 30 | UP | LAR_RS00350 | guaC |
| H2O2_30min_vs_baseline | H2O2 | 30 | UP | LAR_RS00360 |  |
| H2O2_30min_vs_baseline | H2O2 | 30 | UP | LAR_RS00420 | clfA |
| H2O2_30min_vs_baseline | H2O2 | 30 | UP | LAR_RS00660 | fhs |
| H2O2_30min_vs_baseline | H2O2 | 30 | UP | LAR_RS00665 | ilvB |
| H2O2_30min_vs_baseline | H2O2 | 30 | UP | LAR_RS00670 | alsD |
| H2O2_30min_vs_baseline | H2O2 | 30 | UP | LAR_RS00675 | purE |
| H2O2_30min_vs_baseline | H2O2 | 30 | UP | LAR_RS00680 | purK |
| H2O2_30min_vs_baseline | H2O2 | 30 | UP | LAR_RS00805 |  |
| H2O2_30min_vs_baseline | H2O2 | 30 | UP | LAR_RS00855 |  |
| H2O2_30min_vs_baseline | H2O2 | 30 | UP | LAR_RS00860 |  |
| H2O2_30min_vs_baseline | H2O2 | 30 | UP | LAR_RS00910 |  |
| H2O2_30min_vs_baseline | H2O2 | 30 | UP | LAR_RS00925 |  |
| H2O2_30min_vs_baseline | H2O2 | 30 | UP | LAR_RS00930 |  |
| H2O2_30min_vs_baseline | H2O2 | 30 | UP | LAR_RS00975 | msrB |
| H2O2_30min_vs_baseline | H2O2 | 30 | UP | LAR_RS01005 |  |
| H2O2_30min_vs_baseline | H2O2 | 30 | UP | LAR_RS01055 | yhdJ |
| H2O2_30min_vs_baseline | H2O2 | 30 | UP | LAR_RS01270 | glsA |
| H2O2_30min_vs_baseline | H2O2 | 30 | UP | LAR_RS01330 | codA |
| H2O2_30min_vs_baseline | H2O2 | 30 | UP | LAR_RS01450 |  |
| H2O2_30min_vs_baseline | H2O2 | 30 | UP | LAR_RS01480 |  |
| H2O2_30min_vs_baseline | H2O2 | 30 | UP | LAR_RS01485 |  |
| H2O2_30min_vs_baseline | H2O2 | 30 | UP | LAR_RS01540 | gpmA |
| H2O2_30min_vs_baseline | H2O2 | 30 | UP | LAR_RS01545 |  |
| H2O2_30min_vs_baseline | H2O2 | 30 | UP | LAR_RS01585 | dut |
| H2O2_30min_vs_baseline | H2O2 | 30 | UP | LAR_RS01595 |  |
| H2O2_30min_vs_baseline | H2O2 | 30 | UP | LAR_RS01600 | cysS |
| H2O2_30min_vs_baseline | H2O2 | 30 | UP | LAR_RS01605 |  |
| H2O2_30min_vs_baseline | H2O2 | 30 | UP | LAR_RS01615 | secE |
| H2O2_30min_vs_baseline | H2O2 | 30 | UP | LAR_RS01660 |  |
| H2O2_30min_vs_baseline | H2O2 | 30 | UP | LAR_RS01670 | mscL |
| H2O2_30min_vs_baseline | H2O2 | 30 | UP | LAR_RS01765 | ppx1 |
| H2O2_30min_vs_baseline | H2O2 | 30 | UP | LAR_RS01770 | ppk1 |
| H2O2_30min_vs_baseline | H2O2 | 30 | UP | LAR_RS01775 | ppx2 |
| H2O2_30min_vs_baseline | H2O2 | 30 | UP | LAR_RS01795 | tsaB |
| H2O2_30min_vs_baseline | H2O2 | 30 | UP | LAR_RS01800 | rimI |
| H2O2_30min_vs_baseline | H2O2 | 30 | UP | LAR_RS01860 | groS |
| H2O2_30min_vs_baseline | H2O2 | 30 | UP | LAR_RS01870 | ybaK |
| H2O2_30min_vs_baseline | H2O2 | 30 | UP | LAR_RS01975 | trxB |
| H2O2_30min_vs_baseline | H2O2 | 30 | UP | LAR_RS01980 | dapE |
| H2O2_30min_vs_baseline | H2O2 | 30 | UP | LAR_RS02000 |  |
| H2O2_30min_vs_baseline | H2O2 | 30 | UP | LAR_RS02010 | uvrB |
| H2O2_30min_vs_baseline | H2O2 | 30 | UP | LAR_RS02015 | uvrA |
| H2O2_30min_vs_baseline | H2O2 | 30 | UP | LAR_RS02080 | proY |
| H2O2_30min_vs_baseline | H2O2 | 30 | UP | LAR_RS02085 | eriC |
| H2O2_30min_vs_baseline | H2O2 | 30 | UP | LAR_RS02165 |  |
| H2O2_30min_vs_baseline | H2O2 | 30 | UP | LAR_RS02170 |  |
| H2O2_30min_vs_baseline | H2O2 | 30 | UP | LAR_RS02180 | glmS |
| H2O2_30min_vs_baseline | H2O2 | 30 | UP | LAR_RS02190 |  |
| H2O2_30min_vs_baseline | H2O2 | 30 | UP | LAR_RS02195 |  |
| H2O2_30min_vs_baseline | H2O2 | 30 | UP | LAR_RS02200 |  |
| H2O2_30min_vs_baseline | H2O2 | 30 | UP | LAR_RS02275 |  |
| H2O2_30min_vs_baseline | H2O2 | 30 | UP | LAR_RS02280 | copA2 |
| H2O2_30min_vs_baseline | H2O2 | 30 | UP | LAR_RS02285 | copA3 |
| H2O2_30min_vs_baseline | H2O2 | 30 | UP | LAR_RS02290 | copA |
| H2O2_30min_vs_baseline | H2O2 | 30 | UP | LAR_RS02370 |  |
| H2O2_30min_vs_baseline | H2O2 | 30 | UP | LAR_RS02415 | tdk |
| H2O2_30min_vs_baseline | H2O2 | 30 | UP | LAR_RS02420 | prfA |
| H2O2_30min_vs_baseline | H2O2 | 30 | UP | LAR_RS02425 |  |
| H2O2_30min_vs_baseline | H2O2 | 30 | UP | LAR_RS02605 |  |
| H2O2_30min_vs_baseline | H2O2 | 30 | UP | LAR_RS02625 | oxc |
| H2O2_30min_vs_baseline | H2O2 | 30 | UP | LAR_RS02680 | cydA |
| H2O2_30min_vs_baseline | H2O2 | 30 | UP | LAR_RS02685 | cydB |
| H2O2_30min_vs_baseline | H2O2 | 30 | UP | LAR_RS02705 | folC |
| H2O2_30min_vs_baseline | H2O2 | 30 | UP | LAR_RS02710 | radC |
| H2O2_30min_vs_baseline | H2O2 | 30 | UP | LAR_RS02745 |  |
| H2O2_30min_vs_baseline | H2O2 | 30 | UP | LAR_RS02770 | recA |
| H2O2_30min_vs_baseline | H2O2 | 30 | UP | LAR_RS02775 |  |
| H2O2_30min_vs_baseline | H2O2 | 30 | UP | LAR_RS02810 |  |
| H2O2_30min_vs_baseline | H2O2 | 30 | UP | LAR_RS02855 |  |
| H2O2_30min_vs_baseline | H2O2 | 30 | UP | LAR_RS02920 | comGA |
| H2O2_30min_vs_baseline | H2O2 | 30 | UP | LAR_RS02925 | comGB |
| H2O2_30min_vs_baseline | H2O2 | 30 | UP | LAR_RS02930 | comGC |
| H2O2_30min_vs_baseline | H2O2 | 30 | UP | LAR_RS02935 |  |
| H2O2_30min_vs_baseline | H2O2 | 30 | UP | LAR_RS02940 |  |
| H2O2_30min_vs_baseline | H2O2 | 30 | UP | LAR_RS02945 |  |
| H2O2_30min_vs_baseline | H2O2 | 30 | UP | LAR_RS03100 | yhgC |
| H2O2_30min_vs_baseline | H2O2 | 30 | UP | LAR_RS03105 | spxA |
| H2O2_30min_vs_baseline | H2O2 | 30 | UP | LAR_RS03115 | coiA |
| H2O2_30min_vs_baseline | H2O2 | 30 | UP | LAR_RS03120 | pepDA |
| H2O2_30min_vs_baseline | H2O2 | 30 | UP | LAR_RS03125 |  |
| H2O2_30min_vs_baseline | H2O2 | 30 | UP | LAR_RS03460 |  |
| H2O2_30min_vs_baseline | H2O2 | 30 | UP | LAR_RS03465 | suhB |
| H2O2_30min_vs_baseline | H2O2 | 30 | UP | LAR_RS03475 | ftsW |
| H2O2_30min_vs_baseline | H2O2 | 30 | UP | LAR_RS03480 |  |
| H2O2_30min_vs_baseline | H2O2 | 30 | UP | LAR_RS03485 | rsmD |
| H2O2_30min_vs_baseline | H2O2 | 30 | UP | LAR_RS03490 | coaD |
| H2O2_30min_vs_baseline | H2O2 | 30 | UP | LAR_RS03565 | uvrC |
| H2O2_30min_vs_baseline | H2O2 | 30 | UP | LAR_RS03570 | obg |
| H2O2_30min_vs_baseline | H2O2 | 30 | UP | LAR_RS03675 | lexA |
| H2O2_30min_vs_baseline | H2O2 | 30 | UP | LAR_RS03695 |  |
| H2O2_30min_vs_baseline | H2O2 | 30 | UP | LAR_RS03700 |  |
| H2O2_30min_vs_baseline | H2O2 | 30 | UP | LAR_RS03705 | ldhA |
| H2O2_30min_vs_baseline | H2O2 | 30 | UP | LAR_RS03810 | hrcA |
| H2O2_30min_vs_baseline | H2O2 | 30 | UP | LAR_RS03945 |  |
| H2O2_30min_vs_baseline | H2O2 | 30 | UP | LAR_RS04050 | dnaE |
| H2O2_30min_vs_baseline | H2O2 | 30 | UP | LAR_RS04130 |  |
| H2O2_30min_vs_baseline | H2O2 | 30 | UP | LAR_RS04235 |  |
| H2O2_30min_vs_baseline | H2O2 | 30 | UP | LAR_RS04250 |  |
| H2O2_30min_vs_baseline | H2O2 | 30 | UP | LAR_RS04280 |  |
| H2O2_30min_vs_baseline | H2O2 | 30 | UP | LAR_RS04285 |  |
| H2O2_30min_vs_baseline | H2O2 | 30 | UP | LAR_RS04310 |  |
| H2O2_30min_vs_baseline | H2O2 | 30 | UP | LAR_RS04315 |  |
| H2O2_30min_vs_baseline | H2O2 | 30 | UP | LAR_RS04350 | recT1 |
| H2O2_30min_vs_baseline | H2O2 | 30 | UP | LAR_RS04605 |  |
| H2O2_30min_vs_baseline | H2O2 | 30 | UP | LAR_RS04705 | menB |
| H2O2_30min_vs_baseline | H2O2 | 30 | UP | LAR_RS04755 |  |
| H2O2_30min_vs_baseline | H2O2 | 30 | UP | LAR_RS04845 | mvaK1 |
| H2O2_30min_vs_baseline | H2O2 | 30 | UP | LAR_RS04880 |  |
| H2O2_30min_vs_baseline | H2O2 | 30 | UP | LAR_RS04895 | ypsC |
| H2O2_30min_vs_baseline | H2O2 | 30 | UP | LAR_RS04950 | waaW |
| H2O2_30min_vs_baseline | H2O2 | 30 | UP | LAR_RS04960 |  |
| H2O2_30min_vs_baseline | H2O2 | 30 | UP | LAR_RS04990 | fldA |
| H2O2_30min_vs_baseline | H2O2 | 30 | UP | LAR_RS05075 | endA |
| H2O2_30min_vs_baseline | H2O2 | 30 | UP | LAR_RS05330 | narG |
| H2O2_30min_vs_baseline | H2O2 | 30 | UP | LAR_RS05335 | moeB |
| H2O2_30min_vs_baseline | H2O2 | 30 | UP | LAR_RS05340 | moaB |
| H2O2_30min_vs_baseline | H2O2 | 30 | UP | LAR_RS05370 | nreC |
| H2O2_30min_vs_baseline | H2O2 | 30 | UP | LAR_RS05390 | narK |
| H2O2_30min_vs_baseline | H2O2 | 30 | UP | LAR_RS05395 | moaD |
| H2O2_30min_vs_baseline | H2O2 | 30 | UP | LAR_RS05400 | moaE |
| H2O2_30min_vs_baseline | H2O2 | 30 | UP | LAR_RS05405 |  |
| H2O2_30min_vs_baseline | H2O2 | 30 | UP | LAR_RS05410 |  |
| H2O2_30min_vs_baseline | H2O2 | 30 | UP | LAR_RS05455 |  |
| H2O2_30min_vs_baseline | H2O2 | 30 | UP | LAR_RS05460 |  |
| H2O2_30min_vs_baseline | H2O2 | 30 | UP | LAR_RS05560 |  |
| H2O2_30min_vs_baseline | H2O2 | 30 | UP | LAR_RS05565 |  |
| H2O2_30min_vs_baseline | H2O2 | 30 | UP | LAR_RS05680 |  |
| H2O2_30min_vs_baseline | H2O2 | 30 | UP | LAR_RS05685 |  |
| H2O2_30min_vs_baseline | H2O2 | 30 | UP | LAR_RS05765 | glpK |
| H2O2_30min_vs_baseline | H2O2 | 30 | UP | LAR_RS05770 | xylH |
| H2O2_30min_vs_baseline | H2O2 | 30 | UP | LAR_RS05775 |  |
| H2O2_30min_vs_baseline | H2O2 | 30 | UP | LAR_RS05795 | ahpF |
| H2O2_30min_vs_baseline | H2O2 | 30 | UP | LAR_RS05800 | ahpC |
| H2O2_30min_vs_baseline | H2O2 | 30 | UP | LAR_RS05825 | rpsN |
| H2O2_30min_vs_baseline | H2O2 | 30 | UP | LAR_RS05830 |  |
| H2O2_30min_vs_baseline | H2O2 | 30 | UP | LAR_RS05900 |  |
| H2O2_30min_vs_baseline | H2O2 | 30 | UP | LAR_RS06065 |  |
| H2O2_30min_vs_baseline | H2O2 | 30 | UP | LAR_RS06175 |  |
| H2O2_30min_vs_baseline | H2O2 | 30 | UP | LAR_RS06180 |  |
| H2O2_30min_vs_baseline | H2O2 | 30 | UP | LAR_RS06190 |  |
| H2O2_30min_vs_baseline | H2O2 | 30 | UP | LAR_RS06195 |  |
| H2O2_30min_vs_baseline | H2O2 | 30 | UP | LAR_RS06325 | coaBC |
| H2O2_30min_vs_baseline | H2O2 | 30 | UP | LAR_RS06340 |  |
| H2O2_30min_vs_baseline | H2O2 | 30 | UP | LAR_RS06345 | recN |
| H2O2_30min_vs_baseline | H2O2 | 30 | UP | LAR_RS06350 | argR |
| H2O2_30min_vs_baseline | H2O2 | 30 | UP | LAR_RS06355 | yqxC |
| H2O2_30min_vs_baseline | H2O2 | 30 | UP | LAR_RS06360 |  |
| H2O2_30min_vs_baseline | H2O2 | 30 | UP | LAR_RS06365 | xseB |
| H2O2_30min_vs_baseline | H2O2 | 30 | UP | LAR_RS06370 | xseA |
| H2O2_30min_vs_baseline | H2O2 | 30 | UP | LAR_RS06375 | folD |
| H2O2_30min_vs_baseline | H2O2 | 30 | UP | LAR_RS06390 |  |
| H2O2_30min_vs_baseline | H2O2 | 30 | UP | LAR_RS06395 | pepP |
| H2O2_30min_vs_baseline | H2O2 | 30 | UP | LAR_RS06400 |  |
| H2O2_30min_vs_baseline | H2O2 | 30 | UP | LAR_RS06460 | patA |
| H2O2_30min_vs_baseline | H2O2 | 30 | UP | LAR_RS06470 | glnA |
| H2O2_30min_vs_baseline | H2O2 | 30 | UP | LAR_RS06520 |  |
| H2O2_30min_vs_baseline | H2O2 | 30 | UP | LAR_RS06710 | murC |
| H2O2_30min_vs_baseline | H2O2 | 30 | UP | LAR_RS06730 | glsA |
| H2O2_30min_vs_baseline | H2O2 | 30 | UP | LAR_RS06750 |  |
| H2O2_30min_vs_baseline | H2O2 | 30 | UP | LAR_RS06755 |  |
| H2O2_30min_vs_baseline | H2O2 | 30 | UP | LAR_RS06795 | sbcD |
| H2O2_30min_vs_baseline | H2O2 | 30 | UP | LAR_RS06875 | NA |
| H2O2_30min_vs_baseline | H2O2 | 30 | UP | LAR_RS06880 |  |
| H2O2_30min_vs_baseline | H2O2 | 30 | UP | LAR_RS06905 |  |
| H2O2_30min_vs_baseline | H2O2 | 30 | UP | LAR_RS06915 | blyA |
| H2O2_30min_vs_baseline | H2O2 | 30 | UP | LAR_RS06940 |  |
| H2O2_30min_vs_baseline | H2O2 | 30 | UP | LAR_RS06945 | leuS |
| H2O2_30min_vs_baseline | H2O2 | 30 | UP | LAR_RS06950 | metK |
| H2O2_30min_vs_baseline | H2O2 | 30 | UP | LAR_RS06965 | ycnB |
| H2O2_30min_vs_baseline | H2O2 | 30 | UP | LAR_RS06990 | lp_3022 |
| H2O2_30min_vs_baseline | H2O2 | 30 | UP | LAR_RS06995 | umuC |
| H2O2_30min_vs_baseline | H2O2 | 30 | UP | LAR_RS07000 | hsp20 |
| H2O2_30min_vs_baseline | H2O2 | 30 | UP | LAR_RS07005 |  |
| H2O2_30min_vs_baseline | H2O2 | 30 | UP | LAR_RS07020 | xthA |
| H2O2_30min_vs_baseline | H2O2 | 30 | UP | LAR_RS07030 |  |
| H2O2_30min_vs_baseline | H2O2 | 30 | UP | LAR_RS07055 |  |
| H2O2_30min_vs_baseline | H2O2 | 30 | UP | LAR_RS07060 |  |
| H2O2_30min_vs_baseline | H2O2 | 30 | UP | LAR_RS07250 |  |
| H2O2_30min_vs_baseline | H2O2 | 30 | UP | LAR_RS07495 | gshA |
| H2O2_30min_vs_baseline | H2O2 | 30 | UP | LAR_RS07510 |  |
| H2O2_30min_vs_baseline | H2O2 | 30 | UP | LAR_RS07515 |  |
| H2O2_30min_vs_baseline | H2O2 | 30 | UP | LAR_RS07525 |  |
| H2O2_30min_vs_baseline | H2O2 | 30 | UP | LAR_RS07530 |  |
| H2O2_30min_vs_baseline | H2O2 | 30 | UP | LAR_RS07535 | cwlA |
| H2O2_30min_vs_baseline | H2O2 | 30 | UP | LAR_RS07550 | ogt |
| H2O2_30min_vs_baseline | H2O2 | 30 | UP | LAR_RS07555 |  |
| H2O2_30min_vs_baseline | H2O2 | 30 | UP | LAR_RS07575 | amt |
| H2O2_30min_vs_baseline | H2O2 | 30 | UP | LAR_RS07585 |  |
| H2O2_30min_vs_baseline | H2O2 | 30 | UP | LAR_RS07640 | flpA |
| H2O2_30min_vs_baseline | H2O2 | 30 | UP | LAR_RS07735 | ligA |
| H2O2_30min_vs_baseline | H2O2 | 30 | UP | LAR_RS07740 | uvrD |
| H2O2_30min_vs_baseline | H2O2 | 30 | UP | LAR_RS08080 | pcl1 |
| H2O2_30min_vs_baseline | H2O2 | 30 | UP | LAR_RS08085 | pcl2 |
| H2O2_30min_vs_baseline | H2O2 | 30 | UP | LAR_RS08125 |  |
| H2O2_30min_vs_baseline | H2O2 | 30 | UP | LAR_RS08205 | mleS |
| H2O2_30min_vs_baseline | H2O2 | 30 | UP | LAR_RS08210 | mleR |
| H2O2_30min_vs_baseline | H2O2 | 30 | UP | LAR_RS08215 | ppiB |
| H2O2_30min_vs_baseline | H2O2 | 30 | UP | LAR_RS08240 |  |
| H2O2_30min_vs_baseline | H2O2 | 30 | UP | LAR_RS08255 | scrP |
| H2O2_30min_vs_baseline | H2O2 | 30 | UP | LAR_RS08285 |  |
| H2O2_30min_vs_baseline | H2O2 | 30 | UP | LAR_RS08395 |  |
| H2O2_30min_vs_baseline | H2O2 | 30 | UP | LAR_RS08400 |  |
| H2O2_30min_vs_baseline | H2O2 | 30 | UP | LAR_RS08430 | ptpA |
| H2O2_30min_vs_baseline | H2O2 | 30 | UP | LAR_RS08510 |  |
| H2O2_30min_vs_baseline | H2O2 | 30 | UP | LAR_RS08525 |  |
| H2O2_30min_vs_baseline | H2O2 | 30 | UP | LAR_RS08530 |  |
| H2O2_30min_vs_baseline | H2O2 | 30 | UP | LAR_RS08685 | yhgE |
| H2O2_30min_vs_baseline | H2O2 | 30 | UP | LAR_RS08690 |  |
| H2O2_30min_vs_baseline | H2O2 | 30 | UP | LAR_RS08695 |  |
| H2O2_30min_vs_baseline | H2O2 | 30 | UP | LAR_RS08700 |  |
| H2O2_30min_vs_baseline | H2O2 | 30 | UP | LAR_RS08715 | gmk |
| H2O2_30min_vs_baseline | H2O2 | 30 | UP | LAR_RS08735 |  |
| H2O2_30min_vs_baseline | H2O2 | 30 | UP | LAR_RS08780 |  |
| H2O2_30min_vs_baseline | H2O2 | 30 | UP | LAR_RS08810 | trxB |
| H2O2_30min_vs_baseline | H2O2 | 30 | UP | LAR_RS08820 |  |
| H2O2_30min_vs_baseline | H2O2 | 30 | UP | LAR_RS08880 | gatC |
| H2O2_30min_vs_baseline | H2O2 | 30 | UP | LAR_RS08900 |  |
| H2O2_30min_vs_baseline | H2O2 | 30 | UP | LAR_RS08905 |  |
| H2O2_30min_vs_baseline | H2O2 | 30 | UP | LAR_RS08950 |  |
| H2O2_30min_vs_baseline | H2O2 | 30 | UP | LAR_RS09135 | cbiA |
| H2O2_30min_vs_baseline | H2O2 | 30 | UP | LAR_RS09140 | cobD |
| H2O2_30min_vs_baseline | H2O2 | 30 | UP | LAR_RS09295 |  |
| H2O2_30min_vs_baseline | H2O2 | 30 | UP | LAR_RS09300 |  |
| H2O2_30min_vs_baseline | H2O2 | 30 | UP | LAR_RS09360 |  |
| H2O2_30min_vs_baseline | H2O2 | 30 | UP | LAR_RS09485 | cls |
| H2O2_30min_vs_baseline | H2O2 | 30 | UP | LAR_RS09580 |  |
| H2O2_30min_vs_baseline | H2O2 | 30 | UP | LAR_RS09665 | corA |
| H2O2_30min_vs_baseline | H2O2 | 30 | UP | LAR_RS09670 | pepDA |
| H2O2_30min_vs_baseline | H2O2 | 30 | UP | LAR_RS09740 | dacC |
| H2O2_30min_vs_baseline | H2O2 | 30 | UP | LAR_RS09765 | nnr |
| H2O2_30min_vs_baseline | H2O2 | 30 | UP | LAR_RS09770 |  |
| H2O2_30min_vs_baseline | H2O2 | 30 | UP | LAR_RS09805 |  |
| H2O2_30min_vs_baseline | H2O2 | 30 | UP | LAR_RS09840 | mntH |
| H2O2_30min_vs_baseline | H2O2 | 30 | UP | LAR_RS09885 | metE2 |
| H2O2_30min_vs_baseline | H2O2 | 30 | UP | LAR_RS09890 |  |
| H2O2_30min_vs_baseline | H2O2 | 30 | UP | LAR_RS09895 |  |
| H2O2_30min_vs_baseline | H2O2 | 30 | UP | LAR_RS09910 |  |
| H2O2_30min_vs_baseline | H2O2 | 30 | UP | LAR_RS09940 | pepC |
| H2O2_30min_vs_baseline | H2O2 | 30 | UP | LAR_RS09955 | folT |
| H2O2_30min_vs_baseline | H2O2 | 30 | UP | LAR_RS10010 |  |
| H2O2_30min_vs_baseline | H2O2 | 30 | UP | LAR_RS10080 | pcaC |
| H2O2_30min_vs_baseline | H2O2 | 30 | UP | LAR_RS10100 |  |
| H2O2_30min_vs_baseline | H2O2 | 30 | UP | LAR_RS10150 | hprT |
| H2O2_30min_vs_baseline | H2O2 | 30 | UP | LAR_RS10155 | mgtC |
| H2O2_30min_vs_baseline | H2O2 | 30 | UP | LAR_RS10160 |  |
| H2O2_30min_vs_baseline | H2O2 | 30 | UP | LAR_RS10165 |  |
| H2O2_30min_vs_baseline | H2O2 | 30 | UP | LAR_RS10190 |  |
| H2O2_30min_vs_baseline | H2O2 | 30 | UP | LAR_RS10230 | jag |
| H2O2_30min_vs_baseline | H2O2 | 30 | DOWN | LAR_RS00080 |  |
| H2O2_30min_vs_baseline | H2O2 | 30 | DOWN | LAR_RS00090 |  |
| H2O2_30min_vs_baseline | H2O2 | 30 | DOWN | LAR_RS00095 |  |
| H2O2_30min_vs_baseline | H2O2 | 30 | DOWN | LAR_RS00105 | vicR |
| H2O2_30min_vs_baseline | H2O2 | 30 | DOWN | LAR_RS00165 | glcU |
| H2O2_30min_vs_baseline | H2O2 | 30 | DOWN | LAR_RS00220 | ldhA |
| H2O2_30min_vs_baseline | H2O2 | 30 | DOWN | LAR_RS00225 | patA |
| H2O2_30min_vs_baseline | H2O2 | 30 | DOWN | LAR_RS00315 | gshA |
| H2O2_30min_vs_baseline | H2O2 | 30 | DOWN | LAR_RS00405 |  |
| H2O2_30min_vs_baseline | H2O2 | 30 | DOWN | LAR_RS00435 | carB |
| H2O2_30min_vs_baseline | H2O2 | 30 | DOWN | LAR_RS00450 | malR3 |
| H2O2_30min_vs_baseline | H2O2 | 30 | DOWN | LAR_RS00455 | rumA |
| H2O2_30min_vs_baseline | H2O2 | 30 | DOWN | LAR_RS00480 |  |
| H2O2_30min_vs_baseline | H2O2 | 30 | DOWN | LAR_RS00490 | lsp |
| H2O2_30min_vs_baseline | H2O2 | 30 | DOWN | LAR_RS00520 | rihC |
| H2O2_30min_vs_baseline | H2O2 | 30 | DOWN | LAR_RS00525 | paaH |
| H2O2_30min_vs_baseline | H2O2 | 30 | DOWN | LAR_RS00565 | deoC |
| H2O2_30min_vs_baseline | H2O2 | 30 | DOWN | LAR_RS00570 | deoB |
| H2O2_30min_vs_baseline | H2O2 | 30 | DOWN | LAR_RS00575 | pdp |
| H2O2_30min_vs_baseline | H2O2 | 30 | DOWN | LAR_RS00580 | deoD |
| H2O2_30min_vs_baseline | H2O2 | 30 | DOWN | LAR_RS00590 | guaB |
| H2O2_30min_vs_baseline | H2O2 | 30 | DOWN | LAR_RS00740 |  |
| H2O2_30min_vs_baseline | H2O2 | 30 | DOWN | LAR_RS00745 | tyrS |
| H2O2_30min_vs_baseline | H2O2 | 30 | DOWN | LAR_RS00810 | pncB |
| H2O2_30min_vs_baseline | H2O2 | 30 | DOWN | LAR_RS00815 | pncA |
| H2O2_30min_vs_baseline | H2O2 | 30 | DOWN | LAR_RS00875 |  |
| H2O2_30min_vs_baseline | H2O2 | 30 | DOWN | LAR_RS00950 |  |
| H2O2_30min_vs_baseline | H2O2 | 30 | DOWN | LAR_RS00995 | metN |
| H2O2_30min_vs_baseline | H2O2 | 30 | DOWN | LAR_RS01000 | metQ |
| H2O2_30min_vs_baseline | H2O2 | 30 | DOWN | LAR_RS01015 |  |
| H2O2_30min_vs_baseline | H2O2 | 30 | DOWN | LAR_RS01020 |  |
| H2O2_30min_vs_baseline | H2O2 | 30 | DOWN | LAR_RS01090 | metG |
| H2O2_30min_vs_baseline | H2O2 | 30 | DOWN | LAR_RS01095 | tatD |
| H2O2_30min_vs_baseline | H2O2 | 30 | DOWN | LAR_RS01100 | rnmV |
| H2O2_30min_vs_baseline | H2O2 | 30 | DOWN | LAR_RS01105 | ksgA |
| H2O2_30min_vs_baseline | H2O2 | 30 | DOWN | LAR_RS01135 | purR |
| H2O2_30min_vs_baseline | H2O2 | 30 | DOWN | LAR_RS01140 | glmU |
| H2O2_30min_vs_baseline | H2O2 | 30 | DOWN | LAR_RS01185 | rpoE |
| H2O2_30min_vs_baseline | H2O2 | 30 | DOWN | LAR_RS01245 | lemA |
| H2O2_30min_vs_baseline | H2O2 | 30 | DOWN | LAR_RS01250 | htpX |
| H2O2_30min_vs_baseline | H2O2 | 30 | DOWN | LAR_RS01255 | murF |
| H2O2_30min_vs_baseline | H2O2 | 30 | DOWN | LAR_RS01260 | deaD |
| H2O2_30min_vs_baseline | H2O2 | 30 | DOWN | LAR_RS01335 | hpaH |
| H2O2_30min_vs_baseline | H2O2 | 30 | DOWN | LAR_RS01400 |  |
| H2O2_30min_vs_baseline | H2O2 | 30 | DOWN | LAR_RS01500 | pncB |
| H2O2_30min_vs_baseline | H2O2 | 30 | DOWN | LAR_RS01505 | nadE |
| H2O2_30min_vs_baseline | H2O2 | 30 | DOWN | LAR_RS01510 |  |
| H2O2_30min_vs_baseline | H2O2 | 30 | DOWN | LAR_RS01515 | sprL |
| H2O2_30min_vs_baseline | H2O2 | 30 | DOWN | LAR_RS01565 | cyuC |
| H2O2_30min_vs_baseline | H2O2 | 30 | DOWN | LAR_RS01620 | nusG |
| H2O2_30min_vs_baseline | H2O2 | 30 | DOWN | LAR_RS01640 |  |
| H2O2_30min_vs_baseline | H2O2 | 30 | DOWN | LAR_RS01675 | ywzG |
| H2O2_30min_vs_baseline | H2O2 | 30 | DOWN | LAR_RS01680 | lp_3217 |
| H2O2_30min_vs_baseline | H2O2 | 30 | DOWN | LAR_RS01695 | nrdF |
| H2O2_30min_vs_baseline | H2O2 | 30 | DOWN | LAR_RS01700 | nrdE |
| H2O2_30min_vs_baseline | H2O2 | 30 | DOWN | LAR_RS01705 | nrdH |
| H2O2_30min_vs_baseline | H2O2 | 30 | DOWN | LAR_RS01785 |  |
| H2O2_30min_vs_baseline | H2O2 | 30 | DOWN | LAR_RS01790 |  |
| H2O2_30min_vs_baseline | H2O2 | 30 | DOWN | LAR_RS01900 |  |
| H2O2_30min_vs_baseline | H2O2 | 30 | DOWN | LAR_RS02100 | rnr |
| H2O2_30min_vs_baseline | H2O2 | 30 | DOWN | LAR_RS02105 | smpB |
| H2O2_30min_vs_baseline | H2O2 | 30 | DOWN | LAR_RS02140 | dnaQ |
| H2O2_30min_vs_baseline | H2O2 | 30 | DOWN | LAR_RS02150 | rbsK |
| H2O2_30min_vs_baseline | H2O2 | 30 | DOWN | LAR_RS02155 | rbsD |
| H2O2_30min_vs_baseline | H2O2 | 30 | DOWN | LAR_RS02160 | rbsP |
| H2O2_30min_vs_baseline | H2O2 | 30 | DOWN | LAR_RS02210 |  |
| H2O2_30min_vs_baseline | H2O2 | 30 | DOWN | LAR_RS02215 |  |
| H2O2_30min_vs_baseline | H2O2 | 30 | DOWN | LAR_RS02225 | maa |
| H2O2_30min_vs_baseline | H2O2 | 30 | DOWN | LAR_RS02460 | pyrP |
| H2O2_30min_vs_baseline | H2O2 | 30 | DOWN | LAR_RS02535 |  |
| H2O2_30min_vs_baseline | H2O2 | 30 | DOWN | LAR_RS02560 | araD |
| H2O2_30min_vs_baseline | H2O2 | 30 | DOWN | LAR_RS02565 | araA |
| H2O2_30min_vs_baseline | H2O2 | 30 | DOWN | LAR_RS02575 |  |
| H2O2_30min_vs_baseline | H2O2 | 30 | DOWN | LAR_RS02580 | araR |
| H2O2_30min_vs_baseline | H2O2 | 30 | DOWN | LAR_RS02610 |  |
| H2O2_30min_vs_baseline | H2O2 | 30 | DOWN | LAR_RS02615 |  |
| H2O2_30min_vs_baseline | H2O2 | 30 | DOWN | LAR_RS02675 | valS |
| H2O2_30min_vs_baseline | H2O2 | 30 | DOWN | LAR_RS02730 |  |
| H2O2_30min_vs_baseline | H2O2 | 30 | DOWN | LAR_RS02735 |  |
| H2O2_30min_vs_baseline | H2O2 | 30 | DOWN | LAR_RS02740 |  |
| H2O2_30min_vs_baseline | H2O2 | 30 | DOWN | LAR_RS02900 | pepQ |
| H2O2_30min_vs_baseline | H2O2 | 30 | DOWN | LAR_RS03030 | NA |
| H2O2_30min_vs_baseline | H2O2 | 30 | DOWN | LAR_RS03035 | NA |
| H2O2_30min_vs_baseline | H2O2 | 30 | DOWN | LAR_RS03050 | NA |
| H2O2_30min_vs_baseline | H2O2 | 30 | DOWN | LAR_RS03055 | NA |
| H2O2_30min_vs_baseline | H2O2 | 30 | DOWN | LAR_RS03060 | NA |
| H2O2_30min_vs_baseline | H2O2 | 30 | DOWN | LAR_RS03065 | NA |
| H2O2_30min_vs_baseline | H2O2 | 30 | DOWN | LAR_RS03070 | NA |
| H2O2_30min_vs_baseline | H2O2 | 30 | DOWN | LAR_RS03075 | NA |
| H2O2_30min_vs_baseline | H2O2 | 30 | DOWN | LAR_RS03080 | NA |
| H2O2_30min_vs_baseline | H2O2 | 30 | DOWN | LAR_RS03085 | NA |
| H2O2_30min_vs_baseline | H2O2 | 30 | DOWN | LAR_RS03090 | NA |
| H2O2_30min_vs_baseline | H2O2 | 30 | DOWN | LAR_RS03095 |  |
| H2O2_30min_vs_baseline | H2O2 | 30 | DOWN | LAR_RS03170 |  |
| H2O2_30min_vs_baseline | H2O2 | 30 | DOWN | LAR_RS03175 |  |
| H2O2_30min_vs_baseline | H2O2 | 30 | DOWN | LAR_RS03180 | pgl |
| H2O2_30min_vs_baseline | H2O2 | 30 | DOWN | LAR_RS03275 | ileS |
| H2O2_30min_vs_baseline | H2O2 | 30 | DOWN | LAR_RS03340 | lysC |
| H2O2_30min_vs_baseline | H2O2 | 30 | DOWN | LAR_RS03345 | lysA |
| H2O2_30min_vs_baseline | H2O2 | 30 | DOWN | LAR_RS03350 | dapD |
| H2O2_30min_vs_baseline | H2O2 | 30 | DOWN | LAR_RS03355 | dapL |
| H2O2_30min_vs_baseline | H2O2 | 30 | DOWN | LAR_RS03360 | dapA |
| H2O2_30min_vs_baseline | H2O2 | 30 | DOWN | LAR_RS03370 | patA |
| H2O2_30min_vs_baseline | H2O2 | 30 | DOWN | LAR_RS03385 |  |
| H2O2_30min_vs_baseline | H2O2 | 30 | DOWN | LAR_RS03450 | pdhC |
| H2O2_30min_vs_baseline | H2O2 | 30 | DOWN | LAR_RS03455 | pdhD |
| H2O2_30min_vs_baseline | H2O2 | 30 | DOWN | LAR_RS03505 | comEB |
| H2O2_30min_vs_baseline | H2O2 | 30 | DOWN | LAR_RS03530 |  |
| H2O2_30min_vs_baseline | H2O2 | 30 | DOWN | LAR_RS03535 |  |
| H2O2_30min_vs_baseline | H2O2 | 30 | DOWN | LAR_RS03625 |  |
| H2O2_30min_vs_baseline | H2O2 | 30 | DOWN | LAR_RS03630 |  |
| H2O2_30min_vs_baseline | H2O2 | 30 | DOWN | LAR_RS03645 |  |
| H2O2_30min_vs_baseline | H2O2 | 30 | DOWN | LAR_RS03765 | rimP |
| H2O2_30min_vs_baseline | H2O2 | 30 | DOWN | LAR_RS03770 | nusA |
| H2O2_30min_vs_baseline | H2O2 | 30 | DOWN | LAR_RS03775 |  |
| H2O2_30min_vs_baseline | H2O2 | 30 | DOWN | LAR_RS03780 |  |
| H2O2_30min_vs_baseline | H2O2 | 30 | DOWN | LAR_RS03880 |  |
| H2O2_30min_vs_baseline | H2O2 | 30 | DOWN | LAR_RS04020 | patA |
| H2O2_30min_vs_baseline | H2O2 | 30 | DOWN | LAR_RS04025 |  |
| H2O2_30min_vs_baseline | H2O2 | 30 | DOWN | LAR_RS04660 | ribD |
| H2O2_30min_vs_baseline | H2O2 | 30 | DOWN | LAR_RS04665 | ribE |
| H2O2_30min_vs_baseline | H2O2 | 30 | DOWN | LAR_RS04670 | ribBA |
| H2O2_30min_vs_baseline | H2O2 | 30 | DOWN | LAR_RS04675 | ribH |
| H2O2_30min_vs_baseline | H2O2 | 30 | DOWN | LAR_RS04680 | kch |
| H2O2_30min_vs_baseline | H2O2 | 30 | DOWN | LAR_RS04685 | yfhO |
| H2O2_30min_vs_baseline | H2O2 | 30 | DOWN | LAR_RS04695 | sigH |
| H2O2_30min_vs_baseline | H2O2 | 30 | DOWN | LAR_RS04765 |  |
| H2O2_30min_vs_baseline | H2O2 | 30 | DOWN | LAR_RS04820 | melA |
| H2O2_30min_vs_baseline | H2O2 | 30 | DOWN | LAR_RS04970 |  |
| H2O2_30min_vs_baseline | H2O2 | 30 | DOWN | LAR_RS05090 |  |
| H2O2_30min_vs_baseline | H2O2 | 30 | DOWN | LAR_RS05160 | frnE |
| H2O2_30min_vs_baseline | H2O2 | 30 | DOWN | LAR_RS05180 | NA |
| H2O2_30min_vs_baseline | H2O2 | 30 | DOWN | LAR_RS05255 | acpP |
| H2O2_30min_vs_baseline | H2O2 | 30 | DOWN | LAR_RS05260 | fabH |
| H2O2_30min_vs_baseline | H2O2 | 30 | DOWN | LAR_RS05265 | fabT |
| H2O2_30min_vs_baseline | H2O2 | 30 | DOWN | LAR_RS05270 | fabA |
| H2O2_30min_vs_baseline | H2O2 | 30 | DOWN | LAR_RS05280 | xerS |
| H2O2_30min_vs_baseline | H2O2 | 30 | DOWN | LAR_RS05290 |  |
| H2O2_30min_vs_baseline | H2O2 | 30 | DOWN | LAR_RS05295 |  |
| H2O2_30min_vs_baseline | H2O2 | 30 | DOWN | LAR_RS05465 |  |
| H2O2_30min_vs_baseline | H2O2 | 30 | DOWN | LAR_RS05470 |  |
| H2O2_30min_vs_baseline | H2O2 | 30 | DOWN | LAR_RS05475 |  |
| H2O2_30min_vs_baseline | H2O2 | 30 | DOWN | LAR_RS05485 |  |
| H2O2_30min_vs_baseline | H2O2 | 30 | DOWN | LAR_RS05490 |  |
| H2O2_30min_vs_baseline | H2O2 | 30 | DOWN | LAR_RS05590 | yhcR |
| H2O2_30min_vs_baseline | H2O2 | 30 | DOWN | LAR_RS05700 |  |
| H2O2_30min_vs_baseline | H2O2 | 30 | DOWN | LAR_RS05705 | rfbD |
| H2O2_30min_vs_baseline | H2O2 | 30 | DOWN | LAR_RS05730 |  |
| H2O2_30min_vs_baseline | H2O2 | 30 | DOWN | LAR_RS05740 |  |
| H2O2_30min_vs_baseline | H2O2 | 30 | DOWN | LAR_RS05745 |  |
| H2O2_30min_vs_baseline | H2O2 | 30 | DOWN | LAR_RS05780 | pepDA |
| H2O2_30min_vs_baseline | H2O2 | 30 | DOWN | LAR_RS05790 |  |
| H2O2_30min_vs_baseline | H2O2 | 30 | DOWN | LAR_RS05820 |  |
| H2O2_30min_vs_baseline | H2O2 | 30 | DOWN | LAR_RS05850 | mdh |
| H2O2_30min_vs_baseline | H2O2 | 30 | DOWN | LAR_RS05855 | menA |
| H2O2_30min_vs_baseline | H2O2 | 30 | DOWN | LAR_RS05860 | hepST |
| H2O2_30min_vs_baseline | H2O2 | 30 | DOWN | LAR_RS05875 | lacI |
| H2O2_30min_vs_baseline | H2O2 | 30 | DOWN | LAR_RS06230 | ffh |
| H2O2_30min_vs_baseline | H2O2 | 30 | DOWN | LAR_RS06235 |  |
| H2O2_30min_vs_baseline | H2O2 | 30 | DOWN | LAR_RS06240 | ftsY |
| H2O2_30min_vs_baseline | H2O2 | 30 | DOWN | LAR_RS06245 | smc |
| H2O2_30min_vs_baseline | H2O2 | 30 | DOWN | LAR_RS06250 | rnc |
| H2O2_30min_vs_baseline | H2O2 | 30 | DOWN | LAR_RS06330 | rpoZ |
| H2O2_30min_vs_baseline | H2O2 | 30 | DOWN | LAR_RS06335 | gmk |
| H2O2_30min_vs_baseline | H2O2 | 30 | DOWN | LAR_RS06525 | hemY |
| H2O2_30min_vs_baseline | H2O2 | 30 | DOWN | LAR_RS06540 |  |
| H2O2_30min_vs_baseline | H2O2 | 30 | DOWN | LAR_RS06570 | acyP |
| H2O2_30min_vs_baseline | H2O2 | 30 | DOWN | LAR_RS06575 | spoIIIJ |
| H2O2_30min_vs_baseline | H2O2 | 30 | DOWN | LAR_RS06580 | def |
| H2O2_30min_vs_baseline | H2O2 | 30 | DOWN | LAR_RS06585 | matE |
| H2O2_30min_vs_baseline | H2O2 | 30 | DOWN | LAR_RS06620 |  |
| H2O2_30min_vs_baseline | H2O2 | 30 | DOWN | LAR_RS06625 | rsfS |
| H2O2_30min_vs_baseline | H2O2 | 30 | DOWN | LAR_RS06630 | yqeK |
| H2O2_30min_vs_baseline | H2O2 | 30 | DOWN | LAR_RS06635 | nadD |
| H2O2_30min_vs_baseline | H2O2 | 30 | DOWN | LAR_RS06665 | infC |
| H2O2_30min_vs_baseline | H2O2 | 30 | DOWN | LAR_RS06775 | hit |
| H2O2_30min_vs_baseline | H2O2 | 30 | DOWN | LAR_RS06780 |  |
| H2O2_30min_vs_baseline | H2O2 | 30 | DOWN | LAR_RS06805 | pbp2A |
| H2O2_30min_vs_baseline | H2O2 | 30 | DOWN | LAR_RS06830 |  |
| H2O2_30min_vs_baseline | H2O2 | 30 | DOWN | LAR_RS06845 |  |
| H2O2_30min_vs_baseline | H2O2 | 30 | DOWN | LAR_RS06850 | folC |
| H2O2_30min_vs_baseline | H2O2 | 30 | DOWN | LAR_RS06855 | folE |
| H2O2_30min_vs_baseline | H2O2 | 30 | DOWN | LAR_RS06860 | folK |
| H2O2_30min_vs_baseline | H2O2 | 30 | DOWN | LAR_RS06865 | folB |
| H2O2_30min_vs_baseline | H2O2 | 30 | DOWN | LAR_RS06885 |  |
| H2O2_30min_vs_baseline | H2O2 | 30 | DOWN | LAR_RS06890 |  |
| H2O2_30min_vs_baseline | H2O2 | 30 | DOWN | LAR_RS06955 | tag |
| H2O2_30min_vs_baseline | H2O2 | 30 | DOWN | LAR_RS07065 | NA |
| H2O2_30min_vs_baseline | H2O2 | 30 | DOWN | LAR_RS07070 | NA |
| H2O2_30min_vs_baseline | H2O2 | 30 | DOWN | LAR_RS07075 | NA |
| H2O2_30min_vs_baseline | H2O2 | 30 | DOWN | LAR_RS07080 | NA |
| H2O2_30min_vs_baseline | H2O2 | 30 | DOWN | LAR_RS07085 | NA |
| H2O2_30min_vs_baseline | H2O2 | 30 | DOWN | LAR_RS07090 | NA |
| H2O2_30min_vs_baseline | H2O2 | 30 | DOWN | LAR_RS07130 | lafC |
| H2O2_30min_vs_baseline | H2O2 | 30 | DOWN | LAR_RS07135 | cpoA |
| H2O2_30min_vs_baseline | H2O2 | 30 | DOWN | LAR_RS07140 | lafA |
| H2O2_30min_vs_baseline | H2O2 | 30 | DOWN | LAR_RS07165 | panT |
| H2O2_30min_vs_baseline | H2O2 | 30 | DOWN | LAR_RS07180 | lp_2743 |
| H2O2_30min_vs_baseline | H2O2 | 30 | DOWN | LAR_RS07185 | lp_2742 |
| H2O2_30min_vs_baseline | H2O2 | 30 | DOWN | LAR_RS07200 |  |
| H2O2_30min_vs_baseline | H2O2 | 30 | DOWN | LAR_RS07235 |  |
| H2O2_30min_vs_baseline | H2O2 | 30 | DOWN | LAR_RS07240 |  |
| H2O2_30min_vs_baseline | H2O2 | 30 | DOWN | LAR_RS07295 |  |
| H2O2_30min_vs_baseline | H2O2 | 30 | DOWN | LAR_RS07315 | istB |
| H2O2_30min_vs_baseline | H2O2 | 30 | DOWN | LAR_RS07320 |  |
| H2O2_30min_vs_baseline | H2O2 | 30 | DOWN | LAR_RS07405 | recX |
| H2O2_30min_vs_baseline | H2O2 | 30 | DOWN | LAR_RS07415 |  |
| H2O2_30min_vs_baseline | H2O2 | 30 | DOWN | LAR_RS07420 |  |
| H2O2_30min_vs_baseline | H2O2 | 30 | DOWN | LAR_RS07425 |  |
| H2O2_30min_vs_baseline | H2O2 | 30 | DOWN | LAR_RS07610 | cylB |
| H2O2_30min_vs_baseline | H2O2 | 30 | DOWN | LAR_RS07615 |  |
| H2O2_30min_vs_baseline | H2O2 | 30 | DOWN | LAR_RS07620 |  |
| H2O2_30min_vs_baseline | H2O2 | 30 | DOWN | LAR_RS07775 | truA |
| H2O2_30min_vs_baseline | H2O2 | 30 | DOWN | LAR_RS07780 | cbiQ |
| H2O2_30min_vs_baseline | H2O2 | 30 | DOWN | LAR_RS07785 | cbiO |
| H2O2_30min_vs_baseline | H2O2 | 30 | DOWN | LAR_RS07790 | cbiO |
| H2O2_30min_vs_baseline | H2O2 | 30 | DOWN | LAR_RS07945 | fusA |
| H2O2_30min_vs_baseline | H2O2 | 30 | DOWN | LAR_RS07980 |  |
| H2O2_30min_vs_baseline | H2O2 | 30 | DOWN | LAR_RS08005 | NA |
| H2O2_30min_vs_baseline | H2O2 | 30 | DOWN | LAR_RS08015 | NA |
| H2O2_30min_vs_baseline | H2O2 | 30 | DOWN | LAR_RS08020 | NA |
| H2O2_30min_vs_baseline | H2O2 | 30 | DOWN | LAR_RS08030 | dnk |
| H2O2_30min_vs_baseline | H2O2 | 30 | DOWN | LAR_RS08040 | lysP2 |
| H2O2_30min_vs_baseline | H2O2 | 30 | DOWN | LAR_RS08065 | cspA |
| H2O2_30min_vs_baseline | H2O2 | 30 | DOWN | LAR_RS08095 | glnQ |
| H2O2_30min_vs_baseline | H2O2 | 30 | DOWN | LAR_RS08100 | glnH |
| H2O2_30min_vs_baseline | H2O2 | 30 | DOWN | LAR_RS08105 | glnP |
| H2O2_30min_vs_baseline | H2O2 | 30 | DOWN | LAR_RS08110 | glnP |
| H2O2_30min_vs_baseline | H2O2 | 30 | DOWN | LAR_RS08120 | rnj |
| H2O2_30min_vs_baseline | H2O2 | 30 | DOWN | LAR_RS08145 | rluD |
| H2O2_30min_vs_baseline | H2O2 | 30 | DOWN | LAR_RS08150 | NA |
| H2O2_30min_vs_baseline | H2O2 | 30 | DOWN | LAR_RS08270 | arcD |
| H2O2_30min_vs_baseline | H2O2 | 30 | DOWN | LAR_RS08300 | cysK |
| H2O2_30min_vs_baseline | H2O2 | 30 | DOWN | LAR_RS08405 |  |
| H2O2_30min_vs_baseline | H2O2 | 30 | DOWN | LAR_RS08455 | metQ |
| H2O2_30min_vs_baseline | H2O2 | 30 | DOWN | LAR_RS08460 | luxS |
| H2O2_30min_vs_baseline | H2O2 | 30 | DOWN | LAR_RS08485 |  |
| H2O2_30min_vs_baseline | H2O2 | 30 | DOWN | LAR_RS08490 |  |
| H2O2_30min_vs_baseline | H2O2 | 30 | DOWN | LAR_RS08495 |  |
| H2O2_30min_vs_baseline | H2O2 | 30 | DOWN | LAR_RS08500 |  |
| H2O2_30min_vs_baseline | H2O2 | 30 | DOWN | LAR_RS08610 | guaA |
| H2O2_30min_vs_baseline | H2O2 | 30 | DOWN | LAR_RS08615 |  |
| H2O2_30min_vs_baseline | H2O2 | 30 | DOWN | LAR_RS08635 |  |
| H2O2_30min_vs_baseline | H2O2 | 30 | DOWN | LAR_RS08740 | potD |
| H2O2_30min_vs_baseline | H2O2 | 30 | DOWN | LAR_RS08765 | pip |
| H2O2_30min_vs_baseline | H2O2 | 30 | DOWN | LAR_RS08770 |  |
| H2O2_30min_vs_baseline | H2O2 | 30 | DOWN | LAR_RS08775 | dps |
| H2O2_30min_vs_baseline | H2O2 | 30 | DOWN | LAR_RS08800 |  |
| H2O2_30min_vs_baseline | H2O2 | 30 | DOWN | LAR_RS08805 |  |
| H2O2_30min_vs_baseline | H2O2 | 30 | DOWN | LAR_RS08815 | iunH |
| H2O2_30min_vs_baseline | H2O2 | 30 | DOWN | LAR_RS08940 |  |
| H2O2_30min_vs_baseline | H2O2 | 30 | DOWN | LAR_RS09150 | pduO |
| H2O2_30min_vs_baseline | H2O2 | 30 | DOWN | LAR_RS09160 | fldA |
| H2O2_30min_vs_baseline | H2O2 | 30 | DOWN | LAR_RS09170 |  |
| H2O2_30min_vs_baseline | H2O2 | 30 | DOWN | LAR_RS09175 | cobC |
| H2O2_30min_vs_baseline | H2O2 | 30 | DOWN | LAR_RS09350 | zwf |
| H2O2_30min_vs_baseline | H2O2 | 30 | DOWN | LAR_RS09455 | yxdJ |
| H2O2_30min_vs_baseline | H2O2 | 30 | DOWN | LAR_RS09460 |  |
| H2O2_30min_vs_baseline | H2O2 | 30 | DOWN | LAR_RS09475 | metA |
| H2O2_30min_vs_baseline | H2O2 | 30 | DOWN | LAR_RS09480 | cysK |
| H2O2_30min_vs_baseline | H2O2 | 30 | DOWN | LAR_RS09530 |  |
| H2O2_30min_vs_baseline | H2O2 | 30 | DOWN | LAR_RS09560 |  |
| H2O2_30min_vs_baseline | H2O2 | 30 | DOWN | LAR_RS09565 |  |
| H2O2_30min_vs_baseline | H2O2 | 30 | DOWN | LAR_RS09570 | brnQ |
| H2O2_30min_vs_baseline | H2O2 | 30 | DOWN | LAR_RS09590 |  |
| H2O2_30min_vs_baseline | H2O2 | 30 | DOWN | LAR_RS09615 |  |
| H2O2_30min_vs_baseline | H2O2 | 30 | DOWN | LAR_RS09620 |  |
| H2O2_30min_vs_baseline | H2O2 | 30 | DOWN | LAR_RS09625 |  |
| H2O2_30min_vs_baseline | H2O2 | 30 | DOWN | LAR_RS09630 |  |
| H2O2_30min_vs_baseline | H2O2 | 30 | DOWN | LAR_RS09640 | czcR1 |
| H2O2_30min_vs_baseline | H2O2 | 30 | DOWN | LAR_RS09660 | rluD |
| H2O2_30min_vs_baseline | H2O2 | 30 | DOWN | LAR_RS09680 | nhaC2 |
| H2O2_30min_vs_baseline | H2O2 | 30 | DOWN | LAR_RS09780 |  |
| H2O2_30min_vs_baseline | H2O2 | 30 | DOWN | LAR_RS09795 | scrR |
| H2O2_30min_vs_baseline | H2O2 | 30 | DOWN | LAR_RS09825 | yxeA |
| H2O2_30min_vs_baseline | H2O2 | 30 | DOWN | LAR_RS09830 | yxeR |
| H2O2_30min_vs_baseline | H2O2 | 30 | DOWN | LAR_RS09850 | thrS |
| H2O2_30min_vs_baseline | H2O2 | 30 | DOWN | LAR_RS09960 | asnA |
| H2O2_30min_vs_baseline | H2O2 | 30 | DOWN | LAR_RS10005 |  |
| H2O2_30min_vs_baseline | H2O2 | 30 | DOWN | LAR_RS10020 |  |
| H2O2_30min_vs_baseline | H2O2 | 30 | DOWN | LAR_RS10025 |  |
| H2O2_30min_vs_baseline | H2O2 | 30 | DOWN | LAR_RS10035 | ansA |
| H2O2_30min_vs_baseline | H2O2 | 30 | DOWN | LAR_RS10040 |  |
| H2O2_30min_vs_baseline | H2O2 | 30 | DOWN | LAR_RS10045 |  |
| H2O2_30min_vs_baseline | H2O2 | 30 | DOWN | LAR_RS10050 | hicD3 |
| H2O2_30min_vs_baseline | H2O2 | 30 | DOWN | LAR_RS10055 | hpaH |
| H2O2_30min_vs_baseline | H2O2 | 30 | DOWN | LAR_RS10110 |  |
| H2O2_30min_vs_baseline | H2O2 | 30 | DOWN | LAR_RS10135 | ybaK |
| H2O2_30min_vs_baseline | H2O2 | 30 | DOWN | LAR_RS10140 |  |
| H2O2_30min_vs_baseline | H2O2 | 30 | DOWN | LAR_RS10270 |  |
| H2O2_30min_vs_baseline | H2O2 | 30 | DOWN | LAR_RS10285 | NA |
| HOCl_5min_vs_baseline | HOCl | 5 | UP | LAR_RS00065 |  |
| HOCl_5min_vs_baseline | HOCl | 5 | UP | LAR_RS00070 |  |
| HOCl_5min_vs_baseline | HOCl | 5 | UP | LAR_RS00215 | osmC |
| HOCl_5min_vs_baseline | HOCl | 5 | UP | LAR_RS00275 | malT |
| HOCl_5min_vs_baseline | HOCl | 5 | UP | LAR_RS00280 | mapA |
| HOCl_5min_vs_baseline | HOCl | 5 | UP | LAR_RS00285 | pgmB |
| HOCl_5min_vs_baseline | HOCl | 5 | UP | LAR_RS00445 | malY |
| HOCl_5min_vs_baseline | HOCl | 5 | UP | LAR_RS00855 |  |
| HOCl_5min_vs_baseline | HOCl | 5 | UP | LAR_RS00910 |  |
| HOCl_5min_vs_baseline | HOCl | 5 | UP | LAR_RS00975 | msrB |
| HOCl_5min_vs_baseline | HOCl | 5 | UP | LAR_RS01345 | spoVC |
| HOCl_5min_vs_baseline | HOCl | 5 | UP | LAR_RS01690 | adhE |
| HOCl_5min_vs_baseline | HOCl | 5 | UP | LAR_RS01975 | trxB |
| HOCl_5min_vs_baseline | HOCl | 5 | UP | LAR_RS02010 | uvrB |
| HOCl_5min_vs_baseline | HOCl | 5 | UP | LAR_RS02280 | copA2 |
| HOCl_5min_vs_baseline | HOCl | 5 | UP | LAR_RS02285 | copA3 |
| HOCl_5min_vs_baseline | HOCl | 5 | UP | LAR_RS02290 | copA |
| HOCl_5min_vs_baseline | HOCl | 5 | UP | LAR_RS02605 |  |
| HOCl_5min_vs_baseline | HOCl | 5 | UP | LAR_RS02625 | oxc |
| HOCl_5min_vs_baseline | HOCl | 5 | UP | LAR_RS02850 | trxA |
| HOCl_5min_vs_baseline | HOCl | 5 | UP | LAR_RS03105 | spxA |
| HOCl_5min_vs_baseline | HOCl | 5 | UP | LAR_RS03125 |  |
| HOCl_5min_vs_baseline | HOCl | 5 | UP | LAR_RS03365 | dapB |
| HOCl_5min_vs_baseline | HOCl | 5 | UP | LAR_RS04130 |  |
| HOCl_5min_vs_baseline | HOCl | 5 | UP | LAR_RS04235 |  |
| HOCl_5min_vs_baseline | HOCl | 5 | UP | LAR_RS04795 | lrgA |
| HOCl_5min_vs_baseline | HOCl | 5 | UP | LAR_RS04800 | lrgB |
| HOCl_5min_vs_baseline | HOCl | 5 | UP | LAR_RS04805 | ldh |
| HOCl_5min_vs_baseline | HOCl | 5 | UP | LAR_RS05010 | copR |
| HOCl_5min_vs_baseline | HOCl | 5 | UP | LAR_RS05795 | ahpF |
| HOCl_5min_vs_baseline | HOCl | 5 | UP | LAR_RS05800 | ahpC |
| HOCl_5min_vs_baseline | HOCl | 5 | UP | LAR_RS05825 | rpsN |
| HOCl_5min_vs_baseline | HOCl | 5 | UP | LAR_RS05900 |  |
| HOCl_5min_vs_baseline | HOCl | 5 | UP | LAR_RS06340 |  |
| HOCl_5min_vs_baseline | HOCl | 5 | UP | LAR_RS06720 | trxD |
| HOCl_5min_vs_baseline | HOCl | 5 | UP | LAR_RS06970 | perR |
| HOCl_5min_vs_baseline | HOCl | 5 | UP | LAR_RS07000 | hsp20 |
| HOCl_5min_vs_baseline | HOCl | 5 | UP | LAR_RS07020 | xthA |
| HOCl_5min_vs_baseline | HOCl | 5 | UP | LAR_RS07160 | clpE |
| HOCl_5min_vs_baseline | HOCl | 5 | UP | LAR_RS07555 |  |
| HOCl_5min_vs_baseline | HOCl | 5 | UP | LAR_RS07560 |  |
| HOCl_5min_vs_baseline | HOCl | 5 | UP | LAR_RS07565 |  |
| HOCl_5min_vs_baseline | HOCl | 5 | UP | LAR_RS07570 | gor |
| HOCl_5min_vs_baseline | HOCl | 5 | UP | LAR_RS08080 | pcl1 |
| HOCl_5min_vs_baseline | HOCl | 5 | UP | LAR_RS08085 | pcl2 |
| HOCl_5min_vs_baseline | HOCl | 5 | UP | LAR_RS08465 |  |
| HOCl_5min_vs_baseline | HOCl | 5 | UP | LAR_RS08470 |  |
| HOCl_5min_vs_baseline | HOCl | 5 | UP | LAR_RS08735 |  |
| HOCl_5min_vs_baseline | HOCl | 5 | UP | LAR_RS08820 |  |
| HOCl_5min_vs_baseline | HOCl | 5 | UP | LAR_RS08880 | gatC |
| HOCl_5min_vs_baseline | HOCl | 5 | UP | LAR_RS08950 |  |
| HOCl_5min_vs_baseline | HOCl | 5 | UP | LAR_RS09475 | metA |
| HOCl_5min_vs_baseline | HOCl | 5 | UP | LAR_RS09480 | cysK |
| HOCl_5min_vs_baseline | HOCl | 5 | UP | LAR_RS09770 |  |
| HOCl_5min_vs_baseline | HOCl | 5 | UP | LAR_RS09920 |  |
| HOCl_5min_vs_baseline | HOCl | 5 | UP | LAR_RS09940 | pepC |
| HOCl_5min_vs_baseline | HOCl | 5 | UP | LAR_RS09945 |  |
| HOCl_5min_vs_baseline | HOCl | 5 | UP | LAR_RS09950 | pepN |
| HOCl_5min_vs_baseline | HOCl | 5 | UP | LAR_RS10190 |  |
| HOCl_5min_vs_baseline | HOCl | 5 | DOWN | LAR_RS00565 | deoC |
| HOCl_5min_vs_baseline | HOCl | 5 | DOWN | LAR_RS02005 |  |
| HOCl_5min_vs_baseline | HOCl | 5 | DOWN | LAR_RS02150 | rbsK |
| HOCl_5min_vs_baseline | HOCl | 5 | DOWN | LAR_RS02155 | rbsD |
| HOCl_5min_vs_baseline | HOCl | 5 | DOWN | LAR_RS02160 | rbsP |
| HOCl_5min_vs_baseline | HOCl | 5 | DOWN | LAR_RS02180 | glmS |
| HOCl_5min_vs_baseline | HOCl | 5 | DOWN | LAR_RS02650 | pbuG |
| HOCl_5min_vs_baseline | HOCl | 5 | DOWN | LAR_RS03040 | NA |
| HOCl_5min_vs_baseline | HOCl | 5 | DOWN | LAR_RS03050 | NA |
| HOCl_5min_vs_baseline | HOCl | 5 | DOWN | LAR_RS03055 | NA |
| HOCl_5min_vs_baseline | HOCl | 5 | DOWN | LAR_RS03070 | NA |
| HOCl_5min_vs_baseline | HOCl | 5 | DOWN | LAR_RS05335 | moeB |
| HOCl_5min_vs_baseline | HOCl | 5 | DOWN | LAR_RS05395 | moaD |
| HOCl_5min_vs_baseline | HOCl | 5 | DOWN | LAR_RS05455 |  |
| HOCl_5min_vs_baseline | HOCl | 5 | DOWN | LAR_RS05460 |  |
| HOCl_5min_vs_baseline | HOCl | 5 | DOWN | LAR_RS08030 | dnk |
| HOCl_5min_vs_baseline | HOCl | 5 | DOWN | LAR_RS08960 |  |
| HOCl_5min_vs_baseline | HOCl | 5 | DOWN | LAR_RS09365 | lacS |
| HOCl_5min_vs_baseline | HOCl | 5 | DOWN | LAR_RS10150 | hprT |
| HOCl_15min_vs_baseline | HOCl | 15 | UP | LAR_RS00065 |  |
| HOCl_15min_vs_baseline | HOCl | 15 | UP | LAR_RS00070 |  |
| HOCl_15min_vs_baseline | HOCl | 15 | UP | LAR_RS00215 | osmC |
| HOCl_15min_vs_baseline | HOCl | 15 | UP | LAR_RS00270 | atoB |
| HOCl_15min_vs_baseline | HOCl | 15 | UP | LAR_RS00275 | malT |
| HOCl_15min_vs_baseline | HOCl | 15 | UP | LAR_RS00280 | mapA |
| HOCl_15min_vs_baseline | HOCl | 15 | UP | LAR_RS00285 | pgmB |
| HOCl_15min_vs_baseline | HOCl | 15 | UP | LAR_RS00290 |  |
| HOCl_15min_vs_baseline | HOCl | 15 | UP | LAR_RS00445 | malY |
| HOCl_15min_vs_baseline | HOCl | 15 | UP | LAR_RS00805 |  |
| HOCl_15min_vs_baseline | HOCl | 15 | UP | LAR_RS00855 |  |
| HOCl_15min_vs_baseline | HOCl | 15 | UP | LAR_RS00865 | nhaC |
| HOCl_15min_vs_baseline | HOCl | 15 | UP | LAR_RS00910 |  |
| HOCl_15min_vs_baseline | HOCl | 15 | UP | LAR_RS00915 | rclA |
| HOCl_15min_vs_baseline | HOCl | 15 | UP | LAR_RS00925 |  |
| HOCl_15min_vs_baseline | HOCl | 15 | UP | LAR_RS00975 | msrB |
| HOCl_15min_vs_baseline | HOCl | 15 | UP | LAR_RS01345 | spoVC |
| HOCl_15min_vs_baseline | HOCl | 15 | UP | LAR_RS01350 | mfd |
| HOCl_15min_vs_baseline | HOCl | 15 | UP | LAR_RS01450 |  |
| HOCl_15min_vs_baseline | HOCl | 15 | UP | LAR_RS01460 |  |
| HOCl_15min_vs_baseline | HOCl | 15 | UP | LAR_RS01540 | gpmA |
| HOCl_15min_vs_baseline | HOCl | 15 | UP | LAR_RS01690 | adhE |
| HOCl_15min_vs_baseline | HOCl | 15 | UP | LAR_RS01860 | groS |
| HOCl_15min_vs_baseline | HOCl | 15 | UP | LAR_RS01865 | groL |
| HOCl_15min_vs_baseline | HOCl | 15 | UP | LAR_RS01870 | ybaK |
| HOCl_15min_vs_baseline | HOCl | 15 | UP | LAR_RS01975 | trxB |
| HOCl_15min_vs_baseline | HOCl | 15 | UP | LAR_RS01980 | dapE |
| HOCl_15min_vs_baseline | HOCl | 15 | UP | LAR_RS02010 | uvrB |
| HOCl_15min_vs_baseline | HOCl | 15 | UP | LAR_RS02015 | uvrA |
| HOCl_15min_vs_baseline | HOCl | 15 | UP | LAR_RS02040 | clpP |
| HOCl_15min_vs_baseline | HOCl | 15 | UP | LAR_RS02275 |  |
| HOCl_15min_vs_baseline | HOCl | 15 | UP | LAR_RS02280 | copA2 |
| HOCl_15min_vs_baseline | HOCl | 15 | UP | LAR_RS02285 | copA3 |
| HOCl_15min_vs_baseline | HOCl | 15 | UP | LAR_RS02290 | copA |
| HOCl_15min_vs_baseline | HOCl | 15 | UP | LAR_RS02410 | murE |
| HOCl_15min_vs_baseline | HOCl | 15 | UP | LAR_RS02605 |  |
| HOCl_15min_vs_baseline | HOCl | 15 | UP | LAR_RS02615 |  |
| HOCl_15min_vs_baseline | HOCl | 15 | UP | LAR_RS02625 | oxc |
| HOCl_15min_vs_baseline | HOCl | 15 | UP | LAR_RS02845 | mutS2 |
| HOCl_15min_vs_baseline | HOCl | 15 | UP | LAR_RS02850 | trxA |
| HOCl_15min_vs_baseline | HOCl | 15 | UP | LAR_RS03105 | spxA |
| HOCl_15min_vs_baseline | HOCl | 15 | UP | LAR_RS03115 | coiA |
| HOCl_15min_vs_baseline | HOCl | 15 | UP | LAR_RS03120 | pepDA |
| HOCl_15min_vs_baseline | HOCl | 15 | UP | LAR_RS03125 |  |
| HOCl_15min_vs_baseline | HOCl | 15 | UP | LAR_RS03565 | uvrC |
| HOCl_15min_vs_baseline | HOCl | 15 | UP | LAR_RS03800 | ribF |
| HOCl_15min_vs_baseline | HOCl | 15 | UP | LAR_RS03810 | hrcA |
| HOCl_15min_vs_baseline | HOCl | 15 | UP | LAR_RS03815 | grpE |
| HOCl_15min_vs_baseline | HOCl | 15 | UP | LAR_RS04130 |  |
| HOCl_15min_vs_baseline | HOCl | 15 | UP | LAR_RS04235 |  |
| HOCl_15min_vs_baseline | HOCl | 15 | UP | LAR_RS04795 | lrgA |
| HOCl_15min_vs_baseline | HOCl | 15 | UP | LAR_RS04800 | lrgB |
| HOCl_15min_vs_baseline | HOCl | 15 | UP | LAR_RS04805 | ldh |
| HOCl_15min_vs_baseline | HOCl | 15 | UP | LAR_RS04895 | ypsC |
| HOCl_15min_vs_baseline | HOCl | 15 | UP | LAR_RS05775 |  |
| HOCl_15min_vs_baseline | HOCl | 15 | UP | LAR_RS05795 | ahpF |
| HOCl_15min_vs_baseline | HOCl | 15 | UP | LAR_RS05800 | ahpC |
| HOCl_15min_vs_baseline | HOCl | 15 | UP | LAR_RS05825 | rpsN |
| HOCl_15min_vs_baseline | HOCl | 15 | UP | LAR_RS05900 |  |
| HOCl_15min_vs_baseline | HOCl | 15 | UP | LAR_RS06340 |  |
| HOCl_15min_vs_baseline | HOCl | 15 | UP | LAR_RS06720 | trxD |
| HOCl_15min_vs_baseline | HOCl | 15 | UP | LAR_RS06730 | glsA |
| HOCl_15min_vs_baseline | HOCl | 15 | UP | LAR_RS07000 | hsp20 |
| HOCl_15min_vs_baseline | HOCl | 15 | UP | LAR_RS07020 | xthA |
| HOCl_15min_vs_baseline | HOCl | 15 | UP | LAR_RS07035 |  |
| HOCl_15min_vs_baseline | HOCl | 15 | UP | LAR_RS07160 | clpE |
| HOCl_15min_vs_baseline | HOCl | 15 | UP | LAR_RS07515 |  |
| HOCl_15min_vs_baseline | HOCl | 15 | UP | LAR_RS07550 | ogt |
| HOCl_15min_vs_baseline | HOCl | 15 | UP | LAR_RS07555 |  |
| HOCl_15min_vs_baseline | HOCl | 15 | UP | LAR_RS07560 |  |
| HOCl_15min_vs_baseline | HOCl | 15 | UP | LAR_RS07565 |  |
| HOCl_15min_vs_baseline | HOCl | 15 | UP | LAR_RS07570 | gor |
| HOCl_15min_vs_baseline | HOCl | 15 | UP | LAR_RS08080 | pcl1 |
| HOCl_15min_vs_baseline | HOCl | 15 | UP | LAR_RS08085 | pcl2 |
| HOCl_15min_vs_baseline | HOCl | 15 | UP | LAR_RS08160 | thiJ |
| HOCl_15min_vs_baseline | HOCl | 15 | UP | LAR_RS08205 | mleS |
| HOCl_15min_vs_baseline | HOCl | 15 | UP | LAR_RS08215 | ppiB |
| HOCl_15min_vs_baseline | HOCl | 15 | UP | LAR_RS08240 |  |
| HOCl_15min_vs_baseline | HOCl | 15 | UP | LAR_RS08395 |  |
| HOCl_15min_vs_baseline | HOCl | 15 | UP | LAR_RS08420 |  |
| HOCl_15min_vs_baseline | HOCl | 15 | UP | LAR_RS08465 |  |
| HOCl_15min_vs_baseline | HOCl | 15 | UP | LAR_RS08470 |  |
| HOCl_15min_vs_baseline | HOCl | 15 | UP | LAR_RS08590 | nplT |
| HOCl_15min_vs_baseline | HOCl | 15 | UP | LAR_RS08595 | malT2 |
| HOCl_15min_vs_baseline | HOCl | 15 | UP | LAR_RS08735 |  |
| HOCl_15min_vs_baseline | HOCl | 15 | UP | LAR_RS08780 |  |
| HOCl_15min_vs_baseline | HOCl | 15 | UP | LAR_RS08820 |  |
| HOCl_15min_vs_baseline | HOCl | 15 | UP | LAR_RS08880 | gatC |
| HOCl_15min_vs_baseline | HOCl | 15 | UP | LAR_RS08950 |  |
| HOCl_15min_vs_baseline | HOCl | 15 | UP | LAR_RS09360 |  |
| HOCl_15min_vs_baseline | HOCl | 15 | UP | LAR_RS09475 | metA |
| HOCl_15min_vs_baseline | HOCl | 15 | UP | LAR_RS09480 | cysK |
| HOCl_15min_vs_baseline | HOCl | 15 | UP | LAR_RS09770 |  |
| HOCl_15min_vs_baseline | HOCl | 15 | UP | LAR_RS09885 | metE2 |
| HOCl_15min_vs_baseline | HOCl | 15 | UP | LAR_RS09890 |  |
| HOCl_15min_vs_baseline | HOCl | 15 | UP | LAR_RS09895 |  |
| HOCl_15min_vs_baseline | HOCl | 15 | UP | LAR_RS09910 |  |
| HOCl_15min_vs_baseline | HOCl | 15 | UP | LAR_RS09920 |  |
| HOCl_15min_vs_baseline | HOCl | 15 | UP | LAR_RS09925 |  |
| HOCl_15min_vs_baseline | HOCl | 15 | UP | LAR_RS09940 | pepC |
| HOCl_15min_vs_baseline | HOCl | 15 | UP | LAR_RS09945 |  |
| HOCl_15min_vs_baseline | HOCl | 15 | UP | LAR_RS09950 | pepN |
| HOCl_15min_vs_baseline | HOCl | 15 | UP | LAR_RS09955 | folT |
| HOCl_15min_vs_baseline | HOCl | 15 | UP | LAR_RS10155 | mgtC |
| HOCl_15min_vs_baseline | HOCl | 15 | UP | LAR_RS10160 |  |
| HOCl_15min_vs_baseline | HOCl | 15 | UP | LAR_RS10190 |  |
| HOCl_15min_vs_baseline | HOCl | 15 | UP | LAR_RS10225 | trmE |
| HOCl_15min_vs_baseline | HOCl | 15 | UP | LAR_RS10230 | jag |
| HOCl_15min_vs_baseline | HOCl | 15 | DOWN | LAR_RS00185 |  |
| HOCl_15min_vs_baseline | HOCl | 15 | DOWN | LAR_RS00190 | macB |
| HOCl_15min_vs_baseline | HOCl | 15 | DOWN | LAR_RS00565 | deoC |
| HOCl_15min_vs_baseline | HOCl | 15 | DOWN | LAR_RS00570 | deoB |
| HOCl_15min_vs_baseline | HOCl | 15 | DOWN | LAR_RS00575 | pdp |
| HOCl_15min_vs_baseline | HOCl | 15 | DOWN | LAR_RS00580 | deoD |
| HOCl_15min_vs_baseline | HOCl | 15 | DOWN | LAR_RS01225 | tpiA |
| HOCl_15min_vs_baseline | HOCl | 15 | DOWN | LAR_RS01230 | fbaA |
| HOCl_15min_vs_baseline | HOCl | 15 | DOWN | LAR_RS02150 | rbsK |
| HOCl_15min_vs_baseline | HOCl | 15 | DOWN | LAR_RS02155 | rbsD |
| HOCl_15min_vs_baseline | HOCl | 15 | DOWN | LAR_RS02160 | rbsP |
| HOCl_15min_vs_baseline | HOCl | 15 | DOWN | LAR_RS02180 | glmS |
| HOCl_15min_vs_baseline | HOCl | 15 | DOWN | LAR_RS02650 | pbuG |
| HOCl_15min_vs_baseline | HOCl | 15 | DOWN | LAR_RS02730 |  |
| HOCl_15min_vs_baseline | HOCl | 15 | DOWN | LAR_RS02735 |  |
| HOCl_15min_vs_baseline | HOCl | 15 | DOWN | LAR_RS03220 | ftsI |
| HOCl_15min_vs_baseline | HOCl | 15 | DOWN | LAR_RS03225 | mraY |
| HOCl_15min_vs_baseline | HOCl | 15 | DOWN | LAR_RS03230 | murD |
| HOCl_15min_vs_baseline | HOCl | 15 | DOWN | LAR_RS03235 | murG |
| HOCl_15min_vs_baseline | HOCl | 15 | DOWN | LAR_RS04685 | yfhO |
| HOCl_15min_vs_baseline | HOCl | 15 | DOWN | LAR_RS05300 |  |
| HOCl_15min_vs_baseline | HOCl | 15 | DOWN | LAR_RS05315 | narI |
| HOCl_15min_vs_baseline | HOCl | 15 | DOWN | LAR_RS05320 | narJ |
| HOCl_15min_vs_baseline | HOCl | 15 | DOWN | LAR_RS05325 | narH |
| HOCl_15min_vs_baseline | HOCl | 15 | DOWN | LAR_RS05330 | narG |
| HOCl_15min_vs_baseline | HOCl | 15 | DOWN | LAR_RS05335 | moeB |
| HOCl_15min_vs_baseline | HOCl | 15 | DOWN | LAR_RS05350 | mobB |
| HOCl_15min_vs_baseline | HOCl | 15 | DOWN | LAR_RS05360 |  |
| HOCl_15min_vs_baseline | HOCl | 15 | DOWN | LAR_RS05375 |  |
| HOCl_15min_vs_baseline | HOCl | 15 | DOWN | LAR_RS05380 |  |
| HOCl_15min_vs_baseline | HOCl | 15 | DOWN | LAR_RS05385 |  |
| HOCl_15min_vs_baseline | HOCl | 15 | DOWN | LAR_RS05390 | narK |
| HOCl_15min_vs_baseline | HOCl | 15 | DOWN | LAR_RS05395 | moaD |
| HOCl_15min_vs_baseline | HOCl | 15 | DOWN | LAR_RS05400 | moaE |
| HOCl_15min_vs_baseline | HOCl | 15 | DOWN | LAR_RS05455 |  |
| HOCl_15min_vs_baseline | HOCl | 15 | DOWN | LAR_RS05460 |  |
| HOCl_15min_vs_baseline | HOCl | 15 | DOWN | LAR_RS05465 |  |
| HOCl_15min_vs_baseline | HOCl | 15 | DOWN | LAR_RS06955 | tag |
| HOCl_15min_vs_baseline | HOCl | 15 | DOWN | LAR_RS07180 | lp_2743 |
| HOCl_15min_vs_baseline | HOCl | 15 | DOWN | LAR_RS07185 | lp_2742 |
| HOCl_15min_vs_baseline | HOCl | 15 | DOWN | LAR_RS08770 |  |
| HOCl_15min_vs_baseline | HOCl | 15 | DOWN | LAR_RS08960 |  |
| HOCl_15min_vs_baseline | HOCl | 15 | DOWN | LAR_RS09030 | hemB |
| HOCl_15min_vs_baseline | HOCl | 15 | DOWN | LAR_RS09035 | hemC |
| HOCl_15min_vs_baseline | HOCl | 15 | DOWN | LAR_RS09040 | hemA |
| HOCl_15min_vs_baseline | HOCl | 15 | DOWN | LAR_RS09045 | cysG |
| HOCl_15min_vs_baseline | HOCl | 15 | DOWN | LAR_RS09050 | cbiP |
| HOCl_15min_vs_baseline | HOCl | 15 | DOWN | LAR_RS09065 | cbiN |
| HOCl_15min_vs_baseline | HOCl | 15 | DOWN | LAR_RS09080 | cbiK |
| HOCl_15min_vs_baseline | HOCl | 15 | DOWN | LAR_RS09365 | lacS |
| HOCl_15min_vs_baseline | HOCl | 15 | DOWN | LAR_RS09980 |  |
| HOCl_30min_vs_baseline | HOCl | 30 | UP | LAR_RS00065 |  |
| HOCl_30min_vs_baseline | HOCl | 30 | UP | LAR_RS00070 |  |
| HOCl_30min_vs_baseline | HOCl | 30 | UP | LAR_RS00180 | gabD |
| HOCl_30min_vs_baseline | HOCl | 30 | UP | LAR_RS00215 | osmC |
| HOCl_30min_vs_baseline | HOCl | 30 | UP | LAR_RS00270 | atoB |
| HOCl_30min_vs_baseline | HOCl | 30 | UP | LAR_RS00275 | malT |
| HOCl_30min_vs_baseline | HOCl | 30 | UP | LAR_RS00280 | mapA |
| HOCl_30min_vs_baseline | HOCl | 30 | UP | LAR_RS00285 | pgmB |
| HOCl_30min_vs_baseline | HOCl | 30 | UP | LAR_RS00290 |  |
| HOCl_30min_vs_baseline | HOCl | 30 | UP | LAR_RS00445 | malY |
| HOCl_30min_vs_baseline | HOCl | 30 | UP | LAR_RS00805 |  |
| HOCl_30min_vs_baseline | HOCl | 30 | UP | LAR_RS00855 |  |
| HOCl_30min_vs_baseline | HOCl | 30 | UP | LAR_RS00865 | nhaC |
| HOCl_30min_vs_baseline | HOCl | 30 | UP | LAR_RS00910 |  |
| HOCl_30min_vs_baseline | HOCl | 30 | UP | LAR_RS00915 | rclA |
| HOCl_30min_vs_baseline | HOCl | 30 | UP | LAR_RS00975 | msrB |
| HOCl_30min_vs_baseline | HOCl | 30 | UP | LAR_RS01345 | spoVC |
| HOCl_30min_vs_baseline | HOCl | 30 | UP | LAR_RS01350 | mfd |
| HOCl_30min_vs_baseline | HOCl | 30 | UP | LAR_RS01450 |  |
| HOCl_30min_vs_baseline | HOCl | 30 | UP | LAR_RS01540 | gpmA |
| HOCl_30min_vs_baseline | HOCl | 30 | UP | LAR_RS01550 | cgl |
| HOCl_30min_vs_baseline | HOCl | 30 | UP | LAR_RS01555 | cyuA |
| HOCl_30min_vs_baseline | HOCl | 30 | UP | LAR_RS01560 | cyuB |
| HOCl_30min_vs_baseline | HOCl | 30 | UP | LAR_RS01565 | cyuC |
| HOCl_30min_vs_baseline | HOCl | 30 | UP | LAR_RS01690 | adhE |
| HOCl_30min_vs_baseline | HOCl | 30 | UP | LAR_RS01855 |  |
| HOCl_30min_vs_baseline | HOCl | 30 | UP | LAR_RS01860 | groS |
| HOCl_30min_vs_baseline | HOCl | 30 | UP | LAR_RS01865 | groL |
| HOCl_30min_vs_baseline | HOCl | 30 | UP | LAR_RS01870 | ybaK |
| HOCl_30min_vs_baseline | HOCl | 30 | UP | LAR_RS01975 | trxB |
| HOCl_30min_vs_baseline | HOCl | 30 | UP | LAR_RS01980 | dapE |
| HOCl_30min_vs_baseline | HOCl | 30 | UP | LAR_RS02010 | uvrB |
| HOCl_30min_vs_baseline | HOCl | 30 | UP | LAR_RS02015 | uvrA |
| HOCl_30min_vs_baseline | HOCl | 30 | UP | LAR_RS02040 | clpP |
| HOCl_30min_vs_baseline | HOCl | 30 | UP | LAR_RS02275 |  |
| HOCl_30min_vs_baseline | HOCl | 30 | UP | LAR_RS02280 | copA2 |
| HOCl_30min_vs_baseline | HOCl | 30 | UP | LAR_RS02285 | copA3 |
| HOCl_30min_vs_baseline | HOCl | 30 | UP | LAR_RS02290 | copA |
| HOCl_30min_vs_baseline | HOCl | 30 | UP | LAR_RS02605 |  |
| HOCl_30min_vs_baseline | HOCl | 30 | UP | LAR_RS02610 |  |
| HOCl_30min_vs_baseline | HOCl | 30 | UP | LAR_RS02615 |  |
| HOCl_30min_vs_baseline | HOCl | 30 | UP | LAR_RS02625 | oxc |
| HOCl_30min_vs_baseline | HOCl | 30 | UP | LAR_RS02845 | mutS2 |
| HOCl_30min_vs_baseline | HOCl | 30 | UP | LAR_RS02850 | trxA |
| HOCl_30min_vs_baseline | HOCl | 30 | UP | LAR_RS03105 | spxA |
| HOCl_30min_vs_baseline | HOCl | 30 | UP | LAR_RS03125 |  |
| HOCl_30min_vs_baseline | HOCl | 30 | UP | LAR_RS03810 | hrcA |
| HOCl_30min_vs_baseline | HOCl | 30 | UP | LAR_RS03815 | grpE |
| HOCl_30min_vs_baseline | HOCl | 30 | UP | LAR_RS03820 | dnaK |
| HOCl_30min_vs_baseline | HOCl | 30 | UP | LAR_RS03825 | dnaJ |
| HOCl_30min_vs_baseline | HOCl | 30 | UP | LAR_RS04130 |  |
| HOCl_30min_vs_baseline | HOCl | 30 | UP | LAR_RS04235 |  |
| HOCl_30min_vs_baseline | HOCl | 30 | UP | LAR_RS04580 | gntK |
| HOCl_30min_vs_baseline | HOCl | 30 | UP | LAR_RS04595 |  |
| HOCl_30min_vs_baseline | HOCl | 30 | UP | LAR_RS04800 | lrgB |
| HOCl_30min_vs_baseline | HOCl | 30 | UP | LAR_RS04805 | ldh |
| HOCl_30min_vs_baseline | HOCl | 30 | UP | LAR_RS04895 | ypsC |
| HOCl_30min_vs_baseline | HOCl | 30 | UP | LAR_RS05640 |  |
| HOCl_30min_vs_baseline | HOCl | 30 | UP | LAR_RS05775 |  |
| HOCl_30min_vs_baseline | HOCl | 30 | UP | LAR_RS05795 | ahpF |
| HOCl_30min_vs_baseline | HOCl | 30 | UP | LAR_RS05800 | ahpC |
| HOCl_30min_vs_baseline | HOCl | 30 | UP | LAR_RS05825 | rpsN |
| HOCl_30min_vs_baseline | HOCl | 30 | UP | LAR_RS05900 |  |
| HOCl_30min_vs_baseline | HOCl | 30 | UP | LAR_RS06590 | yjeM |
| HOCl_30min_vs_baseline | HOCl | 30 | UP | LAR_RS06720 | trxD |
| HOCl_30min_vs_baseline | HOCl | 30 | UP | LAR_RS06730 | glsA |
| HOCl_30min_vs_baseline | HOCl | 30 | UP | LAR_RS07000 | hsp20 |
| HOCl_30min_vs_baseline | HOCl | 30 | UP | LAR_RS07020 | xthA |
| HOCl_30min_vs_baseline | HOCl | 30 | UP | LAR_RS07025 |  |
| HOCl_30min_vs_baseline | HOCl | 30 | UP | LAR_RS07035 |  |
| HOCl_30min_vs_baseline | HOCl | 30 | UP | LAR_RS07160 | clpE |
| HOCl_30min_vs_baseline | HOCl | 30 | UP | LAR_RS07550 | ogt |
| HOCl_30min_vs_baseline | HOCl | 30 | UP | LAR_RS07555 |  |
| HOCl_30min_vs_baseline | HOCl | 30 | UP | LAR_RS07560 |  |
| HOCl_30min_vs_baseline | HOCl | 30 | UP | LAR_RS07565 |  |
| HOCl_30min_vs_baseline | HOCl | 30 | UP | LAR_RS07570 | gor |
| HOCl_30min_vs_baseline | HOCl | 30 | UP | LAR_RS08080 | pcl1 |
| HOCl_30min_vs_baseline | HOCl | 30 | UP | LAR_RS08195 | frdA |
| HOCl_30min_vs_baseline | HOCl | 30 | UP | LAR_RS08200 | fumC |
| HOCl_30min_vs_baseline | HOCl | 30 | UP | LAR_RS08205 | mleS |
| HOCl_30min_vs_baseline | HOCl | 30 | UP | LAR_RS08210 | mleR |
| HOCl_30min_vs_baseline | HOCl | 30 | UP | LAR_RS08215 | ppiB |
| HOCl_30min_vs_baseline | HOCl | 30 | UP | LAR_RS08240 |  |
| HOCl_30min_vs_baseline | HOCl | 30 | UP | LAR_RS08255 | scrP |
| HOCl_30min_vs_baseline | HOCl | 30 | UP | LAR_RS08260 | scrT |
| HOCl_30min_vs_baseline | HOCl | 30 | UP | LAR_RS08300 | cysK |
| HOCl_30min_vs_baseline | HOCl | 30 | UP | LAR_RS08465 |  |
| HOCl_30min_vs_baseline | HOCl | 30 | UP | LAR_RS08470 |  |
| HOCl_30min_vs_baseline | HOCl | 30 | UP | LAR_RS08590 | nplT |
| HOCl_30min_vs_baseline | HOCl | 30 | UP | LAR_RS08595 | malT2 |
| HOCl_30min_vs_baseline | HOCl | 30 | UP | LAR_RS08735 |  |
| HOCl_30min_vs_baseline | HOCl | 30 | UP | LAR_RS08780 |  |
| HOCl_30min_vs_baseline | HOCl | 30 | UP | LAR_RS08820 |  |
| HOCl_30min_vs_baseline | HOCl | 30 | UP | LAR_RS08880 | gatC |
| HOCl_30min_vs_baseline | HOCl | 30 | UP | LAR_RS08950 |  |
| HOCl_30min_vs_baseline | HOCl | 30 | UP | LAR_RS09360 |  |
| HOCl_30min_vs_baseline | HOCl | 30 | UP | LAR_RS09770 |  |
| HOCl_30min_vs_baseline | HOCl | 30 | UP | LAR_RS09835 | mtlD |
| HOCl_30min_vs_baseline | HOCl | 30 | UP | LAR_RS09885 | metE2 |
| HOCl_30min_vs_baseline | HOCl | 30 | UP | LAR_RS09910 |  |
| HOCl_30min_vs_baseline | HOCl | 30 | UP | LAR_RS09920 |  |
| HOCl_30min_vs_baseline | HOCl | 30 | UP | LAR_RS09940 | pepC |
| HOCl_30min_vs_baseline | HOCl | 30 | UP | LAR_RS09945 |  |
| HOCl_30min_vs_baseline | HOCl | 30 | UP | LAR_RS09950 | pepN |
| HOCl_30min_vs_baseline | HOCl | 30 | UP | LAR_RS09955 | folT |
| HOCl_30min_vs_baseline | HOCl | 30 | UP | LAR_RS10010 |  |
| HOCl_30min_vs_baseline | HOCl | 30 | UP | LAR_RS10190 |  |
| HOCl_30min_vs_baseline | HOCl | 30 | UP | LAR_RS10225 | trmE |
| HOCl_30min_vs_baseline | HOCl | 30 | UP | LAR_RS10230 | jag |
| HOCl_30min_vs_baseline | HOCl | 30 | DOWN | LAR_RS00015 |  |
| HOCl_30min_vs_baseline | HOCl | 30 | DOWN | LAR_RS00565 | deoC |
| HOCl_30min_vs_baseline | HOCl | 30 | DOWN | LAR_RS00570 | deoB |
| HOCl_30min_vs_baseline | HOCl | 30 | DOWN | LAR_RS00575 | pdp |
| HOCl_30min_vs_baseline | HOCl | 30 | DOWN | LAR_RS00580 | deoD |
| HOCl_30min_vs_baseline | HOCl | 30 | DOWN | LAR_RS01225 | tpiA |
| HOCl_30min_vs_baseline | HOCl | 30 | DOWN | LAR_RS01230 | fbaA |
| HOCl_30min_vs_baseline | HOCl | 30 | DOWN | LAR_RS01685 | lp_3218 |
| HOCl_30min_vs_baseline | HOCl | 30 | DOWN | LAR_RS02150 | rbsK |
| HOCl_30min_vs_baseline | HOCl | 30 | DOWN | LAR_RS02155 | rbsD |
| HOCl_30min_vs_baseline | HOCl | 30 | DOWN | LAR_RS02160 | rbsP |
| HOCl_30min_vs_baseline | HOCl | 30 | DOWN | LAR_RS04665 | ribE |
| HOCl_30min_vs_baseline | HOCl | 30 | DOWN | LAR_RS04670 | ribBA |
| HOCl_30min_vs_baseline | HOCl | 30 | DOWN | LAR_RS04675 | ribH |
| HOCl_30min_vs_baseline | HOCl | 30 | DOWN | LAR_RS04765 |  |
| HOCl_30min_vs_baseline | HOCl | 30 | DOWN | LAR_RS05185 | NA |
| HOCl_30min_vs_baseline | HOCl | 30 | DOWN | LAR_RS05300 |  |
| HOCl_30min_vs_baseline | HOCl | 30 | DOWN | LAR_RS05315 | narI |
| HOCl_30min_vs_baseline | HOCl | 30 | DOWN | LAR_RS05320 | narJ |
| HOCl_30min_vs_baseline | HOCl | 30 | DOWN | LAR_RS05325 | narH |
| HOCl_30min_vs_baseline | HOCl | 30 | DOWN | LAR_RS05330 | narG |
| HOCl_30min_vs_baseline | HOCl | 30 | DOWN | LAR_RS05335 | moeB |
| HOCl_30min_vs_baseline | HOCl | 30 | DOWN | LAR_RS05350 | mobB |
| HOCl_30min_vs_baseline | HOCl | 30 | DOWN | LAR_RS05360 |  |
| HOCl_30min_vs_baseline | HOCl | 30 | DOWN | LAR_RS05375 |  |
| HOCl_30min_vs_baseline | HOCl | 30 | DOWN | LAR_RS05380 |  |
| HOCl_30min_vs_baseline | HOCl | 30 | DOWN | LAR_RS05385 |  |
| HOCl_30min_vs_baseline | HOCl | 30 | DOWN | LAR_RS05390 | narK |
| HOCl_30min_vs_baseline | HOCl | 30 | DOWN | LAR_RS05395 | moaD |
| HOCl_30min_vs_baseline | HOCl | 30 | DOWN | LAR_RS05400 | moaE |
| HOCl_30min_vs_baseline | HOCl | 30 | DOWN | LAR_RS05455 |  |
| HOCl_30min_vs_baseline | HOCl | 30 | DOWN | LAR_RS05460 |  |
| HOCl_30min_vs_baseline | HOCl | 30 | DOWN | LAR_RS07175 | lp_2744 |
| HOCl_30min_vs_baseline | HOCl | 30 | DOWN | LAR_RS07180 | lp_2743 |
| HOCl_30min_vs_baseline | HOCl | 30 | DOWN | LAR_RS07185 | lp_2742 |
| HOCl_30min_vs_baseline | HOCl | 30 | DOWN | LAR_RS08040 | lysP2 |
| HOCl_30min_vs_baseline | HOCl | 30 | DOWN | LAR_RS08065 | cspA |
| HOCl_30min_vs_baseline | HOCl | 30 | DOWN | LAR_RS09150 | pduO |
| HOCl_30min_vs_baseline | HOCl | 30 | DOWN | LAR_RS09365 | lacS |
