# Supplementary material for: Complex Responses to Hydrogen Peroxide and Hypochlorous Acid by the Probiotic Bacterium Lactobacillus reuteri
Source: mSystems. 2019 Sep 3;4(5):e00453-19. doi: 10.1128/mSystems.00453-19 (PMC6722424; doi:10.1128/mSystems.00453-19)
Supplement: TABLE S3 [file mSystems.00453-19-st003.docx]

| **Gene** | **Primer sequence** |
| --- | --- |
| *ppk1* (LAR_RS01770) | CAA GAA GTT AGC ACG TTC TAA CAA CGG ATT GGC AGT ATC AGT TTA GGA TCC CAG TAC TCG GCC ATT AAA GTC AAT CCA ACT CAA TTC TCG |
| *ppk2* (LAR_RS00075) | AAT CTT CTT AAG ATC ATC AGC AGA ATC TTT TAC CTT AGT TGG TTA AAG CTT AAT ATC AAA GTC TTT TCC AGT GTA ACG ATA TTT CTT GGT |
| *rclA* (LAR_RS00915) | ATT GCT TGC CTT CCC TCC AAA CGG CTA ATT ATT GAA GCT GCT AGC TAA ACC TCA TTT GAA GAC GCA GTC GAT GGG AAA AAT GTG ATG ACA |
| *msrB* (LAR_RS00975) | CGG TTC ATC AAA TCG ATC ATA TTT ACC ACT ATA TGG CTT TTC CTA GGA TCC CAT TTG AGT CAC AGC ATA TTG CAT TGG CGT TAA TTT CTT |
| *hslO* (LAR_RS01385) | TCC AAT TAA CGT TCG ACC AAG AGC AGC TGA AGA AGC ACT CCA TTA CAT ATG AAT TTC GTG TGC CTT TTG AAC GAC TTG GGT AGC ATT TAC |
| *lo18* (LAR_RS07000 | TAG GTT AGA GAA GAA ATC ATC GTT CAT CCA GTT ACG CAT GTT CTA AGT ACT ATC AAA TAA ATT ATT ACG GTT TTG TAA TTC ATT AGC CAT |
| LAR_RS09945 | TTA GTT GCT ACT GGT AAT GAA GTT ACA GCT GGT GCT CGC CAT ATG TGA AAA GTT ATT AAG TTA AAC CAC GTT ACT GCT GTA GAA TTG AAC |
| *sigH* (LAR_RS04695) | CGC GGA AGC AAT AAG GAC TTC GAA GAG TTA TTT CGC CGT TAT TAA TAA CTC GTT CGC CGA CTG TGG CAA CGA TAT AAT ATT AGC GGC CTT |
| *perR* (LAR_RS06970) | GAT TAT AAT TAT TTT AAG AAA GGG GAC GAC ATT ATG GCA GAA TTC TGA TTT GAT CGA GCT CTT GAT CAT CTC CGT GAA AAC AAG GTC CGT |
