# Supplementary material for: Complex Responses to Hydrogen Peroxide and Hypochlorous Acid by the Probiotic Bacterium Lactobacillus reuteri
Source: mSystems. 2019 Sep 3;4(5):e00453-19. doi: 10.1128/mSystems.00453-19 (PMC6722424; doi:10.1128/mSystems.00453-19)
Supplement: TABLE S4 [file mSystems.00453-19-st004.docx]

| **Gene** | **Primer 1** | **Primer 2** |
| --- | --- | --- |
| *rsmB* | GCGCGTGAATTAGCTTTAGTG | CCAACCGGCGATCTTTATCT |
| *ahpF* | CCGCTGAAGAAGCTGACTATT | ACGCTTAGCCGTCAATACAG |
| *moeB* | CGATCGTCAAGAAAGGGTACAT | TAACTTCCCAATGCTCCTACAC |
| *pcl1* | GAGACGGTTGTTCGGATTAAGTA | TCGCCATCAACGGAATCAA |
| *perR* | CGTCTGCGATAATTGTGGTAAG | GCCATGGACTTCGATGTTATTT |
| *rsiR* | TTATGCCAGAACAGCCGAATTA | TCGCCACCTAAACCATCAATAC |
| *sigH* | GACAAACTCAGGCTCACAAAC | AGACTCTGCCGGCAATAAA |
