# Supplementary material for: Complex Responses to Hydrogen Peroxide and Hypochlorous Acid by the Probiotic Bacterium Lactobacillus reuteri
Source: mSystems. 2019 Sep 3;4(5):e00453-19. doi: 10.1128/mSystems.00453-19 (PMC6722424; doi:10.1128/mSystems.00453-19)
Supplement: TEXT S1 [file mSystems.00453-19-s0001.docx]

**SUPPLEMENTAL METHODS**

**Inhibitory stress tolerance assays**

Wild-type *L. reuteri* were grown anaerobically in 5 ml of MEI-C broth overnight at 37°C. 15 ml of anaerobically equilibrated MEI-C in 16 x 125 mm Hungate tubes was inoculated with 300 µl of the overnight culture and grown anaerobically to mid-log phase (A_600_ = 0.35) at 37°C. Cells were then treated anaerobically with increasing doses of H_2_O_2_ or HOCl and incubation was continued at 37°C. A no stress control was included for each experiment. Cultures were mixed by inversion and A_600_ readings were recorded every hour after addition of stress treatment for 5 hours.

**NAD^+^ and NADH measurements**

Wild-type *L. reuteri* were grown anaerobically in 5 ml of MEI-C broth overnight at 37°C. 15 ml of anaerobically equilibrated MEI-C in 16 x 125 mm Hungate tubes was inoculated with 300 µl of the overnight culture and grown anaerobically to mid-log phase (A_600_ = 0.35) at 37°C. Cells were harvested at 0 min (without stress), 5 min, 15 min and 30 min after addition of 1.25 mM HOCl or 0.12 mM H_2_O_2_ by pelleting 1 ml aliquots and resuspending in 0.2 N NaOH with 1% DTAB (dodecyltrimethylammonium bromide). Samples were stored at -80°C for further processing. Serial dilutions of cultures were plated on MRS agar and CFU were counted after 24-48 hours of anaerobic incubation at 37°C. The Promega NAD^+^/ NADH Glo Assay kit was used to measure cellular NAD^+^/ NADH levels following the manufacturer’s protocol, which were then normalized to the number of CFU in each culture.

**RNA sequencing**

Wild-type *L. reuteri* were grown anaerobically in 5 ml of MEI-C broth overnight at 37°C. 300 µl of the overnight culture was used to inoculate four tubes containing 15 ml of anaerobic MEI-C broth, and the cultures were grown anaerobically at 37°C to mid log phase (A_600_ = 0.35). HOCl (1.25 mM) or H_2_O_2_ (0.12 mM) were added anaerobically to 3 of the 4 tubes and incubation was continued at 37°C. Cells (7 ml) were harvested at 0 min (no addition of stress; the tube which did not receive any oxidant), 5 min, 15 min and 30 min after addition of oxidants by mixing with 7 ml ice-cold isopropanol anaerobically to stop transcription, and then removing the samples from the anaerobic chamber and centrifuging at 1500 x g for 15 minutes at 4°C. The supernatant was discarded and the pellet was resuspended in the remaining isopropanol and transferred to 1.5 ml microfuge tubes. The cells were then centrifuged at 4000 x g for 10 minutes at 4°C. The supernatant was removed completely and the samples were stored at -80°C for further processing. Total RNA was extracted using the RiboPure™ RNA Purification Kit for bacteria (Ambion) following the manufacturer’s protocol. All RNA samples were DNAse treated with the optional DNAse treatment kit provided with the Ambion RiboPure Bacteria kit to remove any contaminating genomic DNA. Each stress treatment experiment was repeated in triplicate.

RNA-sequencing was performed on the Illumina HiSeq2500 using the Rapid Run v2 sequencing chemistry and flow cells as described by the manufacturer (Illumina Inc., San Diego, CA).  Briefly, the quality of the total RNA was assessed using the Agilent 2100 Bioanalyzer.  RNA with a RNA Integrity Number (RIN) of 7.0 or above was used for sequencing library preparation. We used the Agilent SureSelect Strand Specific mRNA library kit as per the manufacturer’s instructions (Agilent, Santa Clara, CA).  Cluster generation was performed according to the manufacturers recommendations for onboard clustering (Illumina).  Paired end 50 bp sequencing runs were used to allow for better alignment of the sequences to the reference genome.

**RNA-Seq Analysis**

The quality of FASTQ files were evaluated using FASTQC (1). Reads were trimmed at 50 bases and further QC filtering was performed using tool Trimmomatic with the following settings: CROP:50 LEADING:3 TRAILING:3 SLIDINGWINDOW:40:15 MINLEN:40 (2). Read mapping and transcript abundance measurement was performed using EDGE_pro which is designed for prokaryotic gene expression data analysis (3). EDGE_pro internally uses Bowtie2 for read mapping. Read count tables were imported into R for normalization, variance stabilization (rlog method) and differential expression analysis (multiple testing correction with the Benjamini-Hochberg method are provided, Bonferroni correction was used in the text) using DESeq2 (4). For differential expression analysis, the baseline samples for both the H_2_O_2_ and HOCl cultures were combined (n = 6 replicates for baseline samples); for all other groups (treatment/time-point combinations), there were three replicates each. Downstream analysis (PCA, clustered heat maps, visualizations) were performed using Matlab (The MathWorks, Natick MA, USA).

**Quantitative reverse transcription PCR**

300 µl of an overnight culture of *L. reuteri* cells was used to inoculate 15 ml of MEI-C medium. Cells were grown either anaerobically or microaerobically to mid log phase (A_600_ = 0.35) and then stressed with 0.625 mM, 1.25 mM, or 2.5 mM HOCl, or 0.06 mM, 0.12 mM, or 0.24 mM H_2_O_2_. Cells were harvested at 0 min (no treatment) and at 5, 15, or 30 min post treatment by adding 7 ml of culture to 7 ml of ice-cold isopropanol. Total RNA was extracted from frozen *L.* *reuteri* cells as described above for RNA sequencing. Reverse transcription was carried out to synthesize cDNA from mRNA using the SuperScript^TM^ IV VILO^TM^ master mix kit (ThermoFisher Scientific) following the manufacturer’s instructions. A no RT control was included for each reaction. Changes in gene expression relative to untreated controls were calculated using the ∆∆C_t_ method (5), with *rsmB* (16S rRNA) as an internal expression control.

**Survival and growth inhibition assays of mutant strains**

*L. reuteri* cells were grown to mid log phase (A_600_ = 0.35) in MEI-C medium either anaerobically or microaerobically. For bactericidal stress treatments, the cells were dosed with 1.5 mM H_2_O_2_ microaerobically and anaerobically, or with 2.5 mM or 7.5 mM HOCl under microaerobic and anaerobic growth conditions, respectively. 1 ml of cells was harvested at 0 min (no stress control), 20 min, 40 min and 60 min after addition of stress treatment. 3 mM sodium pyruvate was used to quench 1.5 mM H_2_O_2_; 25 mM sodium thiosulfate was used to quench HOCl. 10 fold serial dilutions of cells were made in PBS and appropriate dilutions were plated on MRS medium and incubated at 37°C under anaerobic conditions (BD Gas-Pak^TM^ EZ). CFU ml^-1^ were enumerated by counting colonies 24-48 hours after plating. Survival indices of mutant strains were calculated as the difference between the log_10_ CFU ml^-1^ of each mutant and the log_10_ CFU ml^-1^ of the wild type replicate carried out at the same time. For inhibitory stress treatments, stationary phase cells from overnight cultures were diluted 1:20 in 96-well plates in fresh MEI-C (200 µl) containing no oxidant or 2-fold dilution series of H_2_O_2_ (1 to 0.015625 mM) or HOCl (10 to 0.15625 mM) under anaerobic or aerobic conditions. Cultures were incubated 12 hours either anaerobically (in a Tecan Sunrise plate reader) or microaerobically (in a Tecan Infinite M1000 plate reader) at 37°C, measuring A_600_ every 30 min, shaking briefly before each measurement. Concentrations of oxidants that resulted in differences in growth between wild-type and mutants (0.0625 mM H_2_O_2_ and 1.25 mM HOCl) were selected and plotted as percent of wild-type A_600_ at the same time point under the same conditions.

**Statistical analyses.** Student’s t tests and two-way repeated measures ANOVA with Holm-Sidak’s multiple comparisons corrections were performed using GraphPad Prism version 8.0.1 for Macintosh, GraphPad Software, La Jolla California USA, ([www.graphpad.com](http://www.graphpad.com)).

**SUPPLEMENTAL REFERENCES**

1. Andrews S. 2012. FastQC: A quality control application for high throughput sequence data. Babraham Institute Project page: http://www.bioinformatics.bbsrc.ac.uk/projects/fastqc.

2. Bolger AM, Lohse M, Usadel B. 2014. Trimmomatic: a flexible trimmer for Illumina sequence data. Bioinformatics 30:2114-20.

3. Magoc T, Wood D, Salzberg SL. 2013. EDGE-pro: Estimated Degree of Gene Expression in Prokaryotic Genomes. Evol Bioinform Online 9:127-36.

4. Love MI, Huber W, Anders S. 2014. Moderated estimation of fold change and dispersion for RNA-seq data with DESeq2. Genome Biol 15:550.

5. Livak KJ, Schmittgen TD. 2001. Analysis of relative gene expression data using real-time quantitative PCR and the 2(-Delta Delta C(T)) Method. Methods 25:402-8.
